# 2025 Position statement on active outdoor play

**DOI:** 10.1186/s12966-025-01813-9

**Published:** 2025-09-25

**Authors:** Eun-Young Lee, Louise de Lannoy, Yeong-Bae Kim, Apoorva Rathod, Maeghan E. James, Olivia Lopes, Brianna Nasrallah, Anujah Thankarajah, Dina Adjei-Boadi, Maria Isabel Amando de Barros, Scott Duncan, Robyn Monro Miller, Lærke Mygind, Leigh M. Vanderloo, Po-Yu Wang, Mark S. Tremblay, AOP10 Steering Committee Group, Alethea Jerbine, Alison Amero, Arlene M. McGarty, Ashley McCurdy, Avril Johnstone, Carla Gull, Christine Alden, Colin Harris, Colleen A. Kiselyk, Dané Coetzee, David W. Chorney, Diego Augusto Santos Silva, Ellen Beate Hansen Sandseter, Erica Phipps, Erin Wentzell, Franz Plangger, Frederico Lopes, Gemma Goldenberg, Helen F. Dodd, Hyunshik Kim, Jane Cawley, Janet Omstead, Jasper Schipperijn, Javier Brazo-Sayavera, Justin J. Lang, Justin Jeon, Kamil Maciaszek, Kelly P. Arbour-Nicitopoulos, Kimberly Squires, Konstantina Rentzou, Kyoung June Yi, Lauren McNamara, Lauren Turner, Lisa M. Barnett, Lucy-Joy Wachira, Mairi Ferris, Mallory J. Donaldson, Mariana Brussoni, Marjaana Kangas, Mark Leather, Maxwell Hartt, Megan Zeni, Michael J. A. Down, Michelle Stone, Narayan Subedi, Nevin J. Harper, Nicola Kemp, Peter Bakalár, Peter Bentsen, Rachel A. Jones, Rachel Franz, Rachel Ramsden, Rebecca Forte, Richard Larouche, Rita Cordovil, Robert Wallis, Ryan Fahey, Salomé Aubert, Seiyeong Park, Shawnda A. Morrison, Sitong Chen, Son Truong, Søren Præstholm, Steph N. Dean, Stephanie A. Prince, Steven Lam, Suryeon Ryu, Susan Paudel, Suzanne Levenson, Tanya Halsall, Taru Manyanga, Tim Gill, Trish Tucker, Ulises Charles-Rodriguez, Valerie Carson

**Affiliations:** 1 School of Kinesiology and Health Studies, Queen’s University, Kingston, Canada; 2 Department of Sport Industry Studies, Yonsei University, Seoul, Korea, Republic Of; 3 Children’s Hospital of Eastern Ontario Research Institute, Ottawa, Canada; 4 Outdoor Play Canada, Ottawa, Canada; 5 Faculty of Kinesiology, Sport, and Recreation, University of Alberta, Edmonton, Canada; 6 Faculty of Medicine, University of Ottawa, Ottawa, Canada; 7 Department of Health Sciences, Carleton University, Ottawa, Canada; 8 Department of Population Medicine, University of Guelph, Guelph, Canada; 9 Department of Geography and Resource Development, University of Ghana, Legon, Accra, Ghana; 10 Instituto Alana, São Paulo, Brazil; 11 School of Sport and Recreation, Auckland University of Technology, Auckland, New Zealand; 12 International Play Association, Melbourne, Australia; 13 Center for Clinical Research and Prevention, Copenhagen University Hospital - Bispebjerg and Frederiksberg, Copenhagen, Denmark; 14 Research & Evaluation, ParticipACTION, Toronto Canada; 15 School of Occupational Therapy, Western University, London, Canada; 16 Department of Civic Education and Leadership, National Taiwan Normal University, Taipei, Taiwan

**Keywords:** Health promotion, Leisure, Nature, Nature-based solution, Planetary health, Physical activity, Recreation, Risky play

## Abstract

**Background:**

In 2015, the *Position Statement on Active Outdoor Play* was released in Canada, emphasizing the critical role of active outdoor play—with its risks—in fostering children’s healthy development. Building on this foundation, a 10-year update of the *Position Statement on Active Outdoor Play* (AOP10) was initiated to broaden its scope and impact, by encompassing all age groups and extending its reach conceptually and globally. Here we explain and present the new *2025 Position Statement*.

**Methods:**

Development of the 2025 Position Statement was informed by 18 rigorous literature reviews, a series of leadership group meetings, three rounds of draft AOP10 surveys, followed by extensive communication, translation, production, and dissemination activities.

**Results:**

The *2025 Position Statement on Active Outdoor Play* states: “Active outdoor play promotes holistic health and well-being for people of all ages, communities, and environments, and for our entire planet. It is critical given the multiple global challenges we face today (e.g., social and health inequities, climate change and digital addiction). Together, as a collective of the outdoor play sector, we recommend increasing opportunities for active outdoor play in all settings where people live, learn, work, and play. To achieve this, it is important to collaborate across sectors, settings, and societies to preserve, promote, and value equitable access to active play outdoors and in nature.” We also provide key evidence pertaining to the nine core themes that informed the development of the *2025 Position Statement* and offer recommendations across sectors, calling for multi-sectoral, multi-level collaborations. Across all three survey rounds, responses indicated strong support for the *2025 Position Statement* and its supporting content (Round 3: 93–98%). Comprehensive, proactive knowledge translation and dissemination plans were executed to maximize the reach and impact of the *2025 Position Statement*.

**Conclusions:**

The *2025 Position Statement* calls for systemic changes that prioritize equitable access to active outdoor play opportunities and aims to create healthier communities. Achieved through international collaboration and consensus, the *2025 Position Statement* aspires to connect, advise, inspire, and activate active outdoor play worldwide.

**Supplementary Information:**

The online version contains supplementary material available at 10.1186/s12966-025-01813-9.

## Background

In 2015, the *Position Statement on Active Outdoor Play* was released [1], calling for a fundamental shift in how society perceives and supports children’s active outdoor play in the Canadian context. Driven by scientific evidence from diverse disciplines—including public health, child development, early child education and care, and injury epidemiology— the statement emphasized the foundational importance of active outdoor play for children’s physical, mental, and social well-being [2]. The 2015 *Position Statement* also highlighted the need to balance risk and safety, recognizing that some degree of risk is essential for healthy child development [3]. A decade later, the world has changed in ways that make active outdoor play more important than ever. The COVID-19 pandemic, climate change, rising mental health concerns, and the increasing use of digital technologies have reshaped people’s lives, limiting and replacing opportunities for unstructured active outdoor play [1, 4–8].

Despite progress in outdoor play research, policy, and practice [1, 9, 10], many barriers to active outdoor play remain [11, 12]. Moreover, new challenges are emerging on a global scale. The widespread use of digital technologies and increasing prevalence of screen-based recreational activities is shifting people toward indoor, sedentary activities [4, 13–15]. Climate change is also playing a significant role, with rising temperatures, natural disasters, and unpredictable weather patterns impacting the feasibility of outdoor play in many regions [16–19]. Economic and geopolitical challenges in conflict zones [20–22], as well as socioeconomic disparities within and between countries [23–25] continue to create inequitable outdoor play opportunities, disproportionately affecting individuals from marginalized groups and communities on a regional- and global-scale.

Addressing these evolving challenges requires an integrated, cross-sectoral approach that merges urban planning, public health, education, and environmental sustainability efforts to ensure all individuals value and have access to safe, engaging, and healthy outdoor play experiences. In response to these challenges, an international leadership group came together to create an updated *Position Statement on Active Outdoor Play* (AOP10 project hereafter). The goal of this update was to reaffirm and expand upon the original statement’s principles while integrating new evidence pertaining to all age groups and addressing emerging global challenges. The updated *2025 Position Statement* aims to respond to the evolving issues faced by humanity, emphasizing the need for systemic action across various sectors—including policy and legislation, education and schools, urban and community planning, culture and society, media, and research and granting agencies. It calls for collective efforts to ensure that all individuals have equitable access to active outdoor play regardless of background or ability. Furthermore, it advocates for integrating active outdoor play into system-level strategies and initiatives that promote the health and well-being of the Earth’s ecosystems.

Now, more than ever, we must prioritize and protect the right to play outside for all. The overarching aim of the AOP10 project was to update the *2015 Position Statement on Active Outdoor Play* [1], expanding its scope to encompass all age groups, broader concepts, and reflect a global perspective [1]. The emphasis on *active outdoor play* is intentional, distinguishing it from broader concepts such as outdoor play, outdoor time, or outdoor learning [5]. While these terms are interconnected, they carry distinct meanings, and prioritizing *active outdoor play* highlights our consensus on its unique benefits for the health and well-being of humans and the planet. We used the international consensus definition of active outdoor play, defined as “voluntary engagement in activity that takes place outdoors, involving physical activity of any intensity, that is fun and/or rewarding and usually driven by intrinsic motivation [5]”. Accordingly, new to the *2025 Position Statement* is the recognition that active outdoor play applies across the lifespan. While terms such as ‘recreation’ and ‘leisure’ may be used more commonly for adults, they are included under the umbrella of active outdoor play, as long as they align with the above definition [5]. In line with this broader scope, the evidence reviews conducted to inform this update included studies on outdoor physical activity among adults and nature-based walking among older adults (e.g., [26]), while excluding studies focused on organized sport or structured exercise. To guide the inclusion and interpretation of data, we developed a typology of active outdoor play, presented in Table 1, which outlines the criteria used to define what constitutes active outdoor play for the purposes of this work. Operational definitions of active outdoor play and key terms used in and related to the *2025 Position Statement* are provided in Appendix A–Supplementary Table 1.

**Table 1:** Typology of active outdoor play

	**Considerations**		
	**General**	**Age**	**Country-Specific Context**
**Activity**			
Outdoor play	Play outdoors is generally assumed to be active; if outdoors, there is a higher likelihood of being active, even sporadically	Play is not considered something that adults do. A narrow definition of play excludes most age groups beyond children	
	Natural outdoor environments are more conducive to sporadic and spontaneous play for both children and adults alike; greenspaces or blue spaces provide organic opportunities for unplanned'play-like'activities		
Physical activity	Physical activity for the purposes of health improvement (e.g., walking-based intervention for obesity/type 2 diabetes management) is not considered play		
Sport	Sport as a career, elite-level sport, competitive sport is generally not considered play	Adult recreational sport leagues are considered play (especially if self-refereed), whereas child sport is not as it typically adult-directed and supervised	Within many African countries, sport is the primary way that children play, where it is often child-led and intrinsically motivated and/or voluntary
Recreation	Outdoor recreation as part of a job/career (e.g., hiking guide) is generally not considered play		
	Recreation needs to be unstructured to be considered play		
Active transport	Active transport is generally not considered play		
Play-based learning	Play needs to be explicitly mentioned (playful, play-based, etc.) in education-focused research to be included in order to have clarity on the intentionality within learning environments	Adventure camping as part of higher-level education is not considered play because it is not intrinsically motivated	In Denmark, guided play in early years settings is often used to initiate child-led play, and so is still considered play. In countries where the focus of early childhood education is much more on education, much less is considered play
	In education, there is a continuum of unstructured free play to teacher directed play, with guided play somewhere in the middle (e.g., setting up invitations for play where there is still opportunity for child-led play)		
	Active outdoor play in educational settings can be considered anything involving movement outdoors. This means loose parts, exploring as a class, actively engaging in a science activity, etc		
**Measurement Methods**			
Play-based evaluation		Early years research typically uses play methods to assess developmental stages; as this is not intrinsically motivated by the child, this is not considered play	
Proximity to outdoor recreation spaces	Proximity to an outdoor play/recreation space without discussing use of that space is generally excluded		

The objective of the present paper was to explain and present the updated *2025 Position Statement on Active Outdoor Play* and describe its release. 

## Methods

The development of the AOP10 project followed a multi-phase, participatory approach to ensure global representation, scientific rigor, and global relevance. The detailed preparatory process and methodology underpinning this report are documented in a separate methods-focused manuscript [27], which outlines the study design, data collection tools, and analytical approach in depth. The current manuscript focuses on interpreting the findings, highlighting key insights, and offering actionable recommendations.

### Reviews of the literature

Based on the conceptual framework developed for the AOP10 project (Appendix A–Supplementary Fig. 1) [27], 12 reviews were undertaken to provide key evidence on the core themes identified in the framework, along with six world region reviews. A full list of the reviews can be found in Supplementary Table 2 [27]. Methodological details of this phase is described in Sect. 2.5 of the accompanying AOP10 Methods paper [27]. The present paper provides a Summary of the key findings from the 18 reviews and relevant studies that informed the *2025 Position Statement*.

### AOP10 leadership group meeting

The 11-person AOP10 Leadership Group convened in Seoul, South Korea, December 6–9, 2024, for an intensive three-day working meeting. The meeting was co-organized by the AOP10 Executive Committee and the Department of Sport Industry Studies at Yonsei University. The primary objectives of this gathering were to 1) review and synthesize the latest evidence on active outdoor play based on the 18 reviews conducted, 2) engage in collaborative discussions, and 3) draft the *2025 Position Statement* and its supporting evidence statements. Through structured brainstorming sessions, the group worked to refine key messages, ensuring they reflected current research, emerging global challenges, and the needs of global communities. This in-person meeting provided an opportunity for interdisciplinary dialogue, fostering a shared vision to draft the *2025 Position Statement*.

AOP10 Steering Committee Group Consultation.

The AOP10 Steering Committee consisted of a diverse group of experts interested in outdoor play leadership, research, practice, and advocacy [27]. The first round of the consultation survey was conducted between January 10 and 21, 2025, and the second survey was circulated between February 14 and 21, 2025—via the online survey platform, REDCap [28] (BN, MEJ)—distributed to the AOP10 Steering Committee (N = 143). The purpose of engaging the AOP10 Steering Committee at this early stage of the consultation process was to create space for detailed feedback, identify areas requiring clarification or refinement, and collaboratively shape the tone, structure, and messaging of the *2025 Position Statement*.

Survey Round 1 gathered initial feedback on the Statement’s clarity, level of agreement with the content, and alignment with the AOP10 Steering Committee members’ understanding, research, and/or practice. Each question included a five-point Likert response scale (strongly agree, somewhat agree, neither agree nor disagree, somewhat disagree, strongly disagree), along with an open-text field for respondents to provide written feedback. We also gathered qualitative feedback for each section (i.e., Position Statement, Evidence, and Recommendations). In February 2025, Survey Round 2 was circulated with the Steering Committee after refinement based on the input from Round 1, ensuring alignment with current evidence and expert opinion pertaining to active outdoor play. The surveys distributed during Round 1 and Round 2 are available in Appendix B.

### Global collaboration group consultation

To further enhance the global relevance and cultural applicability of the *2025 Position Statement* draft, we circulated the further revised statements to the broader global community, leveraging the membership of the PLaTO-Net (Play, Learn, Teach Outdoors Network; https://www.outdoorplaycanada.ca/plato-net/) on March 21, 2025 via the online survey platform, REDCap [28] (BN, MEJ). This consultation was conducted in the six official languages of the United Nations (UN; i.e., Arabic, Simplified Chinese, English, French, Russian, and Spanish) to gather diverse perspectives and feedback from partners and interested parties worldwide. Initial translations for each language were generated using Chat Generative Pre-trained Transformer (ChatGPT), then reviewed and refined by two or three professionals actively working in the field, each fluent in English and one of the UN official languages. All reviewer-provided translations were consolidated, and consensus was reached. This multi-step process aimed to ensure that the final *Position Statement* reflects the needs, experiences, and cultural contexts of a broad and representative range of communities on a global scale. The survey was disseminated through the various networks of the AOP10 Leadership and Steering Committee members and followed a snowball sampling methodology to maximize reach and input. To minimize the risk of fraudulent responses in survey, multiple safeguards were employed including targeted recruitment, reCAPTCHA verification within the REDCap platform, use of survey access codes, and by refraining from posting a public survey link on social media platforms [29]. English and translated versions of the Global Collaboration Consultation Survey (Round 3) are available in Appendix B. The list of those who contributed to the translation process is presented in Appendix A– Supplementary Table 3.

### Analysis and integration

Both quantitative and qualitative feedback from the AOP10 Steering Committee and Global Collaboration Group consultation via three rounds of consultation surveys were systematically analyzed and integrated into the final version of the *2025 Position Statement*. The final statements were informed by both scientific evidence as well as expert knowledge and lived experiences shared via the surveys in an open-ended format from the AOP10 Steering Committee and the Global Collaboration Group (Appendix C). Immediately after each survey closed, two trainees (AT, BN) organized and summarized the responses. The Executive Leadership Team (E-YL, LDL, MST), together with the trainees (AT, BN, MJ), convened after each survey round to review both the quantitative results and qualitative feedback, which had been thematically grouped. Each written comment was carefully evaluated and triangulated (E-YL, LDL, MEJ, MST) to assess its validity and relevance to the refinement process. One trainee (AR) conducted an additional round of review and reorganization. Specifically, responses from all three rounds of the survey were systematically Summarized and restructured according to key thematic areas. This process aimed to improve comprehension, highlight patterns across rounds, and present the findings in a more coherent and accessible format for interested actors and readers of the final report. This was done in three stages: 1) reading all the qualitative comments for each section and highlighting common words and phrases across the comments, 2) grouping together all the comments with similar words and phrases and identifying possible themes, and 3) going over the themes and comments to see if there was further need for reorganization or regrouping. Where possible, these themes were also maintained across sections to be able to pinpoint overarching areas of concern and guide future works. For descriptive data, consensus was considered achieved a priori if 75% or more of valid respondents—respondents who provided complete data—indicated agreement (combining 'strongly agree' and 'somewhat agree') [30].

## Results

**Fig. 1:**
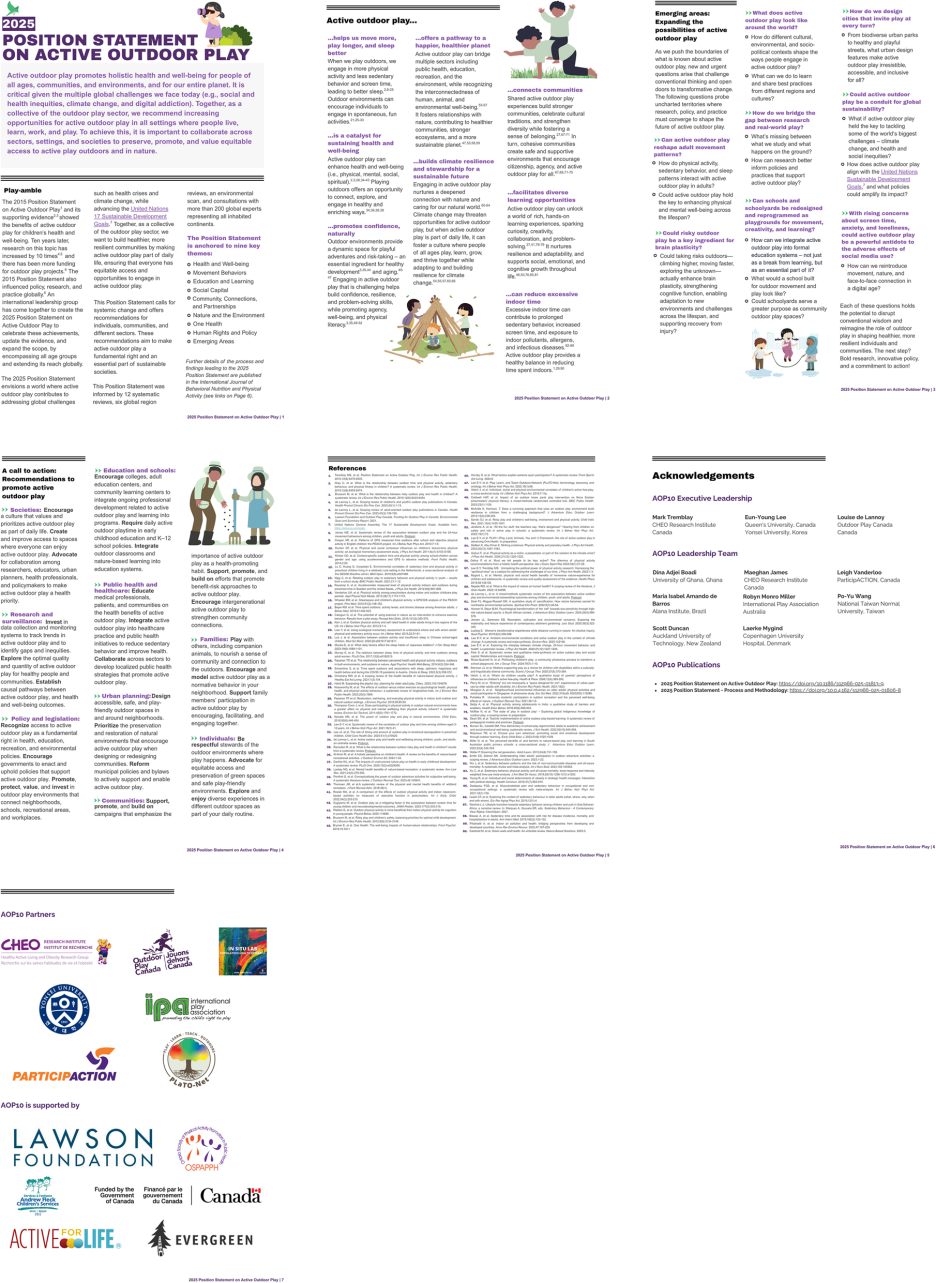
2025 Position statement on active outdoor play. Note: Visit https://www.outdoorplaycanada.ca/aop10/ to access the 2025 Position Statement in higher-resolution, print-quality format.

This section presents the *2025 Position Statement* and its eight key supporting evidence statements. The final *2025 Position Statement*, its supporting evidence statements, emerging areas, and recommendations are available in Fig. 1, with versions in each of the other UN languages are available at https://www.outdoorplaycanada.ca/aop10/. Below, the evidence that supported the development of the *2025 Position Statement* are explained further, followed by the summary of the AOP10 consultation process. Draft versions of the *2025 Position Statement* and evidence statements used during the three rounds of the consultation process are available in Appendix B.

### 2025 Position statement evidence

#### Evidence statement 1: movement behaviors

This evidence is consistent with and builds on the evidence used for the *2015 Position Statement* [2, 31]. In general, active outdoor play is associated with higher levels of physical activity, less sedentary behavior, including recreational screen time, and better sleep. Specifically, compared to being indoors, spending more time outdoors is associated with higher levels of physical activity among both children [2, 31–39] and adults [40–44]. Furthermore, being outside often reduces the likelihood of prolonged sitting and screen time for children [38, 39, 45] and adults [42, 44]. Outdoor settings provide diverse and dynamic stimuli that encourage movement—whether through walking, running, climbing, biking, wheeling, or simply exploring in urban greenery or nature—which not only helps expend energy but also supports better sleep quality and/or duration among children [31, 45, 46] and adults [47–49].

In addition to contributing to more physically active lifestyles, outdoor environments offer unique conditions that entice individuals to engage in spontaneous and enjoyable activities [49–54]. Natural features like trees, open fields, uneven terrain, and loose parts, or playground structures could inspire creative spontaneous active play, which is often unstructured and self-directed [11, 55]. The sensory experiences of being outdoors—sunlight, fresh air, varied textures, smells, and sounds—also contribute to emotional regulation and stress relief, which are linked to improved sleep patterns [46, 51, 52, 56]. Therefore, frequent engagement in active outdoor play on a regular basis helps individuals establish a healthy cycle of activity and rest throughout the day.

#### Evidence statement 2: health & well-being

This evidence is also consistent with and builds on the evidence used for the *2015 Position Statement* [2, 3, 57, 58]. A recent umbrella review [58] synthesizing evidence from six systematic reviews [3, 59–63] (Summarizing results from 381 published articles) reported overall favorable associations between active outdoor play and a broad range of physical, mental, social, and spiritual health and well-being outcomes, with partial support for causality. Of note, five reviews included in the umbrella review focused exclusively on active outdoor play in natural spaces. Nevertheless, these findings, in general, offer robust support for the multifaceted benefits of active outdoor play across the lifespan. A recent scoping review also highlighted that physical activity outdoors in nature provides greater health benefits compared to exposure to nature or physical activity alone [51].

Growing evidence suggests that the benefits of active outdoor play for children, regardless of where it takes place—either in open fields, playgrounds, urban centers, or public spaces—, extend beyond physical health, showing generally positive associations with social and mental health [3, 57] and executive functioning [64], and even mediates the negative impact of screen time on neurodevelopmental outcomes [65]. Furthermore, active outdoor play was associated with better cognition compared to physical activity done indoors [66]. In a recent systematic review on risky play and health outcomes among children aged 3–12 years [57], engaging in risky play—including rough-and-tumble play—was positively associated with physical health and fitness, as well as general gains in mental health, self-efficacy, self-regulation, and resilience. Taken together, these findings affirm that active outdoor play enhances health and well-being across physical, mental, social, and spiritual dimensions for people of all ages.

#### Evidence statement 3: risky play

This evidence is also consistent with and builds on the evidence used for the *2015 Position Statement* [3, 57]. Outdoor environments provide rich and dynamic spaces that foster playful adventures and healthy risk-taking—both of which are essential for development across the lifespan [5, 67, 68]. Among children, risky play—such as climbing, rough-and-tumble activities, or exploring unknown terrain—allows them to test their physical limits, manage fear, and develop risk perception skills that are critical for safety and resilience [3, 69–71]. These experiences are not just physically engaging; they also enhance children's confidence, resilience, and problem-solving capacities [3, 57, 72], while promoting their agency, well-being, and physical literacy [73–75]. This is particularly true when children are supported by nurturing social environments that support risky play [76]. On the other hand, emerging evidence suggests that “a mismatch between a child’s innate proclivity for risk-taking and the rise of ultra-safe and intensively parented play spaces” may have contributed to increased prevalence of anxiety among youth reported in recent data [77–79].

The value of active outdoor play—involving risky or thrill-seeking play [79]—extends into adulthood. Among adolescents and adults, activities like rock climbing, cliff diving, mountain biking, base jumping, and big wave surfing are often categorized as risky play [68]. The concept of risky play is rarely applied to older adults, whose play is often characterized as safe, slow, and stoic [80]. For older adults, play, in general, can be understood as a positive, creative, and stimulating behavior that is beneficial for social and physical well-being [81, 82], stress coping [83], personal development and instilling pro-environmental behavior [82]. It is noted that risky play, even for older adults, can be conceptualized as existing on a spectrum influenced by factors such as age, gender, ability, and personal preference [84, 85], which can include physically challenging activities that push personal comfort or ability levels, depending on individual capacity and interest. Regardless of the level of risks involved in play behavior, play-enabling outdoor spaces have been recognized as especially beneficial for older adults, as they foster social connection, reduce isolation, and support the maintenance of functional mobility and cognitive engagement [85]. Whether navigating childhood or aging, access to outdoor play environments that balance freedom with safety can be an important public health investment that fosters lifelong health and well-being.

#### Evidence statement 4: One Health

This evidence is newly incorporated to inform the *2025 Position Statement*. Active outdoor play can serve to connect benefits across sectors including, but not limited to, public health, education, recreation, and environment sectors to address global challenges. One Health is a multi-sectoral, integrated, transdisciplinary approach to addressing global health challenges, while recognizing the interconnectedness of animal, human, and environmental health [86]. The One Health approach fosters opportunities for individuals to move, learn, and thrive in environments that simultaneously promote biodiversity and ecological health through collective, cross-sectoral efforts [87]. This movement is gaining momentum worldwide, supported by a growing body of evidence and international collaborations that bridge cultural, geographical, and disciplinary boundaries [88–93]. As countries continue to grapple with complex and interconnected challenges of rising communicable and non-communicable diseases [94], active outdoor play offers a promising part of the solution by supporting healthier communities and fostering more resilient ecosystems across the lifespan and around the world [17, 26, 87].

Global momentum has been catalyzed through initiatives such as the AOP10 six world region reviews—representing Africa [92], Asia [90], Europe [88], Latin America and the Caribbean [93], Northern America [89], and Oceania [91]—highlighting how the *2015 Position Statement* has united researchers, practitioners, and policymakers in Canada and the urgent need to engage global communities for collaboration and partnership to address shared global challenges. Complementing this, a global scoping review exploring Indigenous knowledge systems related to outdoor play [95] highlighted the importance of place-based, relational worldviews in which outdoor play is not simply a recreational activity, but a deeply embedded practice that nurtures *wholistic* well-being [96–98]. In a similar vein, indigenizing outdoor play can be understood as integral to intergenerational learning, cultural continuity, decolonization, and shared responsibilities for conservation efforts and the advancement of Indigenous sovereignty [95–98].

#### Evidence statement 5: nature and the environment

This evidence is newly incorporated to inform the *2025 Position Statement*. Experiences in natural settings—whether through distance running, adventures, gardening, forest school programs or spiritual connections with the environment—can evoke personal transformation, eco-sensitivity, and a sacred appreciation for nature [99–103]. These embodied and emotional experiences have shown to play a role in shaping environmental attitudes and behaviors across the lifespan. As suggested by a recent review [103], regular outdoor play experiences can foster environmental stewardship, contributing not only to individual well-being but also to collective ecological consciousness. Therefore, when active outdoor play is integrated into everyday life, it can develop lifelong habits of caring for both personal and planetary health [5, 100, 104–106].

Caused by human industrial activity, climate change increasingly threatens opportunities for active outdoor play by altering landscapes, increasing exposure to extreme weather, and heightening health risks such as heat stress and air pollution [107]. These disruptions are known to reduce safe, accessible play opportunities for individuals and communities affected [17, 26]. At the same time, promoting active outdoor play is reported to offer a meaningful way to engage individuals and communities in climate adaptation and resilience building [26, 108–110]. As scholars in physical activity and public health have noted [111–113], the ostensibly apolitical framing of physical activity, including active outdoor play, can be strategically leveraged to engage with pressing global challenges, such as climate change. In this context, active outdoor play operates not only as a means of individual health promotion but also as a political act that aligns personal behavior with broader environmental and public health imperatives, aligning with the efforts being made primarily in the transportation sector for health co-benefits [114, 115].

Promoting local, low-impact outdoor play opportunities can help minimize travel-related emissions and foster stronger connections with nearby natural environments [26]. It is also important to acknowledge that not all outdoor play activities are inherently sustainable, the use of motorized recreational vehicles (e.g., snowmobiles and all-terrain vehicles) or the development and maintenance of large-scale, corporate-run, recreational resorts geared toward activities such as skiing/snowboarding, golfing, or scuba diving may contribute to environmental degradation and climate change [19, 26]. Recognizing these nuances is critical to guiding policies and practices that support both human and planetary health.

#### Evidence statement 6: social capital

This evidence is newly incorporated to inform the *2025 Position Statement*. Across age groups and settings, engaging in active outdoor play as a collective—whether through community gardens, public parks, schoolyards, or urban green spaces—has been shown to promote intergenerational connection, mutual understanding, and a shared sense of belonging [103, 116]. For children, active outdoor play is reported to facilitate the development of social skills, trust, safety, and empathy while also contributing to community-level social capital [72, 117, 118]. For adolescents, active outdoor play is reported to reduce social anxiety surrounding peer evaluation while enhancing social interactions [119], as well as allowing adolescents to acquire the social safety and support they need, particularly in rural and small-town settings [120]. Among adults and older adults, inclusive and play-enabling physical and social spaces have been shown to enhance perceptions of community connection, reduce social isolation, and encourage playful engagement with others [54, 81, 85, 121]. This is particularly relevant in urban settings where age-friendly design and inclusive planning are critical to ensuring all individuals, regardless of age or ability, can participate meaningfully in community life [54, 122, 123].

In turn, cohesive communities can create safe and supportive environments that promote citizenship, agency, and equitable access to active outdoor play [116]. Community cohesion and strong social networks are foundational to the development of public spaces that are perceived as safe, welcoming, and reflective of cultural values [122, 124]. When individuals feel a sense of ownership and representation in outdoor environments, it is reported that they are more likely to engage in activities that reinforce shared values and civic participation. This finding was particularly important for marginalized groups as it allows them to reshape power dynamics and improve access to play [117, 121, 122, 125]. Furthermore, evidence suggests that when adolescents and young adults have access to safe, culturally relevant, and socially supported outdoor play spaces, they report greater well-being and sustained engagement in physical activity [121, 126]. These findings collectively point to the potential role of active outdoor play as a vehicle for social inclusion, cultural celebration, and the strengthening of community resilience across the lifespan [116].

In nations grappling with the ongoing impacts of colonial legacies, a growing body of Indigenous scholarship and knowledge affirms that outdoor play must be understood not merely as an individual or developmental benefit, but as a practice deeply embedded in place-based, relational worldviews [95]. From this perspective, outdoor play is not separate from culture or community but is a living practice that strengthens ties to land, identity, and responsibility. These Indigenous frameworks challenge dominant paradigms by centering connection over consumption, reciprocity over extraction, and shared responsibilities for conservation [95–98]. Furthermore, among children and youth with disabilities, two recent reviews noted that the literature remains limited, with most studies focusing on supervised play in manufactured playgrounds rather than natural environments [123, 125]. These findings highlight a gap in understanding how natural outdoor settings—such as forests, parks, and open green spaces—may offer inclusive and developmentally supportive experiences for children with diverse abilities.

#### Evidence statement 7: education

This evidence is newly incorporated to inform the *2025 Position Statement*, to recognize the interconnectedness between active outdoor play and learning [5]. From early childhood through older adulthood, play—particularly in outdoor, unstructured, or semi-structured settings—serves as a dynamic foundation for lifelong learning that supports social, emotional, and cognitive growth [54, 59, 64, 127]. In early childhood, engaging with the natural environment through play is shown to enhance sustainable thinking through inquiry-based learning and environmental exploration, as well as opportunities for movement, imaginative engagement, and cooperative learning [128]. Outdoor learning environments, especially those that offer choice and adventure, promote social-emotional development by encouraging autonomy, empathy, and emotional regulation [129]. Nature-based play in school settings has also been associated with increased attention, engagement, and classroom readiness [130].

Active outdoor play-based learning continues well beyond early childhood [131]. School-aged children and youth can also benefit from adventure-based outdoor learning—such as rock climbing, mountain biking, orienteering, or group wilderness challenges—which fosters resilience, adaptability, and leadership [68, 73]. These experiences, especially when scaffolded within educational programs, can promote autonomy, risk negotiation, and collaborative problem-solving, which are central to adolescent development [127]. In adulthood, active outdoor play continues to serve learning opportunities [131, 132]. For example, a scoping review on older adults’ participation in outdoor adventure activities found that they not only developed new skills and competencies but also gained knowledge about nature and sustainability [132].

#### Evidence statement 8: indoor time

This evidence is consistent with and builds on the evidence used for the *2015 Position Statement* [1]. Prolonged indoor time often leads to more sedentary behavior and digital screen use, both of which are independently linked to increased risk for all-cause mortality, cardiovascular disease, and other chronic conditions [133–135]. These risks are compounded by limited opportunities for physical activity, particularly in the outdoors, as sedentary routines often dominate indoor environments in both occupational and domestic settings [136–138]. More alarmingly, higher levels of sedentary behavior have been associated with greater risks for disease incidence and premature death, even among individuals who meet recommended physical activity guidelines [139].

Beyond displacing opportunities for physically engaging and reinvigorating outdoor play, indoor environments may also expose individuals to a range of environmental health hazards, including indoor air pollutants, allergens, and infectious agents [140, 141]. These exposures can originate from various sources such as combustion appliances, volatile organic compounds from household products, tobacco smoke, dust mites, mold, and inadequate ventilation systems [141]. Indoor air quality has long been identified as a critical determinant of respiratory health, particularly among children, older adults, and individuals with pre-existing conditions. According to the World Health Organization [142], household air pollution is responsible for the premature deaths of over three million individuals annually. Among these, approximately 32% are attributed to ischaemic heart disease, 23% to stroke, 21% to lower respiratory infections, 19% to chronic obstructive pulmonary disease, and 6% to lung cancer. In children under the age of five, household air pollution accounts for nearly half of all pneumonia-related deaths. In addition to these established health outcomes, growing evidence indicates that household air pollution may also be associated with a range of other adverse health conditions, including low birth weight, stillbirth, asthma, ear and upper respiratory infections, tuberculosis, cataracts, and cancers of the nasopharynx, larynx, and cervix [140, 142].

It is important to acknowledge that environmental hazards are not confined to indoor spaces. Outdoor environments also contain pollutants—such as traffic-related air pollution, industrial emissions, and seasonal allergens—that pose risks to human health [143–145]. However, the magnitude of exposure risk indoors could be amplified by the sheer amount of time people spend within enclosed spaces. According to global estimates, individuals in both high- and low-income settings now spend approximately 90% of their time indoors—whether in homes, schools, workplaces, or transportation [140]. This prolonged time spent indoors can intensify the cumulative effects of indoor air quality on health over time, compared to that of ambient air pollution [140–142].

Therefore, while outdoor spaces are not without environmental risks (e.g., ambient air pollution, wildfire smoke), the predominance of indoor living environments in modern societies demands a critical public health response. In line with the *2015 Position Statement*, we maintain the position that regular engagement in active outdoor play can counterbalance the risks associated with extended time spent indoors daily [1]. Outdoor environments typically provide greater opportunities for movement and play, thereby facilitating higher levels of moderate-to-vigorous physical activity and helping individuals reduce sedentary time [2, 31]. Active outdoor play can also reduce cumulative exposure to indoor air pollutants and infectious diseases and offer restorative effects associated with natural environments—benefits that often surpass those gained through indoor activities [52, 146].

The *2025 Position Statement* (Fig. 1) also presents some key questions where interdisciplinary research, policy innovation, and practical implementation could emerge to shape the future landscape of active outdoor play. As the field of active outdoor play continues to evolve, emerging and pressing questions are challenging conventional paradigms, stimulating critical inquiry, and revealing new opportunities for advancing research and practice.

#### AOP10 Steering committee group consultation

To ensure that the *2025 Position Statement* reflected both current evidence and the collective vision of the AOP10 initiative, two iterative rounds of consultation were conducted with members of the global AOP10 Steering Committee. This internal consultation served as an essential step to build consensus among core contributors and ensure alignment on the foundational principles, framing, and direction of the *2025 Position Statement* and key evidence statements.

**Fig. 2:**
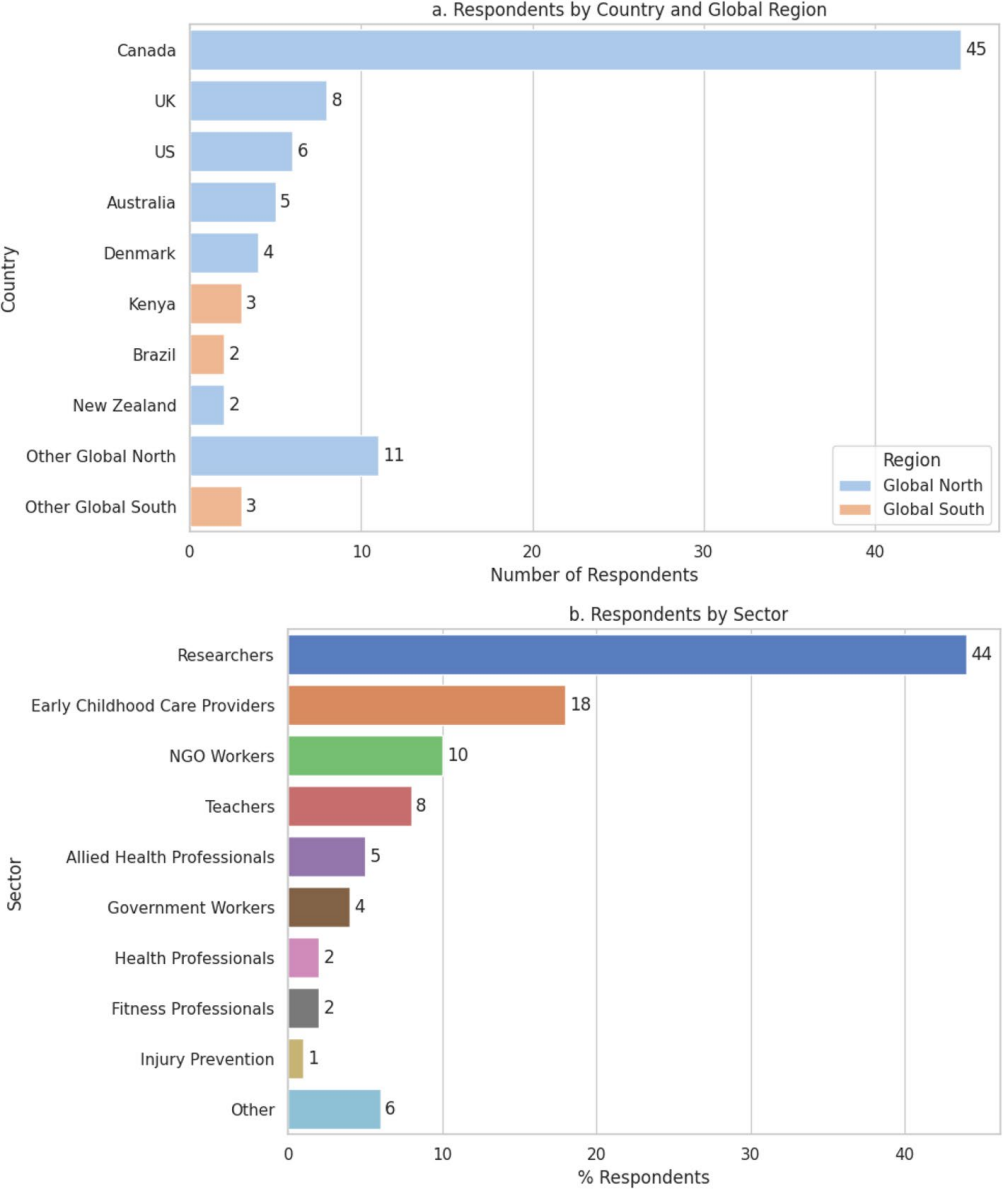
AOP10 Steering Committee Group that participated in consultation (*n* = 88). *Note*: Other Global North countries included Finland, France, Greece, Japan, Poland, Portugal, Serbia, Slovakia, Slovenia, Spain, Taiwan; Other Global South countries included Nepal and South Africa. Other sectors included childhood disability, play provision, urban planning and design, design, and funder. NGO: Non-governmental organization

A total of 139 members were invited via email to complete the Round 1 survey, of which we received 130 responses. Of those, 64% (84 respondents) provided complete responses. We also received qualitative feedback across the three major sections (i.e., Position Statement, Evidence, Recommendations). For Round 2, we received responses from 111 of the 139 members. Of those, 97 respondents completed at least 50% of the survey questions. Primary country of residence/workplace and sectoral representation (collected only during Round 1) of 88 respondents are illustrated in Fig. 2a and b, respectively.

Table 2 (Round 1 and Round 2) reports on three key measures—clarity, agreement, and alignment—for each section of the draft *2025 Position Statement*. In Round 1, 98% of valid respondents agreed the *2025 Position Statement draft* was clear and 95% agreed with its overall message. These figures remained high in Round 2, with 98% agreeing with the clarity and 98% agreeing with the message. The Play-amble was only introduced in Round 2 and received 98% agreement across all three measures based on valid respondents, indicating strong initial endorsement. For the Evidence sections, the largest increases from Round 1 to Round 2 were observed for Evidence 3: “Risky Play” on its clarity (78% to 98%), agreement (73% to 97%), and alignment (78% to 98%). Improvements were also observed for Evidence 1: “Movement Behaviors”, Evidence 2: “Health and Well-being”, and Evidence 7: “Learning”, which suggest that the revised contents in Round 2 strongly resonated with the Steering Committee and addressed previous concerns. Support for clarity (94% to 90%), agreement (95% to 89%), and alignment (96% to 92%) dropped slightly for Evidence 6: “Social Capital”; however, overall, strong consensus was achieved. Identified as an important thematic addition, “Emerging Areas” also received strong support in both rounds (95%−98%), showing strong interest in including forward-looking, novel areas of research.

**Table 2:** Assessment of the *2025 Position Statement* (AOP10) through a three rounds of consultation process

	**AOP10 Steering Committee Group**						**Global Collaboration Group**		
Survey Round	**Round 1**			**Round 2**			**Round 3**		
N (n)^a^	139 (84)			139 (76)			308 (182)		
	Clarity^b^	Agreement^b^	Alignment^b^	Clarity^b^	Agreement^b^	Alignment^b^	Clarity^b^	Agreement^b^	Alignment^b^
**Play-amble**	NA	NA	NA	98% (99%)	98% (99%)	98% (99%)	95% (97%)	95% (97%)	95% (97%)
**Position Statement**	98% (98%)	95% (94%)	NA	97% (97%)	96% (96%)	96% (97%)	95% (96%)	95% (96%)	93% (94%)
Evidence 1: Movement behaviors	90% (90%)	84% (86%)	90% (88%)	98% (99%)	96% (96%)	96% (96%)	97% (97%)	95% (96%)	97% (97%)
Evidence 2: Health and Well-being	93% (92%)	89% (88%)	95% (95%)	98% (99%)	97% (97%)	98% (99%)	97% (97%)	96% (97%)	97% (97%)
Evidence 3: Risky Play	78% (79%)	73% (75%)	78% (82%)	98% (99%)	97% (97%)	98% (96%)	98% (98%)	97% (97%)	96% (96%)
Evidence 4: One Health	89% (90%)	88% (89%)	92% (93%)	98% (95%)	96% (91%)	96% (93%)	97% (96%)	96% (97%)	94% (96%)
Evidence 5: Nature and the Environment	90% (90%)	90% (90%)	90% (92%)	95% (96%)	94% (96%)	95% (95%)	96% (97%)	95% (96%)	97% (96%)
Evidence 6: Social Capital	94% (94%)	95% (95%)	96% (96%)	90% (99%)	89% (96%)	92% (97%)	97% (97%)	96% (94%)	96% (95%)
Evidence 7: Learning	91% (92%)	89% (90%)	90% (92%)	98% (99%)	96% (97%)	96% (96%)	96% (97%)	94% (96%)	95% (94%)
Evidence 8: Indoor Time	NA	NA	NA	95% (91%)	92% (89%)	93% (91%)	96% (96%)	96% (96%)	95% (95%)
Emerging Areas	NA	97% (98%)	NA	98% (97%)	95% (99%)	97% (99%)	97% (97%)	97% (97%)	98% (98%)
Cultural and global considerations	NA	96%	NA	NA	NA	NA	NA	NA	NA
**Recommendations (Round 2 [detailed] and Round 3 [overall] only)**									
Overall	NA	NA	NA	NA	NA	NA	97% (97%)	97% (98%)	96% (96%)
Recommendations–Sectors	NA	NA	NA	89% (99%)	86% (99%)	89% (96%)	NA	NA	NA
Education and Schools	NA	NA	NA	97% (99%)	97% (96%)	96% (97%)	NA	NA	NA
Urban and Community Planning	NA	NA	NA	98% (97%)	98% (96%)	96% (97%)	NA	NA	NA
Public Awareness	NA	NA	NA	97% (99%)	93% (96%)	96% (97%)	NA	NA	NA
Media	NA	NA	NA	97% (96%)	95% (95%)	97% (95%)	NA	NA	NA
Research Monitoring	NA	NA	NA	98% (97%)	95% (96%)	96% (97%)	NA	NA	NA
Recommendations–Individuals	NA	NA	NA	94% (99%)	92% (97%)	93% (97%)	NA	NA	NA
Educators and Community Leaders	NA	NA	NA	97% (99%)	95% (97%)	96% (97%)	NA	NA	NA
Health Professionals	NA	NA	NA	97% (97%)	95% (96%)	96% (95%)	NA	NA	NA
Researchers	NA	NA	NA	98% (99%)	97% (99%)	97% (97%)	NA	NA	NA
Society	NA	NA	NA	97% (99%)	96% (97%)	95% (99%)	NA	NA	NA
**Is update important to… (Round 1 and Round 3 only)**									
	**Yes**	**Unsure**	**No**				**Yes**	**Unsure**	**No**
**Public Health**	98% (99%)	2% (1%)	0% (0%)	NA	NA	NA	99% (99%)	1% (1%)	0% (0%)
**Global and Planetary Health**	78% (76%)	20% (23%)	2% (1%)	NA	NA	NA	91% (91%)	6% (6%)	2% (2%)
**UN SDGs**	86% (87%)	13% (12%)	1% (1%)	NA	NA	NA	89% (89%)	10% (10%)	1% (1%)
**You and your job**	94% (95%)	5% (5%)	1% (0%)	NA	NA	NA	95% (95%)	4% (4%)	1% (1%)
**The country you reside in**	98% (98%)	2% (2%)	0% (0%)	NA	NA	NA	93% (93%)	5% (5%)	2% (2%)

*NA* Not available (the section was not included or evaluated in that round), *UN SDGs* United Nations Sustainable Development Goals

^a^N = Number of invitations sent; (n) = number of responses received

^b^The first percentage represents the total of"Strongly agree"and"Somewhat agree"responses based on the total valid responses (the number in parentheses represents the total of"Strongly agree"and"Somewhat agree"responses based on the respondents with complete data only)

The consultation data during this stage indicated a high degree of consensus on the core components of the *2025 Position Statement* and its supporting evidence statements, with most sections showing steadily high or increased support from Round 1 to Round 2. The extensive feedback helped fine-tune key content areas, particularly for Evidence 1: “Movement behaviors”, Evidence 3: “Risky Play” and Evidence 4: “One Health”*.* Finally, only asked during Round 1, nearly all Steering Committee members felt the update was important for public health (98%), their own work (94%), and their country (98%). A majority also recognized its relevance to global and planetary health (78% with 20% “Unsure” responses) and the UN Sustainable Development Goals (SDGs; 86% with 13% “Unsure” responses). During Round 2, clarity, agreement, and alignment were also assessed for both sector-specific and individual-targeted recommendations, which generally received a high level of consensus (ranging from 86 to 98%). Qualitative feedback highlighted concerns about repetition across different aspects of the recommendations. In response, we revised and streamlined the content for the subsequent survey round (see Appendix C for complete details of qualitative responses received).

#### Global collaboration group consultation

**Fig. 3:**
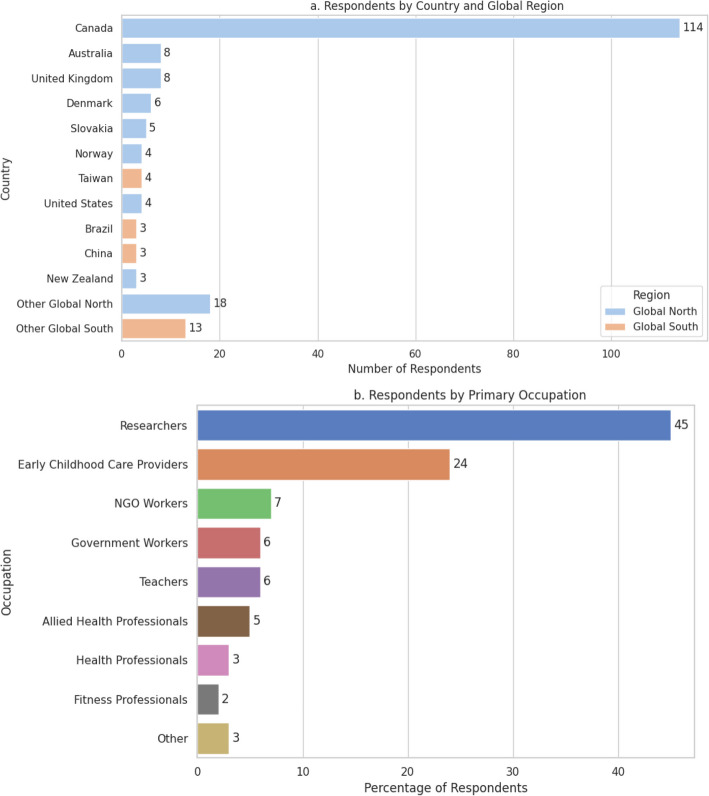
Global Collaboration Group that participated in consultation (*n* = 197; 38 countries). *Note*: Other Global North countries included Czeck Republic (*n* = 1), France (*n* = 2), Finland (*n* = 1), Germany (*n* = 2), Ireland (*n* = 1), Italy (*n* = 1), Japan (*n* = 2), Netherlands (*n* = 1), Poland (*n* = 1), Portugal (*n* = 2), Slovenia (*n* = 2), Spain (*n* = 1), Sweden (*n* = 1), Other Global South countries included Columbia (*n* = 1), Fiji (*n* = 1), Indonesia (*n* = 1), Kenya (*n* = 1), Mexico (*n* = 1), Nepal (*n* = 1), Philippines (*n* = 1), Qatar (*n* = 1), Serbia (*n* = 1), South Africa (*n* = 1), Turkey (*n* = 1), United Arab Emirates (*n* = 1), and Uruguay (*n* = 1). Other sectors included outdoor sports and education, horticulture/organic farming, teacher education, finance and banking, childcare licencing, or camp/outdoor education. NGO: Non-governmental organization

Survey Round 3 extended the consultation process to an even broader international audience through the Global Collaboration Group, with a specific emphasis on global relevance, cultural adaptability, and the incorporation of diverse knowledge systems. Respondents were asked to evaluate draft sections of the Statement, suggest additions or revisions, and provide written input. The Survey was open between March 21 to April 2, 2025. The survey link was opened 308 times, from which we received 221 responses. Primary country of residence/workplace and sectoral representation of 196 respondents are illustrated in Fig. 3a and b, respectively.

Similar to the previous consultation process, clarity, agreement, and alignment for each section of the draft *2025 Position Statement* were asked (Table 2, Round 3). The overall response rate was strong, reflecting global interest and engagement in shaping the *2025 Position Statement* and corresponding evidence base. Across all components (Play-amble, Position Statement, eight evidence statements, emerging areas, and recommendations), respondents reported 94%−98% on clarity, 94%−97% on agreement, and 93%−98% on alignment with their understanding, research, and/or practice.

In terms of the perceived importance of the AOP10 update across five domains (i.e., Public Health, Global and Planetary Health, UN SDGs, You and Your Job, and the Country You Reside In), respondents consistently recognized the importance of the AOP10 update across all domains. The perceived relevance to public health and individual professional roles remained high and stable, with 98–99% of respondents affirming its importance. Notably, there was a substantial increase in the perceived relevance to global and planetary health, rising from 78% in Round 1 to 91% in Round 3, accompanied by a sharp decline in uncertainty (from 20 to 6%). Perceptions of alignment with the UN SDGs also improved modestly (86% to 89%), with a corresponding decrease in the proportion of respondents who were unsure. While the AOP10 project was consistently viewed as important to respondents'countries of residence (98% in Round 1), a slight decrease was observed in Round 3 (93%), possibly reflecting the survey extended reach to additional regions.

## Discussion

The objective of this paper was to explain and present the updated *2025 Position Statement on Active Outdoor Play* and its release, along with the new evidence that has informed its development, actionable recommendations, and its validation process based on the phased consultation process that involved the AOP10 Steering Committee and Global Collaboration Group. The *2025 Position Statement* and its supporting content received strong support from the global community, indicating its broader global relevance while maintaining strong international and sectoral support. The system-focused recommendations and inclusion of new evidence areas were well-received, suggesting that the Statement is both timely and relevant across local and global contexts.

A notable expansion in the *2025 Position Statement* is the recognition of active outdoor play as an important right and opportunity across the lifespan. The importance of active outdoor play for children’s development is well-established [1–3]. Nevertheless, adults of all ages, often overlooked in discussions of play, also experience considerable health and social benefits from playful outdoor engagement, even if it may look different in its presentation from that of children. Research highlighted how outdoor play spaces, when designed to be age-friendly, inclusive, and culturally relevant, reduce social isolation, enhance cognitive function, and promote physical activity [54, 80, 81, 85]. Another key expansion in the *2025 Position Statement* is the framing of active outdoor play as a scalable solution that can be advanced through individual-level behavioral change. In the face of converging global crises—including climate change, sedentary lifestyles, zoonotic diseases, and social isolation—active outdoor play offers a timely and actionable entry point for multi-sectoral collaboration and public health promotion. This aligns well with the One Health approach, which recognizes the interdependence of human, animal, and environmental health [86]. Global momentum supporting this movement is rapidly growing. AOP10's six world-region evidence reviews have demonstrated how the *2015 Position Statement* and subsequent regional efforts have galvanized communities of research and practice [88–93]. These collective efforts highlight some global inequities and the need for further global collaboration, partnership, and knowledge exchange, as attempted through the AOP10 project, to advance policies and practices that support outdoor play in diverse cultural and ecological contexts.

In addition to the broader expansions, a critical advancement in the *2025 Position Statement* was the explicit and intentional centering of equity as a guiding principle for active outdoor play research, policy, and practice. The *2025 Position Statement* and associated recommendations call for a shift from viewing equity merely as access, to understanding it as a valued process of structural change. It is increasingly recognized that addressing individual-level barriers to participation is not enough to make sustainable, systems-wide change. To this end, the *2025 Position Statement* demands that environments, programs, and policies must be co-created with, and accountable to, those who have been historically excluded or marginalized. Equitable outdoor play spaces are not simply physical or logistical; they are culturally affirming, socially responsive, age inclusive, and structurally just spaces [54, 125]. Importantly, the *2025 Position Statement* intentionally takes an inclusive approach to defining outdoor play environments, recognizing that not all communities, particularly in low-resource or densely urbanized areas, have equitable access to safe, nature-rich spaces. Limiting the focus to such environments risks reinforcing existing inequities. By validating a broad spectrum of outdoor settings including open spaces, built environments, and informal urban areas, this Position Statement promotes a globally relevant and equity-informed vision for active outdoor play. Accordingly, we emphasize the value of all types of play spaces—from biodiverse parks and nature-rich environments to urban cul-de-sacs, manufactured playgrounds, and informal open spaces. Moreover, non-green natural landscapes such as snow-covered areas, deserts, coastal regions, and tundra are equally important and culturally meaningful sites for active outdoor play.

As part of the efforts to ensure the *2025 Position Statement* was more inclusive of diverse cultures and ways of knowing, Indigenous knowledge systems related to outdoor play have been intentionally integrated from the project’s start. A forthcoming global scoping review by McRae and colleagues [95] highlights the epistemological foundations of Indigenous outdoor play, emphasizing relational, land-based, and intergenerational paradigms [95–98]. Here, land-based includes those connected to nearby water bodies such as rivers, lakes, wetlands, and other bodies of water as part of a holistic relationship with the environment. These perspectives position outdoor play as a holistic and lifelong practice (‘a way of life’) that fosters intergenerational learning, cultural continuity, and shared responsibilities for conservation partnerships and Indigenous sovereignty [95–98]. Such relational worldviews challenge dominant Euro-Western constructs of play as discrete or child-centric and instead allows us to frame play as a practice of caretaking, healing, and social connection for sustainability [147]. Integrating Indigenous perspectives into outdoor play research, policy, and practice requires a decolonizing lens—one that centers Indigenous sovereignty, self-determination, and lived experience in shaping how, where, and why we play.

Consistent with the *2015 Position Statement* [1], we assert that the evidence base supports policies that facilitate, rather than restrict, opportunities for risky play [3, 57, 69]. This assertion does not entail a disregard for safety, but rather a recalibration of adult roles, including parents, guardians, childcare providers, and teachers, and an investment in built and natural environments that allow for hazard-free uncertainty [69]. The *2025 Position Statement* pivots from framing risky play “with its risks” to emphasizing the developmental value of risk-taking itself. This shift reflects both evolving evidence and an intentional reframing of risk as an opportunity for growth, resilience, and learning such as developing risk assessment skills [148], rather than a liability to be avoided. Even for adults, perceived risk is not the opposite of safety; it is an opportunity for developing stress-coping skills, fostering personal growth, encouraging pro-environmental behavior, and facilitating learning [82, 83]. During the consultation process, risky play emerged as a consistently contested concept. While some survey respondents expressed concerns regarding our strong advocacy for risky play for children due to issues around physical safety, liability, and potential injury, there is growing consensus within the research and practitioner communities that exposure to age- and ability-appropriate risk is not only safe when well-managed, but developmentally essential [69]. In recent work investigating risky or thrill-seeking play from an evolutionary perspective, with a monkey bar as a metaphor, which is typically considered as a public health hazard, indicated that children’s innate need for risky play is a result of selective pressures from the history of primate evolution [79]. Building on the momentum of the *2015 Position Statement* [1] and the subsequent support for risky play—including the Risky Outdoor Play Position Statement published by the Canadian Paediatric Society in 2024 [149] and Active Outdoor Play Position Statement from the Council of Chief Medical Officers of Health [150]—the *2025 Position Statement* continues, deliberately, to shift mindsets toward recognizing the developmental value of age- and ability-appropriate risk, “outweighing the occasional costs of injury [79].”

Another area of debate in the development of the *2025 Position Statement* centered on the framing of indoor environments. While it is true that excessive time indoors can contribute to sedentary behavior, reduced contact with nature and the community, and increased exposure to indoor pollutants and allergens, the intent is not to portray all indoor environments as inherently harmful. Rather, the *2025 Position Statement* calls for a more balanced and integrated daily routine of balanced indoor and outdoor experiences. Consistent with the *2015 Position Statement* [1], the *2025 Position Statement* reaffirms that outdoor environments offer unique restorative and holistic health benefits that surpass those provided by indoor activities [36, 48, 52, 53, 66]. While cautioning against reductive thinking that frames one environment as inherently'good'and the other'bad,'the *2025 Position Statement* deliberately calls for increased time spent outdoors to counterbalance the disproportionate amount of time people now spend indoors. With this, the focus should be on increasing access for active, nature-based outdoor play, especially in urban and underserved communities where access to safe, green outdoor spaces remains limited and unequal [151, 152]. Table 3 summarizes key benefits and potential risks associated with active outdoor play across the lifespan, alongside practical mitigation strategies. This synthesis highlights the complex interplay between promoting planetary health and well-being through active outdoor play while addressing safety and environmental challenges in diverse contexts.

**Table 3:** Benefits and potential risks associated with active outdoor play across the lifespan and practical mitigation strategies

Aspect	Benefits	Risks/Hazards	Mitigation Strategies
Physical Health	Enhances motor skills, physical literacy, fitness, resilience, and risk perception	Acute injuries from falls, bumps, scrapes	Design play spaces with varied challenges; supervision balanced to allow safe risk-taking
Cognitive & Emotional Health	Builds confidence, problem-solving skills, stress coping, and mental well-being across the lifespan	Anxiety from mismatch between innate risk-taking and overly safe play environments	Support nurturing social environments; promote autonomy and age-appropriate challenges
Social & Behavioral Health	Fosters agency, social connection, creativity, and pro-environmental behaviors, with potential behavioral spillover effects	Exposure to unsafe environments or social conflicts	Community engagement, education on social safety; inclusive and culturally affirming spaces
Environmental Health	Encourages environmental stewardship, eco-sensitivity, and lifelong connection to nature	Climate change impacts: extreme weather, environmental toxins, reduced access to safe play	Promote local, low-impact active outdoor play; climate adaptation strategies; minimize travel emissions
Exposure Considerations	Reduces sedentary indoor time and exposure to indoor air pollutants	Outdoor pollutants (air pollution, allergens, wildfire smoke)	Monitor air quality; plan play times to avoid high pollution; create diverse play environments including non-green
Across Lifespan	Supports healthy development in children, active aging, and functional mobility in older adults	Physical limitations or safety concerns, especially in young children, older adults, and people with disability	Adapt play environments to abilities; promote safe but stimulating challenges; encourage inclusive programming

As the evidence on active outdoor play evolves, we recognized that several urgent questions remain—for example, how can outdoor play be integrated into national and global strategies for climate resilience and adaptation? What does culturally safe, land-based play look like in different cultural and geographical settings? How can inclusive play be co-designed with communities facing structural inequities? These questions call for partnership and collaboration across disciplines and sectors, and for greater opportunities for leadership from Indigenous, racialized, and other equity-deserving communities. The future of active outdoor play lies in building systems that are not only health-promoting but also just, sustainable, and reflective of diverse ways of knowing, being, and playing. Based on our findings, we offer a set of recommendations aimed at driving systems-level change (Fig. 1)—targeting the societal actions, policies and legislations, institutional practices, health systems, infrastructures, and cultural norms that shape how, where, and for whom active outdoor play is possible. These recommendations move beyond individual-level behavior change to address the structural conditions necessary to create equitable, sustainable, and play-enabling environments across diverse communities.

### Strengths and limitations

This work presents the *2025 Position Statement*, which advances the field in several ways. One of its primary strengths is the expanded age range, expanded content, and global scope, building on the foundation of the *2015 Position Statement* [1]. By acknowledging the importance of active outdoor play not only for childhood but across the lifespan, the new *Position Statement* takes a more inclusive and holistic perspective. The Statement also situates active outdoor play within broader societal and environmental contexts, highlighting its potential to be part of a solution for emerging global challenges such as social and health inequities, climate change, and reduced access to natural spaces. In doing so, reviews conducted to directly inform the *2025 Position Statement* critically examined how active outdoor play can be integrated into daily life, policy, and practice. Collectively these reviews call for multi-sectoral, multi-level, and systems changes. A further strength lies in the rigorous, inclusive, and participatory development process. Three rounds of consultation were conducted with both the international AOP10 Steering Committee and the broader Global Collaboration Group, enabling iterative refinement and ensuring clarity, agreement, and alignment across diverse interested individuals and parties. Finally, a commitment to accessibility and inclusion for global community participation was demonstrated through the translation of the survey materials into the six official UN languages in an effort to enhance representation and empowerment from diverse linguistic and cultural backgrounds.

Despite its strengths, this work is not without limitations. The development process and participant representation remained largely Global North-centric, with a particularly strong Canadian influence. This geographic skew may affect the contextual relevance and applicability of the recommendations in underrepresented regions, especially across the Global South. In addition, notable evidence gaps persisted in the inclusion of research and practice related to outdoor play among individuals with disabilities [123, 125]. Future iterations of the Statement—especially those tailored for local or regional contexts—must intentionally center historically excluded voices and knowledge systems, particularly those rooted in Indigenous and non-Western worldviews. While broad consensus was achieved, consultation survey participants were primarily professionals already supportive of outdoor play, which may have limited opportunities for critical dissent or alternative perspectives that could have further enriched the final outputs. We hope the *2025 Position Statement on Active Outdoor Play* serves as a stepping stone towards overcoming these limitations.

### Future research directions

Although our efforts to develop the new *2025 Position Statement* with an expanded scope were succinct, we received valuable qualitative feedback that can guide future research to address current evidence gaps. Building on survey feedback, we propose five key research directions that will contribute to a deeper understanding of outdoor play across various populations and contexts.

In addition to this, the “Emerging Areas” and “Recommendations” sections of the Position Statement (Fig. 2) offer further insights into research and practical opportunities. A more complete list of research priorities, including those submitted by survey respondents, is provided in Appendix C.

First, future studies should explore how active outdoor play is perceived and experienced across the entire age spectrum. Research could examine the applicability of existing outdoor play frameworks to adults, older adults, and individuals with varying abilities. Understanding how different age and ability groups conceptualize and integrate outdoor play into their lives would help refine policies and interventions aimed at fostering outdoor engagement for all generations. Such an attempt was made [125] in one of the reviews conducted as part of the AOP10 Project; however, more deliberate research is needed to build upon these findings.

Second, future studies should examine how language used in promoting active outdoor play, such as phrases like “sit less,” impacts inclusion for people with disabilities or other diverse needs. Investigating how different terminologies influence perceptions and participation in active outdoor play across various populations would help create more inclusive communication strategies. Additionally, exploring how inclusive language can be integrated into play promotion for all abilities and age groups is essential for engaging underrepresented communities.

Third, further research is needed to understand how specific environmental design features—such as those related to natural elements, safety, and accessibility—affect active outdoor play. Studies could focus on how the quality and characteristics of outdoor spaces influence participation, particularly in urban areas where environmental conditions may limit opportunities. This research could also inform guidelines for creating more inclusive, stimulating, and accessible play environments, across different age groups.

Fourth, while the connection between active outdoor play and environmental stewardship is widely acknowledged as evidenced in a recent review [103], more research is needed to establish causality on the mechanisms of how outdoor play experiences contribute to long-term environmental engagement and vice versa, such as pro-environmental behavior and spillover effects into other sustainable behaviors [153–155]. Existing research has shown that engagement in outdoor play, particularly nature-based or risky play, can foster a sense of environmental responsibility and promote eco-friendly behaviors [106, 156, 157].

Fifth, future research should investigate the emerging hazards associated with outdoor play, especially in areas with high pollution or traffic, and those experiencing the impacts of climate change. This research would need to weigh the benefits of outdoor engagement against potential environmental hazards. Additionally, exploring how factors such as weather, geographical location, and societal norms influence the frequency and types of active outdoor play would provide important insights for tailoring interventions in diverse contexts.

Lastly, as we continue to advance into the digital age, it is important to explore the relationship between technology, including AI (artificial intelligence), and active outdoor play. Specifically, research could examine how digital tools, AI-powered platforms, and robotics can be utilized to enhance or facilitate outdoor engagement. In particular, the integration of robotics in physical rehabilitation, such as for post-stroke patients [158], offers promising opportunities to support mobility and movement in outdoor settings. Investigating how these technologies can be used to encourage participation in active outdoor play and rehabilitation could provide valuable insights into improving health outcomes and promoting greater engagement in nature-based activities.

### Knowledge Translation and Mobilization (KTM)

Tenets of implementation science and KTM emphasize the importance of sustained interaction and mutual engagement among researchers, decision-makers and practitioners [159, 160]. This reflects the emerging acceptance that traditional dissemination approaches, which typically involve one-way communication, have largely been ineffective at changing policy and practice. In our effort to maximize impact, we considered KTM early in the AOP10 project. Planned KTM activities were deliberate and dynamic between knowledge users and the research team to discuss the results and present the overall findings. This was performed alongside more traditional dissemination practices (end-of-grant KTM) such as presentations at scholarly meetings and peer-reviewed publications.

Our integrative KTM plan followed three main steps.

**Fig. 4:**
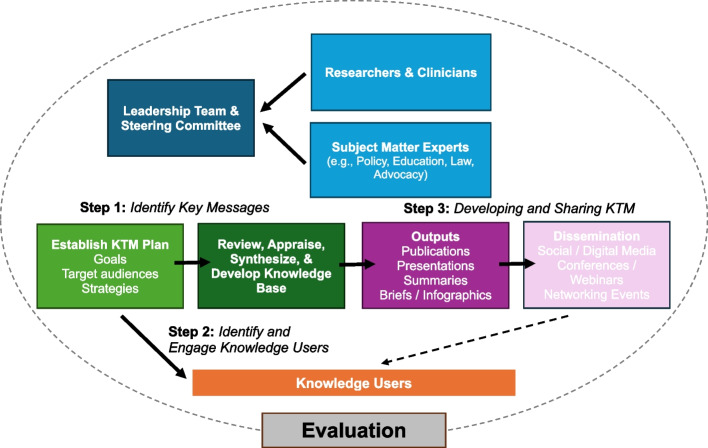
Knowledge Translation and Mobilization Model for the *2025 Position Statement* (AOP10) Project

- Stage 1: Identifying key messages – The proposed work generated evaluation findings and practical information. Ongoing challenges will be in translating these findings into information that will bring about systemic changes in practices and programs. The Steering Committee will play a key role in this process.
- Stage 2: Identifying and engaging knowledge users – The AOP10 Leadership Team worked with the Steering Committee to identify target audiences for KTM products (e.g., partners in research, education, public health, environment sectors).
- Stage 3: Developing KTM products – The Leadership Team developed a suite of assets that are action-oriented and tailored to our knowledge users across research, policy, and practice sectors. To create welcoming and inclusive material, a Sex- and Gender-Based Analysis Plus lens [161] will be applied across all materials. Further, the *2025 Position Statement* is available in all six UN official languages (i.e., Arabic, Chinese, English, French, Russian, and Spanish). See Fig. 4 for a high-level overview of the KTM Logic Model for the AOP10 project.

The *2025 Position Statement on Active Outdoor Play* was officially launched on September 25, 2025 at the 2025 Breath of Fresh Air Summit, marking a key milestone in the project’s public engagement and policy influence efforts. Promotional activities will continue to be carried out through several key platforms and events. ParticipACTION, a Canadian not-for-profit organization that promotes healthy living and physical activity across the country, provided support for the project’s outreach and promotion efforts, leveraging their established communication channels and audience engagement strategies to amplify reach. Features about the *Position Statement*, shared as blog posts, infographics, whiteboard videos, and the full-length and summary report are included on the Outdoor Play Canada website, and targeted posts made across Outdoor Play Canada and PLaTO-Net’s social media channels (e.g., Instagram, Facebook, LinkedIn, and BlueSky). Similar amplification efforts were made through the Leadership Team and Steering Committee connections and networks around the world. These KTM efforts aim not only to share research findings but also to strengthen intersectoral collaboration, build capacity across sectors, and influence future policy and practice.

As the AOP10 project was conceived to elevate the visibility and influence of the outdoor play sector, its dissemination strategies reflect a strong commitment to multi-sectoral collaboration and knowledge mobilization. Per the team’s well-established networks nationally and internationally, we believe the proposed KTM activities will maximize the reach and uptake of this work.

### Commitment to Justice, Equity, Diversity, and Inclusion (JEDI)

The JEDI principles have guided this project since its inception in 2023, shaping both the composition of the AOP10 Leadership Group and our intentional expansion from the *2015 Position Statement* [1] to include all ages and a global community. In developing the main Position Statement, we also made a deliberate decision to prioritize equity rather than adopt broader terms like inclusion, as suggested by some survey respondents. This choice is rooted in our commitment to systemic change and accountability to those historically excluded or marginalized from outdoor play opportunities, which also have been reflected in our sector-directed recommendations. While inclusion is important, it often centers on inviting individuals into existing systems without questioning or altering the structures themselves, but rather focussing on creating a welcoming environment for everyone [162]. In contrast, equity more directly calls for the dismantling of systemic barriers, a rebalancing of power, and an honest reckoning with historical injustices to ultimately achieve fair outcomes [162]. It requires that environments, policies, and practices be fundamentally (re)assessed, implemented, and reimagined to foster just, sustainable participation for all in varying contexts [163–166]. Access to active outdoor play and nature must go beyond physical and/or logistical availability; it must be about creating culturally affirming, socially responsive, age- and ability-inclusive, and structurally just spaces. In our KTM efforts, we also took intentional steps to ensure our processes and outputs are inclusive, accessible, and representative of diverse experiences and worldviews. For example, the global survey and KTM materials are available in all six official UN languages and adhere to international accessibility standards to help broader engagement and equitable access to information. We will continue to uphold our commitment in all future KTM efforts to ensure that JEDI principles remain at the core of our work as we move forward.

## Conclusions

This paper serves as both a reflection on the progress made over the past decade since the *2015 Position Statement* and a roadmap for the future. As we navigate a world increasingly shaped by climate change, sedentary, indoor-centered lifestyles, social fragmentation, and widening inequities, active outdoor play emerges as more than just a lifestyle choice but an important strategy for advancing the holistic health and well-being of human, animals, and the planet. The *2025 Position Statement* renews our collective commitment to the right to play for all, while focusing on improving equity, recognizing that access to nature and quality outdoor spaces remain unequal, and calls for rethinking the systems and structures that sustain exclusion and disadvantage. Through this renewed commitment, we are advocating for a world where all individuals can move freely, play safely, and thrive in nature-rich environments amid complex challenges we face as a species. Furthermore, the *2025 Position Statement* calls on us to engage with diverse knowledge systems, particularly Indigenous perspectives, that have long recognized outdoor play as land-based, relational, and intergenerational. We must also embrace a balanced, lifelong, and community-centered approach to active outdoor play. Active outdoor play is not only essential for the holistic health and well-being of individuals but could also serve as an important mechanism for addressing environmental and societal challenges we face. Aligning with the recommendations made, our future depends not just on research and policy, but on partnership, collaboration, and collective action. The time to create equitable, inclusive, and play-enabling environments for all, across all settings and generations, is now.

## Electronic Supplementary Material

Below is the link to the electronic supplementary material.

Appendix A

Appendix B

Appendix C

## Data Availability

No datasets were generated or analysed during the current study.

## Abbreviations

AI: Artificial Intelligence
AOP10: 10-Year update of the 2015 Position Statement on Active Outdoor Play
ChatGPT: Chat Generative Pre-trained Transformer
JEDI: Justice, Equity, Diversity, and Inclusion
KTM: Knowledge Translation and Mobilization
PLaTO-Net: Play, Learn, and Teach Outdoors Network
SDGs: Sustainable Development Goals
UN: United Nations
