# Correction to: 2025 position statement on active outdoor play

**DOI:** 10.1186/s12966-025-01854-0

**Published:** 2025-11-20

**Authors:** Eun-Young Lee, Louise de Lannoy, Yeong-Bae Kim, Apoorva Rathod, Maeghan E. James, Olivia Lopes, Brianna Nasrallah, Anujah Thankarajah, Dina Adjei-Boadi, Maria Isabel Amando de Barros, Scott Duncan, Robyn Monro Miller, Lærke Mygind, Leigh M. Vanderloo, Po-Yu Wang, Mark S. Tremblay, AOP10 Steering Committee Group

**Affiliations:** 1 School of Kinesiology and Health Studies, Queen’s University, Kingston, Canada; 2 Department of Sport Industry Studies, Yonsei University, Seoul, Republic Of Korea; 3 Children’s Hospital of Eastern Ontario Research Institute, Ottawa, Canada; 4 Outdoor Play Canada, Ottawa, Canada; 5 Faculty of Kinesiology, Sport, and Recreation, University of Alberta, Edmonton, Canada; 6 Faculty of Medicine, University of Ottawa, Ottawa, Canada; 7 Department of Health Sciences, Carleton University, Ottawa, Canada; 8 Department of Population Medicine, University of Guelph, Guelph, Canada; 9 Department of Geography and Resource Development, University of Ghana, Legon, Accra Ghana; 10 Instituto Alana, São Paulo, Brazil; 11 School of Sport and Recreation, Auckland University of Technology, Auckland, New Zealand; 12 International Play Association, Melbourne, Australia; 13 Center for Clinical Research and Prevention, Copenhagen University Hospital - Bispebjerg and Frederiksberg, Copenhagen, Denmark; 14 Research & Evaluation, ParticipACTION, Toronto, Canada; 15 School of Occupational Therapy, Western University, London, Canada; 16 Department of Civic Education and Leadership, National Taiwan Normal University, Taipei, Taiwan

**Correction : Int J Behav Nutr Phys Act 22, 117 (2025)**

**https://doi.org/10.1186/s12966-025-01813-9**

Following publication of the original article [1], the authors reported an error in the name of one of the contributors on the AOP10 Steering Committee Group list. It currently appears as “Jerbine” and should be corrected to “Jerebine.”

## References

[1] Lee EY, de Lannoy L, Kim YB et al. 2025 Position statement on active outdoor play. Int J Behav Nutr Phys Act 22, 117 (2025). 10.1186/s12966-025-01813-9 10.1186/s12966-025-01813-9 PMC12462132 40999404

