# Supplementary material for: 2025 Position statement on active outdoor play
Source: Int J Behav Nutr Phys Act. 2025 Sep 25;22:117. doi: 10.1186/s12966-025-01813-9 (PMC12462132; doi:10.1186/s12966-025-01813-9)
Supplement: Supplementary file 1 — Appendix A [file 12966_2025_1813_MOESM1_ESM.pdf]

2025 Position Statement on Active Outdoor Play

Appendix A: Supplementary Files

Table of Contents

Supplementary Figure 1. AOP10 Conceptual Framework. 2

Supplementary Table 1. Operational definitions of key terms by themes. 3

Supplementary Table 2. List of reviews (N = 18) conducted to inform the 2025 Position Statement. 9

Supplementary Table 3. List of individuals who contributed to the translation of the Global Collaboration Consultation Survey and final 2025 Position Statement. 12

References 13

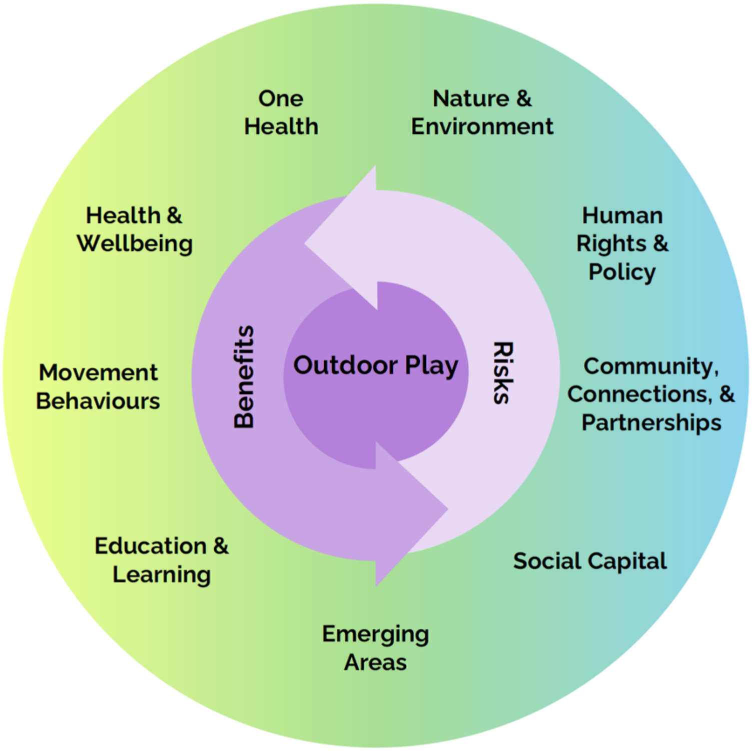

Supplementary Figure 1. AOP10 Conceptual Framework.

Note. Detailed development process and description of the framework is available in de Lannoy et al. [1]

Supplementary Table 1. Operational definitions of key terms by themes.

Term	Operational definition
**Active Outdoor Play**	“Voluntary engagement in activity that takes place outdoors, involving physical activity of any intensity, that is fun and/or rewarding and usually driven by intrinsic motivation.” [2]
Risky play	“Voluntary engagement in activity that is thrilling and exciting, which involves uncertainty, unpredictability, and varying degrees of risk-taking.” [2]
Outdoor play	“Voluntary engagement in activity that takes place outdoors, that is fun and/or rewarding and usually driven by intrinsic motivation.” [2]
Outdoor time	“Time spent outdoors” [2]
Outdoor activity	Leisure, recreational, educational, occupational, and/or health-enhancing activity engaged in the outdoors [2]
Recreation	Activities undertaken for enjoyment, relaxation, or personal development during one's discretionary time. These activities can range from sports and outdoor adventures to arts and cultural engagements, all contributing to individual well-being and social cohesion [3].
Leisure	Engaging in activities voluntarily chosen for their inherent satisfaction, not due to external pressures, obligations, or rewards [3].
**Health and Well-being**	
Mental Health	“A state of well-being in which an individual realizes their own abilities, can cope with the normal stresses of life, can work productively and is able to make a contribution to their community.” [4]
Social Health	“adequate quantity and quality of relationships in a particular context to meet an individual’s need for meaningful human connection.”[5]
Spiritual Health	Spiritual health can be defined or interpreted differently across different countries and/or by different cultural or religious groups [6], nevertheless, in this work, spiritual health is defined as “a multi-faceted and dynamic concept that encompasses a sense of connection to something beyond oneself or an awareness of a greater dimension, characterized by personal identifiable values related to self, others, nature, life and whatever one considers to be most important.”[7]
Holistic/Wholistic Health	Holistic or wholistic health describes an approach that considers the interconnectedness of physical, mental, emotional, social, and spiritual health and well-being, but "wholistic" is considered as a more inclusive term, particularly in contexts like Indigenous health [8].
Harm	Physical or mental damage or injury: something that causes someone or something to be hurt, broken, made less valuable or successful [9].

Hazard/Danger	A source of harm that is not obvious, such that the potential for injury is hidden [10-12].
Risk	A probability of harm [13].
Resilience	The ability to withstand, adapt to, and recover from adversity or stress—often with the potential to emerge stronger. It is not merely about toughness, but about flexibility, adaptability, and the capacity to navigate and grow through change and disruption [14, 15].
**One Health**	"One Health is an integrated, unifying approach that aims to sustainably balance and optimize the health of people, animals and ecosystems." (WHO, n.d.)<br><br>Increasingly, "there is an emphasis within the One Health scientific community on the need for widening the One Health concept to encompassing not only human and animal health, but also biodiversity, ecology, climate change, agricultural systems, and various social sciences." [16, 17]
Planetary health	"...the achievement of the highest attainable standard of health, well-being, and equity worldwide through judicious attention to the human systems—political, economic, and social—that shape the future of humanity and the Earth's natural systems that define the safe environmental limits within which humanity can flourish." [18]
EcoHealth (Ecosystem Approaches to Health)	"EcoHealth is committed to fostering the health of humans, animals, and ecosystems and to conducting research which recognizes the inextricable linkages between the health of all species and their environments. A basic tenet held is that health and well-being cannot be sustained in a resource depleted, polluted, and socially unstable planet." [19]
Environmental health	Environmental health has been defined in many different ways [20]. According to Frumkin, environmental health is "the field of public health that addresses physical, chemical, biological, social, and psychological factors in the environment. It aims both to control and prevent environmental hazards and to promote health and well-being through environmental strategies [20].
Global health	"...an area for study, research, and practice that places a priority on improving health and achieving equity in health for all people worldwide. Global health emphasises transnational health issues, determinants, and solutions; involves many disciplines within and beyond the health sciences and promotes interdisciplinary collaboration; and is a synthesis of population-based prevention within individual-level clinical care." [21]
Global health equity	"...mutually beneficial and power-balanced partnerships and processes leading to equitable human and environmental health outcomes on a global scale." [22]
**Nature and the Environment**	

Nature	“everything in the physical world that is not controlled by humans, such as wild plants and animals, earth and rocks, and the weather.”[23]
Climate change	"A change in the state of the climate that can be identified (e.g., using statistical tests) by changes in the mean and/or the variability of its properties, and that persists for an extended period, typically decades or longer. Climate change may be due to natural internal processes or external forcings such as modulations of the solar cycles, volcanic eruptions, and persistent anthropogenic changes in the composition of the atmosphere or in land use.” [24]
Climate resilience	“The ability to anticipate, adapt to, and recover from the adverse impacts of climate change.” [25]
Health co-benefits	A recognition that actions taken to reduce greenhouse gas emissions and adapt to climate change can simultaneously improve public health [26].
Pro-environmental behavior	“The commission of acts that benefit the natural environment (e.g., recycling) and the omission of acts that harm it (e.g., avoid air travel).” [27]
Environmental stewardship	“Diverse actions as creating protected areas, replanting trees, limiting harvests, reducing harmful activities or pollution, creating community gardens, restoring degraded areas, or purchasing more sustainable products.”[28]
Connectedness to nature	“‘the extent to which an individual includes nature within their cognitive representation of self.” [29]
Sustainability	we use the term sustainability in multiple contexts throughout the paper. To support a nuanced understanding, we refer readers to the following sources outlining key archetypes of sustainability [30, 31].
**Human Rights and Policy**	
Equity	“…achieving fair outcomes, recognizing diversity, and addressing inequality through intervention.” [32]
Diversity	“…the welcoming and embracing of difference, in relation to social demographics as well as a diversity of perspectives and ideas.” [32]
Inclusion	“…the idea that it is not enough to invite a variety of people into institutions…. Inclusion is fostering an environment and culture that is welcoming and supports diverse individuals and/or groups of people, and may also require concrete changes (e.g., accommodations to address physical and social barriers to inclusion).” [32]
Access/Accessibility	Access is not only about removing physical, social, or attitudinal barriers, but also about transforming structures to proactively center the needs and voices of those historically excluded or marginalized.

	Accessibility refers to the intentional design, planning, and implementation of environments, policies, programs, and practices to ensure that all individuals—particularly those from historically marginalized or underserved communities—can equitably participate, engage, and benefit without facing physical, systemic, social, or cultural barriers [33].
**Community, Connections, and Partnerships**	
Decolonization	In the context of settler colonial states—where human habitation began with Indigenous peoples and was followed by European settlers who arrived with the intent to claim the land (e.g., Australia, Canada, New Zealand, USA)—colonization typically refers to Eurocentric systems and worldviews [34]. Decolonization, in this context, involves dismantling colonial structures and supporting Indigenous resurgence, which refers to Indigenous peoples reclaiming and revitalizing their cultures, lands, languages, relationships, and health—both independently and in collaboration with non-Indigenous allies [35-37].<br><br>In other contexts, decolonization may more broadly refer to a shift away from Western European-derived ways of being, knowing, believing, and doing—ways that are often positioned, implicitly or explicitly, as the normative or superior standard. It involves recognizing and treating other worldviews and knowledge systems—frequently marginalized or framed as “alternative” or less legitimate—as equally valid and valuable [34].
Reconciliation	The Truth and Reconciliation Commission (TRC) of Canada defines reconciliation as “establishing and maintaining mutually respectful relationships between Indigenous and non-Indigenous peoples, requiring awareness of the past, acknowledgment of harm, atonement, and action to change behavior.” [35]
Indigenization	In the context of settler colonial states—where human habitation began with Indigenous peoples and was followed by European settlers who arrived with the intent to claim the land (e.g., Australia, Canada, New Zealand, USA)—, Indigenization refers to the intentional process of incorporating Indigenous perspectives, knowledge systems, and ways of being into institutions and practices [35].<br><br>United Nations define Indigenization as “making something more native; transformation of some service, idea, etc. to suit a local culture, especially through the use of more indigenous people in administration, employment, etc.” [38]

Relationality	"In Indigenous worldviews, relationality is based on the principle that everything is interwoven, therefore, the idea that a person or an entity could exist outside the boundaries of this network is deemed unimaginable."[39, 40]
**Social Capital**	"Features of social organizations, such as networks, norms and trust that facilitate action and cooperation for mutual benefit." [41]
Social cohesion	"The presence of strong social bonds that bridge divisions in society, and the lack of conflict in a society. The sense of solidarity among members of a community." [42]
Social support	"The social resources that persons perceive to be available or that are actually provided to them by non-professionals in the context of both formal support groups and informal helping relationships." [43]
Social connectedness (sense of belonging, sense of community)	"An individual's perception of belonging, emotional connection, and mutual support within a group, neighborhood, or society." [44]<br><br>It includes feelings of membership, influence, shared emotional connections, and the fulfillment of needs within a community and the quality of social ties [45].
Social isolation	"Lacking social connection" in which "the umbrella term social connection represents a multifactorial construct that includes structural, functional, and qualitative aspects of social relationships." [46]
Safety concerns (perceived safety)	"An individual's subjective perceptions of how (un)safe they feel in a particular environment, around others." [47]
**Education and Learning**	
Learning[a]	"The development of knowledge, skills, values, morals, beliefs, and habits."[2]
Education[b]	"The process of learning[a] and teaching, which refers to the process of facilitation of learning."[2]
Outdoor learning	"The learning[a] that takes place outdoors."[2]
Outdoor education	"The process of learning[a] and the facilitation of learning, that takes place outdoors."[2]
Land-based learning	An approach to education[b] that recognizes a deep connection and relationship of reciprocity between people and the land [2].
Place-based learning	"Learning[a] that considers the importance of connecting learners with their community by anchoring pedagogy within the context of the locally natural, cultural, and social ecosystems."[2]
**Movement Behaviors**	
Physical activity	"Any body movement produced by skeletal muscles resulting in a substantial increase over resting energy expenditure." [48]
Exercise	"Planned, structured, and repetitive bodily movement done to improve or maintain one or more components of physical fitness." [48]

Sport	"Sport participation is a subset of physical activity that is structured and goal-oriented; it can be competitive and/or contest-based."[49]
Sedentary behavior	“Any waking activity characterized by an energy expenditure ≤1.5 metabolic equivalents and a sitting or reclining posture” [50]
Sedentary screen time	"Time spent passively watching screen-based entertainment (e.g., TV, computer, mobile devices). Does not include active screen-based games where physical activity or movement is required." [51]

Note: While not all of the terms and definitions included in this table were used directly in the manuscript, they are provided to offer additional context and support understanding of the key concepts introduced.

Supplementary Table 2. List of reviews (N = 18) conducted to inform the *2025 Position Statement*.

**Review Title**	**Lead Author**	**Conceptual Framework Theme(s)**	**Protocol Registration/Publication***
**12 Reviews**			
Systematic review of the association between outdoor play and the 24-hour movement behaviours among children, youth and adults	Maeghan James	Movement Behaviors	https://www.crd.york.ac.uk/PROSPERO/view/CRD42024517145
Associations between active outdoor play and health and wellbeing among children, adolescents, and adults: an umbrella review	Louise de Lannoy	Health and Well-being	https://www.crd.york.ac.uk/PROSPERO/view/CRD42024565295
What is the relationship between outdoor risky play and health in children? Results from a systematic review	Mariana Brussoni	Health and Well-being	https://www.crd.york.ac.uk/PROSPERO/view/CRD42023488023
A mixed-methods systematic review of the association between active outdoor play and environmental stewardship outcomes among children, youth, and adults	Louise de Lannoy	Nature and Environment	https://www.crd.york.ac.uk/PROSPERO/view/CRD42024552064
Climate change and active outdoor play: a systematic review and qualitative synthesis	Eun-Young Lee	Nature and Environment	https://www.crd.york.ac.uk/PROSPERO/view/CRD42024560103
The PLAY+ (Play, Land, Animals, You and +) Framework: the role of active outdoor play in advancing One Health	Eun-Young Lee	One Health / Community, Connection, & Partnerships	https://www.outdoorplaycanada.ca/aop10-full-length-research-manuscripts-and-reports/
Active play among young children (0-4 years) with disabilities: a scoping review	Alessia Capone	Human Rights and Policy / Community, Connection, & Partnerships	https://doi.org/10.17605/OSF.IO/GWPSD
Places and spaces for play among children and youth with disabilities: an umbrella review	Leanne Abungin	Human Rights and Policy / Community, Connection, & Partnerships	https://doi.org/10.17605/OSF.IO/ZJ6US

'The state of play in outdoor play' - Exploring global Indigenous knowledge of outdoor play: a scoping review	River McRae	Human Rights and Policy / Community, Connection, & Partnerships	https://www.outdoorplaycanada.ca/aop10-full-length-research-manuscripts-and-reports/
Systematic review and qualitative meta-synthesis on (active) outdoor play and social capital: relationships and impacts	Seiyeong Park	Social Capital / Community, Connection, & Partnerships	https://www.crd.york.ac.uk/PROSPERO/view/CRD42024568624
Teacher implementation of active outdoor play-based learning: a systematic review of pedagogical models and practices	Steph Dean	Education and Learning	https://www.crd.york.ac.uk/PROSPERO/view/CRD42024551581
An environmental scan of global outdoor play-based projects, programs, and initiatives	Scott Duncan	Community, Connection, & Partnerships	https://www.outdoorplaycanada.ca/aop10-full-length-research-manuscripts-and-reports/
**World Region Reviews (6 reviews)**			
Outdoor play in the Africa: a status update	Dina Adjei Boadi	Human Rights and Policy / Community, Connection, & Partnerships	https://www.outdoorplaycanada.ca/aop10-full-length-research-manuscripts-and-reports/
Outdoor play in the Asia region: a status update	Eun-Young Lee	Human Rights and Policy / Community, Connection, & Partnerships	https://www.outdoorplaycanada.ca/aop10-full-length-research-manuscripts-and-reports/
Outdoor play in Europe: terminology and state of research, practice, and policy	Lærke Mygind	Human Rights and Policy / Community, Connection, & Partnerships	https://www.outdoorplaycanada.ca/aop10-full-length-research-manuscripts-and-reports/
Outdoor play in the Latin America and Caribbean region: a narrative review on the challenges, lessons learned, and recommendations for the future	Diego Augusto Santos Silva	Human Rights and Policy / Community, Connection, & Partnerships	https://www.outdoorplaycanada.ca/aop10-full-length-research-manuscripts-and-reports/

Outdoor play in the Northern America region: a status update	Mark S. Tremblay	Human Rights and Policy / Community, Connection, & Partnerships	https://www.outdoorplaycanada.ca/aop10-full-length-research-manuscripts-and-reports/
Outdoor play in Oceania: a review of policy, advocacy, and regional priorities	Scott Duncan	Human Rights and Policy / Community, Connection, & Partnerships	https://www.outdoorplaycanada.ca/aop10-full-length-research-manuscripts-and-reports/
* Some of these articles are currently in preparation, under journal review, or in press. For updates, please visit: https://www.outdoorplaycanada.ca/aop10-full-length-research-manuscripts-and-reports/.			

Supplementary Table 3. List of individuals who contributed to the translation of the Global Collaboration Consultation Survey and final 2025 Position Statement.

Language	Name	Affiliation
Arabic	Lina Majed	Hamad Bin Khalifa University
	Bayan Kaid	Outdoor Play Canada
	Ameneh Baghestani	MohaMohammed Bin Rashid University
Chinese	Wendy Huang	Hong Kong Baptist University
	Yang Liu	Shanghai University of Sport
	Sitong Chen	Victoria University, Australia
French	Richard Larouche	University of Lethbridge
	Manon Laviolette	Government of New Brunswick
	Salome Aubert	Active Healthy Kids Global Alliance
Russian	Iryna Demchenko	Carleton University
	Svitlana Demchenko	University of Ottawa
Spanish	Javier Brazo-Sayavera	Pablo de Olavide University
	Silvia Gonzalez	Bogota, Colombia
	Julissa Ortiz Brunel	Universidad de Guadalajara

Note: Initial translations were generated using ChatGPT, then reviewed and refined by native-speaking collaborators. All reviewer-provided translations were consolidated, and consensus was reached.
Translated versions of the Global Collaboration Consultation Survey are available in Appendix B.
Translated versions of the 2025 Position Statement on Active Outdoor Play in all six United Nations official languages are available in Appendix D.

References

1. de Lannoy L, Lee E-Y, Lopes O, Boadi DA, de Barros MIA, Duncan S, et al. 2025 Position Statement on Active Outdoor Play–Process and Methodology. Int J Behav Nutr Phys Act. https://doi.org/10.0.4.162/s12966-025-01806-8.
2. Lee E-Y, de Lannoy L, Li L, de Barros MIA, Bentsen P, Brussoni M, et al. Play, Learn, and Teach Outdoors-Network (PLaTO-Net): terminology, taxonomy, and ontology. Int J Behav Nutr Phys Act. 2022;19(1):66. doi:10.1186/s12966-022-01294-0
3. Veal AJ. Definitions of leisure and recreation. Australian Journal of Leisure and Recreation. 1992;2(4):44-8.
4. World Health Organization. Mental health. n.d. https://www.who.int/data/gho/data/themes/theme-details/GHO/mental-health#:~:text=%C3%97%20Subscribe%20here%20to%20receive,protect%20and%20restore%20mental%20health.
5. Doyle DM, Link BG. On social health: History, conceptualization, and population patterning. Health Psychol Rev. 2024;18(3):619-48.
6. McSherry W, Cash K. The language of spirituality: an emerging taxonomy. Int J Nurs Stud. 2004;41(2):151-61.
7. Hawks S. Spiritual health: definition and theory. Wellness Perspectives. 1994;10(4).
8. Miles RM, Chow MI, Tomasky G, Bredin SS, Kaufman KL, Warburton DE. Wholistic versus holistic: words matter for indigenous peoples. Health fit J Can. 2023;16(3):3-7.
9. Merriam-Wbster. Harm. n.d. www.merriam-webster.com/dictionary/harm. Accessed April 04 2025.
10. Tremblay MS, Gray C, Babcock S, Barnes J, Bradstreet CC, Carr D, et al. Position Statement on Active Outdoor Play. Int J Environ Res Public Health. 2015;12(6):6475-505. doi:10.3390/ijerph120606475
11. Brussoni M, Olsen LL, Pike I, Sleet DA. Risky play and children's safety: balancing priorities for optimal child development. Int J Environ Res Public Health. 2012;9(9):3134-48.
12. Wallach F. Playground safety: what did we do wrong? Parks and Recreation. 1992;27(4):52.
13. Scheer D, Benighaus C, Benighaus L, Renn O, Gold S, Röder B, et al. The distinction between risk and hazard: understanding and use in stakeholder communication. Risk Anal. 2014;34:1270-85. doi:10.1111/risa.12169
14. McArdle K, T. H, and Harrison D. Does a nurturing approach that uses an outdoor play environment build resilience in children from a challenging background? J Adventure Educ Outdoor Learn. 2013;13(3):238-54. doi:10.1080/14729679.2013.776862
15. Sisto A, Vicinanza F, Campanozzi LL, Ricci G, Tartaglini D, Tambone V. Towards a transversal definition of psychological resilience: a literature review. Medicina. 2019;55(11). doi:10.3390/medicina55110745
16. Lerner H, Berg C. A comparison of three holistic approaches to health: One Health, EcoHealth, and Planetary Health. Front Vet Sci. 2017;4:163. doi:10.3389/fvets.2017.00163
17. Keune H, Flandroy L, Thys S, De Regge N, Mori M, T. vdB, et al. European OneHealth/EcoHealth Workshop Report. Brussels: Belgian Community of Practice Biodiversity and Health, Belgian Biodiversity Platform2017.
18. Whitmee S, Haines A, Beyrer C, Boltz F, Capon AG, de Souza Dias BF, et al. Safeguarding human health in the Anthropocene epoch: report of The Rockefeller Foundation–Lancet Commission on planetary health. The Lancet. 2015;386(10007):1973-2028.
19. Lerner H, Berg C. The concept of health in One Health and some practical implications for research and education: what is One Health? Infect Ecol Epidemiol. 2015;5(1):25300.

20. Frumkin H. Environmental health: from global to local. Frumkin H, editor. San Francisco, CA: John Wiley & Sons; 2016.
21. Koplan JP, Bond TC, Merson MH, Reddy KS, Rodriguez MH, Sewankambo NK, et al. Towards a common definition of global health. The Lancet. 2009;373(9679):1993-5.
22. August E, Tadesse L, O'Neill MS, Eisenberg JN, Wong R, Kolars JC, et al. What is global health equity? A proposed definition. Ann Glob Health. 2022;88(1):50.
23. Dictionary L. Nature. n.d. https://www.ldoceonline.com/dictionary/nature. Accessed April 04 2025.
24. Intergovernmental Panel on Climate Change. Synthesis Report of the IPCC Sixth Assessment Report - Longer Report: Intergovernmental Panel on Climate Change2023.
25. Solutions CfCaE. What is climate resilience, and why does it matter? In: Climate Essentials. Center for Climate and Energy Solutions. 2019. https://www.c2es.org/document/what-is-climate-resilience-and-why-does-it-matter/.
26. Shaw C, Hales S, Howden-Chapman P, Edwards R. Health co-benefits of climate change mitigation policies in the transport sector. Nat Clim Chang. 2014;4(6):427-33.
27. Lange F, Dewitte S. Measuring pro-environmental behavior: Review and recommendations. J Environ Psychol. 2019;63:92-100.
28. Bennett NJ, Whitty TS, Finkbeiner E, Pittman J, Bassett H, Gelcich S, et al. Environmental stewardship: A conceptual review and analytical framework. Environ Manage. 2018;61:597-614.
29. Schultz PW. Inclusion with nature: The psychology of human-nature relations. In: Schmuck P, Schultz PW, editors. Psychology of Sustainable Development. New York, NY.: Springer; 2002. p. 61-78.
30. Vos RO. Defining sustainability: a conceptual orientation. J Chem Technol Biotechnol. 2007;82(4):334-9.
31. Moore JE, Mascarenhas A, Bain J, Straus SE. Developing a comprehensive definition of sustainability. Implement Sci. 2017;12:1-8.
32. Kelly C, Dansereau L, Sebring J, Aubrecht K, FitzGerald M, Lee Y, et al. Intersectionality, health equity, and EDI: What's the difference for health researchers? Int J Equity Health. 2022;21(1):182.
33. Persson H, Åhman H, Yngling AA, Gulliksen J. Universal design, inclusive design, accessible design, design for all: different concepts—one goal? On the concept of accessibility—historical, methodological and philosophical aspects. Universal Access in the Information Society. 2015;14:505-26.
34. Kuokkanen R. Reshaping the university: Responsibility, Indigenous epistemes, and the logic of the gift. Vancouver: University of British Columbia Press; 2011.
35. Truth Reconciliation Commission of Canada. Final Report of the Truth and Reconciliation Commission of Canada, Volume One: Summary: Honouring the Truth, Reconciling for the Future. Toronto: James Lorimer & Company; 2015.
36. Mackey E. Unsettled expectations: Uncertainty, land and settler decolonization. Halifax: Fernwood Publishing; 2016.
37. Moreton-Robinson A. The white possessive: Property, power, and indigenous sovereignty. Minneapolis: University of Minnesota Press; 2015.
38. United Nations Economic and Social Commission of Western Asia. Indigenization. n.d. https://archive.unescwa.org/indigenization. Accessed April 8 2025.
39. Tynan L. What is relationality? Indigenous knowledges, practices and responsibilities with kin. Cult Geogr. 2021;28(4):597-610.
40. Mbah MF, Bailey M, Shingruf A. Considerations for relational research methods for use in Indigenous contexts: Implications for sustainable development. Int J Soc Res Methodol. 2024;27(4):431-46.

41. Putnam RD. Bowling alone: The collapse and revival of American community. New York, NY: Simon & Schuster Paperbacks; 2000.
42. Ehsan A, Klaas HS, Bastianen A, Spini D. Social capital and health: a systematic review of systematic reviews. SSM Popul Health. 2019;8:100425.
43. Gottlieb BH, Bergen AE. Social support concepts and measures. J Psychosom Res. 2010;69(5):511-20.
44. McMillan DW, Chavis DM. Sense of community: A definition and theory. J Community Psychol. 1986;14(1):6-23.
45. Baumeister RF, Leary MR. The need to belong: Desire for interpersonal attachments as a fundamental human motivation. In: Baumeister R, F., Leary M, R., editors. Interpersonal Development. London: Routhledge; 2017. p. 57-89.
46. Holt-Lunstad J, Robles TF, Sbarra DA. Advancing social connection as a public health priority in the United States. Am Psychol. 2017;72(6):517.
47. Syropoulos S, Leidner B, Mercado E, Li M, Cros S, Gomez A, et al. How safe are we? Introducing the multidimensional model of perceived personal safety. Pers Individ Dif. 2024;224:112640.
48. Caspersen CJ, Powell KE, Christenson GM. Physical activity, exercise, and physical fitness: Definitions and distinctions for health-related research. Public Health Rep. 1985;100(2):126.
49. ParticipACTION. ParticipACTION Report Card on Physical Activity for Adults. In: Moving toward a better normal: . ParticipACTION, Toronto. 2021. https://www.participaction.com/wp-content/uploads/2022/09/2021-ParticipACTION-Report-Card-on-Physical-Activity-for-Adults.pdf?x12631. Accessed January 12 2025.
50. Tremblay MS, Aubert S, Barnes JD, Saunders TJ, Carson V, Latimer-Cheung AE, et al. Sedentary Behavior Research Network (SBRN) - Terminology Consensus Project process and outcome. Int J Behav Nutr Phys Act. 2017;14(1):75. doi:10.1186/s12966-017-0525-8
51. World Health Organization. Guidelines on physical activity, sedentary behaviour and sleep for children under 5 years of age. Geneva: World Health Organization2019.
