# Supplementary material for: 2025 Position statement on active outdoor play
Source: Int J Behav Nutr Phys Act. 2025 Sep 25;22:117. doi: 10.1186/s12966-025-01813-9 (PMC12462132; doi:10.1186/s12966-025-01813-9)
Supplement: Supplementary file 2 — Appendix B [file 12966_2025_1813_MOESM2_ESM.pdf]

Survey on the 10-Year Update to the Position Statement on Active Outdoor Play

Survey Round 1

You are being invited to participate in a survey soliciting your opinion on the draft 10-year update to the Position Statement on Active Outdoor Play, prepared by an 11-person global outdoor play leadership group, with representatives from every continent, including: Dina Adjei Boadi (Ghana), Isabel de Barros (Brazil), Scott Duncan (New Zealand), Laerke Groenfeldt (Denmark), Maeghan James (Canada), Robyn Monro Miller (Australia), Leigh Vanderloo (Canada), Po-Yu Wang (Taiwan), Eun-Young Lee (South Korea/Canada), Louise de Lannoy (Canada), Mark Tremblay (Canada).

You are being invited to participate because you have signed up to be part of the 100+ international Steering Committee for this project, to help ensure this project achieves global reach, representation, and meaning.

As you may recall, the 2015 Position Statement on Active Outdoor Play and the systematic reviews that informed it highlighted the unequivocal benefits of outdoor play for child growth and development - mentally, emotionally, cognitively, and socially. The 2015 Position Statement informed both Canadian and international policy documents, a Canadian Supreme Court decision against a playground injury lawsuit, substantial philanthropic investment, and a surge in Canadian and international-led outdoor play-focused research projects.

In 2025, it will be 10 years since the release of the 2015 Position Statement and to celebrate, Outdoor Play Canada has assembled the above-mentioned international Leadership Group to spearhead, and international Steering Committee to guide and provide feedback on, an update to the Position Statement on Active Outdoor Play (hereafter called the 'AOP10' project). Given the number of new projects and initiatives that have come about since that time, this project is an opportunity to describe its impact, consolidate similar position statements, and broaden the net of outdoor play stakeholders. Accordingly, this update has expanded to include all ages (i.e., children, youth, adults, and older adults), all countries, and is centered around the following conceptual framework (Figure 1):

Figure 1. Conceptual Framework for the 10-year Anniversary Update of the Position Statement on Outdoor Play Project. The outer green-blue circle contains central themes related to outdoor play, where all themes have overlapping relationships. In the middle, surrounding the purple 'Outdoor Play' circle, are light and dark purple concentric overlapping arrows indicating benefits and risks, to indicate that all themes have benefits and risks associated with outdoor play, where risks can beget benefits and vice versa.

Using this framework as a guide, this collective team developed 10 systematic reviews and 6 continental narrative reviews to inform the updated Position Statement on Active Outdoor Play. As an initial stage in the development of this Position Statement we are seeking to gain feedback from you about the phrasing and clarity, as well as level of agreement, perceived importance, and support for the Position Statement and supporting evidence statements.

This stakeholder survey will be open until January 21st, 2025. The survey should take you no longer than 10 minutes to complete. On the next series of screens you will be presented with each proposed section of the Position Statement. You may advance or go back using the buttons on the bottom of the screen. Participation in this survey is voluntary. By accessing and completing this survey you are giving your implied/passive consent to participate in the survey. The survey does not collect information about your name or email address and responses will be presented in group format only. A summary of findings will be available on the OPC website (www.outdoorplaycanada.ca) once the results have been published and will also be sent to participating Steering Committee members for broader distribution. If you have any questions about this study, please contact Dr. Louise de Lannoy at ldelannoy@outdoorplaycanada.ca.

Note: This Position Statement on Active Outdoor Play is in draft form and not intended for general circulation. This is a first rough draft - you will see further iterations for comment as it develops.

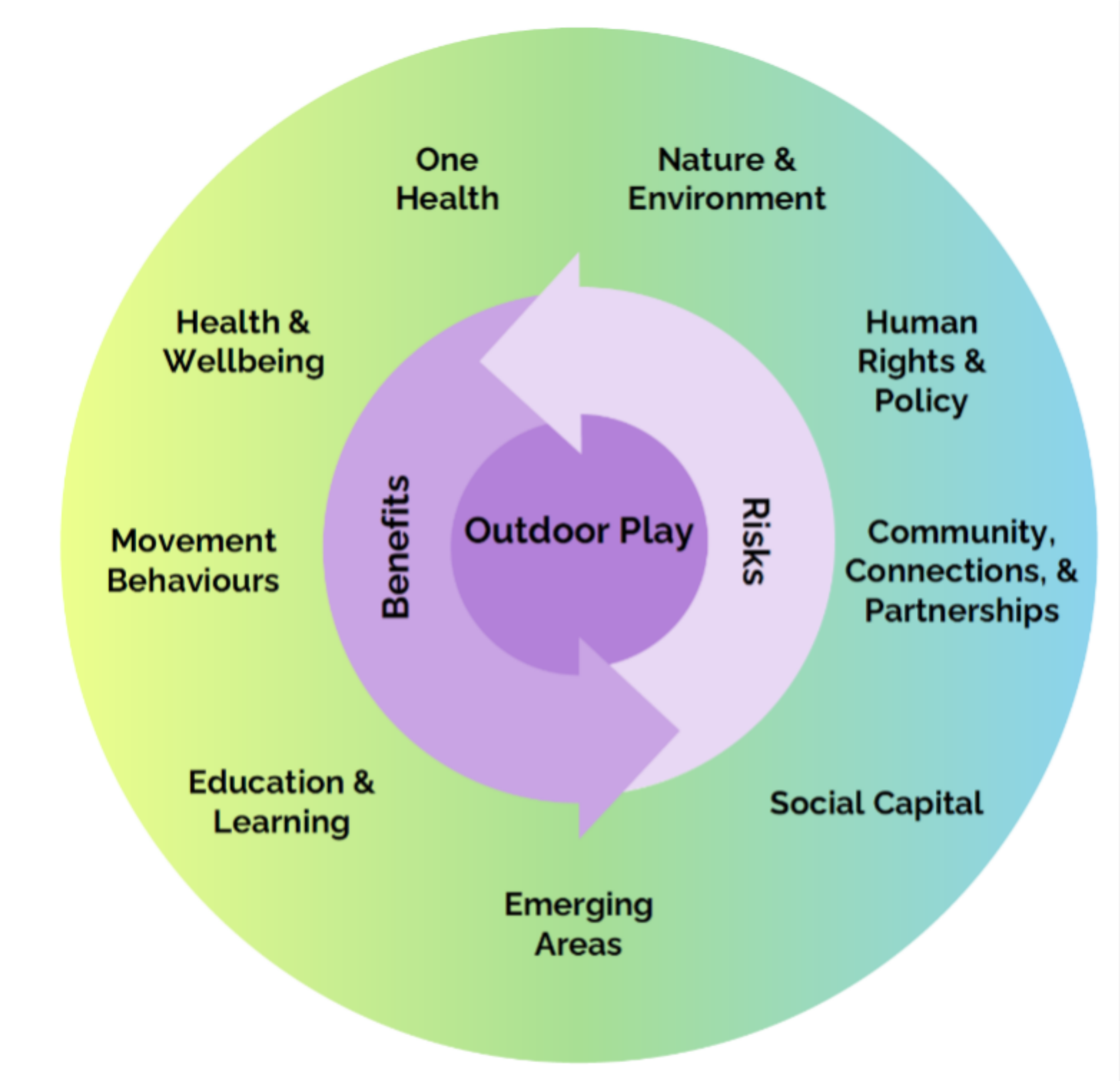
One Health
Nature & Environment
Health & Wellbeing
Human Rights & Policy
Movement Behaviours
Benefits
Outdoor Play
Risks
Community, Connections, & Partnerships
Education & Learning
Social Capital
Emerging Areas

SECTION 1 - Position Statement
The following Position Statement serves as an update to the 2015 Position Statement on Active Outdoor Play, building on that previous statement and new evidence gathered from 10 systematic reviews and 6 continental reviews.

The 2015 Position Statement stated the following: "Access to active play in nature and outdoors-with its risks-is essential for healthy child development. We recommend increasing children's opportunities for self-directed play outdoors in all settings-at home, at school, in childcare, the community and nature."

The proposed 2025 updated Position Statement on Active Outdoor Play is as follows:

"Active outdoor play - with its risks - is beneficial for the health and wellbeing of people, communities, and the planet. We need to work together across sectors and settings to preserve, promote, and provide equitable access to active play outdoors and in nature."

	Strongly agree	Somewhat agree	Neither agree nor disagree	Somewhat disagree	Strongly disagree
Do you consider the main Position Statement to be clearly stated?	○	○	○	○	○
Do you agree with the Position Statement?	○	○	○	○	○

Do you have any suggested edits to/comments on the Position Statement? ______________________________

SECTION 2 - Evidence

A. The evidence remains that when people are outside, they move more, sit less and play longer. Engaging in active outdoor play is linked to less screen time and better sleep.

• Engaging in more active outdoor play is related to increased physical activity and decreased sedentary behaviour and screen time in children and youth aged 0 to 15 years old.
• Engaging in active outdoor play may or may not positively impact sleep in children and youth aged 0 to 15 years.
• Take home message: Engaging in more active outdoor play is associated with increased physical activity levels and less time spent in sedentary behaviour and on screens. AOP may also result in better and/or longer duration of sleep.

	Strongly agree	Somewhat agree	Neither agree nor disagree	Somewhat disagree	Strongly disagree
Do you consider this evidence to be clearly stated?	○	○	○	○	○
Do you agree with the way this evidence was stated?	○	○	○	○	○
Does this evidence align with your understanding, research, and/or practice?	○	○	○	○	○

Do you have additional suggestions regarding this evidence? ______________________

SECTION 2 - Evidence
B. Active outdoor play is favourably associated with physical, mental, social and spiritual health across all ages.

• There is a significant positive association between active outdoor play and mental, physical, spiritual, and social health, with particular emphasis on the associations between active outdoor play and positive mental health (e.g., resilience, positive affect).
• Favourable associations between indicators of spiritual health and wellbeing and active outdoor play were also evident.

	Strongly agree	Somewhat agree	Neither agree nor disagree	Somewhat disagree	Strongly disagree
Do you consider this evidence to be clearly stated?	○	○	○	○	○
Do you agree with the way this evidence was stated?	○	○	○	○	○
Does this evidence align with your understanding, research, and/or practice?	○	○	○	○	○

Do you have additional suggestions regarding this evidence? ______________________________

SECTION 2 - Evidence
C. The outdoors creates opportunities for risky play, which is linked to positive health outcomes.

• Risky play have positive associations with children's health.
• Independent mobility is convincingly associated with more physical activity but may not always be good for wellbeing.

	Strongly agree	Somewhat agree	Neither agree nor disagree	Somewhat disagree	Strongly disagree
Do you consider this evidence to be clearly stated?	○	○	○	○	○
Do you agree with the way this evidence was stated?	○	○	○	○	○
Does this evidence align with your understanding, research, and/or practice?	○	○	○	○	○

Do you have additional suggestions regarding this evidence? __________________________________

SECTION 2 - Evidence

D. Active outdoor play has the potential to bridge gaps between different health paradigms (e.g., One health, global health, planetary health).

- **Active outdoor play may serve as a conduit to promote integrated health strategies, bridging gaps between different health paradigms.**
- **Active outdoor play aligns with public health goals, environmental sustainability, and equitable access to recreational opportunities.**
- **Active outdoor play has multifaceted benefits and policy relevance.**
- **It is important to embed active outdoor play in health initiatives, with a focus on interdisciplinary approaches and systemic support.**

	Strongly agree	Somewhat agree	Neither agree nor disagree	Somewhat disagree	Strongly disagree
Do you consider this evidence to be clearly stated?	◯	◯	◯	◯	◯
Do you agree with the way this evidence was stated?	◯	◯	◯	◯	◯
Does this evidence align with your understanding, research, and/or practice?	◯	◯	◯	◯	◯

Do you have additional suggestions regarding this evidence? ________________________________

SECTION 2 - Evidence
E. Active outdoor play can foster a connection to nature and environmental stewardship. While climate change poses a challenge to active outdoor play, active outdoor play can serve as an adaptive strategy to foster climate resilience.

• There are many explanatory/influencing variables for why active outdoor play may foster a connection to nature (e.g., nature recreation leads to personal transformation which in turn leads to a stronger connection to nature) and the consequences of that connection (e.g., viewing nature as part of a larger ecological web fosters an interconnected worldview).
• Air quality alerts and public perception, rather than actual pollution levels, appear to influence individuals' decisions to engage in active outdoor play; improved communication and public understanding can help ensure safer and more informed participation.
• Compared to alternatives such as passive outdoor or active indoor activities, the impact of climate change on active outdoor play may not be as detrimental as commonly perceived, highlighting opportunities to implement adaptive strategies that promote active outdoor play.
• It is important to account for potential environmental impacts of active outdoor play. Research indicates that transportation to and from outdoor spaces and energy use associated with long-distance travel and accommodations have the most detrimental effects on the environment. A key recommendation may be to promote accessible local opportunities for active outdoor play to minimize these impacts.

	Strongly agree	Somewhat agree	Neither agree nor disagree	Somewhat disagree	Strongly disagree
Do you consider this evidence to be clearly stated?	○	○	○	○	○
Do you agree with the way this evidence was stated?	○	○	○	○	○
Does this evidence align with your understanding, research, and/or practice?	○	○	○	○	○

Do you have additional suggestions regarding this evidence? ______________________

SECTION 2 - Evidence
F. Active outdoor play and community belongingness, culture, and social connection share a mutually beneficial relationship.

• Social capital and active outdoor play have bidirectional relationships. Specifically, active outdoor play contributes to building positive social capital, while social capital factors can create supportive environments for greater engagement in active outdoor play.
• Research supports the importance of social support and safety across all age groups.
• Multiple social capital factors (e.g., social cohesion, social connectedness, perceived safety) can simultaneously influence active outdoor play, emphasizing the interconnected nature of these relationships.

	Strongly agree	Somewhat agree	Neither agree nor disagree	Somewhat disagree	Strongly disagree
Do you consider this evidence to be clearly stated?	○	○	○	○	○
Do you agree with the way this evidence was stated?	○	○	○	○	○
Does this evidence align with your understanding, research, and/or practice?	○	○	○	○	○

Do you have additional suggestions regarding this evidence? ______________________________

SECTION 2 - Evidence
G. Active outdoor play can facilitate diverse learning opportunities

• Some literature indicates that knowledge retention may be similar in outdoor and indoor settings. Implementing playful pedagogies outdoors may therefore expand the places where learning happens and help meet the needs of a wider diversity of learners.
• Active outdoor play-based learning (AOPL) is achieved through diverse activities, such as gardening, game-based learning, and inquiry-driven projects.
• Teachers play pivotal roles as facilitators, leaders, or co-learners, tailoring activities to blend play and academic goals.
• Effective AOPL depends on educators' ability to integrate play with explicit curricular goals.

	Strongly agree	Somewhat agree	Neither agree nor disagree	Somewhat disagree	Strongly disagree
Do you consider this evidence to be clearly stated?	○	○	○	○	○
Do you agree with the way this evidence was stated?	○	○	○	○	○
Does this evidence align with your understanding, research, and/or practice?	○	○	○	○	○

Do you have additional suggestions regarding this evidence? ________________________________

SECTION 3 - Considerations and Future Directions

A. Cultural and Global Considerations

In developing the updated Position Statement, the AOP10 Leadership Group discussed at length the importance of contextual sensitivity; the need to consider regions with differing priorities or cultural contexts (i.e., in countries where outdoor play is embedded into culture and way of life, there may not be a need for active outdoor play policies), global diversity in language, and cultural perceptions of outdoor play, which can each impact how this statement is read, interpreted, and used (or not used).

Do you agree with these Cultural and Global Considerations within the Position Statement?

- ◯ Strongly agree
- ◯ Somewhat agree
- ◯ Neither agree nor disagree
- ◯ Somewhat disagree
- ◯ Strongly disagree

Do you have additional suggestions regarding these Cultural and Global Considerations?

____

B. Emerging Areas

From the above evidence discussed, knowledge gaps and emerging areas for future research, policy and practice were identified. These include:

• Associations between 24-hour movement behaviours and active outdoor play in adult populations.
• Cognitive/academic outcomes of risky outdoor play.
• Risky outdoor play outside of the global north.
• Conceptual and empirical studies to bridge the gap between practice and research.
• Designing (or redesigning) learning environments to incorporate active outdoor play and learning, and evaluating the effectiveness of integrating active outdoor play into formal education systems.
• Built environments (e.g., open space, accessible space, shaded areas) conducive to active outdoor play.
• Links between active outdoor play and the UN Sustainable Development Goals, with potential to address global challenges such as climate change and principles of justice, equity, diversity, and inclusion, across sectors.
• Potential of active outdoor play in mitigating the negative impact of social media use (e.g., on mental, physical, social health and wellbeing)

Do you agree with these Emerging Areas?

- ◯ Strongly agree
- ◯ Somewhat agree
- ◯ Neither agree nor disagree
- ◯ Somewhat disagree
- ◯ Strongly disagree

Do you have additional suggestions regarding these or other Emerging Areas?

____

SECTION 4 - Other Feedback

Do you feel this update to the Position Statement on Active Outdoor Play is important to public health?	◯ Yes<br>◯ No<br>◯ Unsure
Do you feel this update to the Position Statement on Active Outdoor Play is important to global and planetary health?	◯ Yes<br>◯ No<br>◯ Unsure
Do you feel this update to the Position Statement on Active Outdoor Play is important for meeting the United Nations Sustainable Development Goals?	◯ Yes<br>◯ No<br>◯ Unsure
Do you feel this update to the Position Statement on Active Outdoor Play is important to you and/or your job?	◯ Yes<br>◯ No<br>◯ Unsure
Do you think the Position Statement is relevant/applicable to the country you reside in?	◯ Yes<br>◯ No<br>◯ Unsure
Please enter any additional comments you would like to add regarding this update to the Position Statement on Active Outdoor Play:	__________________________________

SECTION 5 - Demographic Information

What sector do you represent/work in?

- ☐ Early childhood education
- ☐ Elementary to secondary school
- ☐ Health professional
- ☐ Allied health professional (e.g., public health professional, physiotherapist, nurse, dietitian, occupational therapist)
- ☐ Fitness professional (e.g., kinesiologist)
- ☐ Injury prevention
- ☐ Insurance
- ☐ Law
- ☐ Professor/researcher
- ☐ Non-governmental organization staff or board member
- ☐ Government
- ☐ Other

Please specify what sector you represent/work in.

____

In what country do you primarily live, work, and play?

◯ Afghanistan
◯ Albania
◯ Algeria
◯ Andorra
◯ Angola
◯ Antigua and Barbuda
◯ Argentina
◯ Armenia
◯ Australia
◯ Austria
◯ Azerbaijan
◯ Bahamas
◯ Bahrain
◯ Bangladesh
◯ Barbados
◯ Belarus
◯ Belgium
◯ Belize
◯ Benin
◯ Bhutan
◯ Bolivia
◯ Bosnia and Herzegovina
◯ Botswana
◯ Brazil
◯ Brunei
◯ Bulgaria
◯ Burkina Faso
◯ Burundi
◯ Cabo Verde
◯ Cambodia
◯ Cameroon
◯ Canada
◯ Central African Republic
◯ Chad
◯ Chile
◯ China
◯ Colombia
◯ Comoros
◯ Congo (Congo-Brazzaville)
◯ Costa Rica
◯ Croatia
◯ Cuba
◯ Cyprus
◯ Czech Republic (Czechia)
◯ DR Congo (Congo-Kinshasa)
◯ Denmark
◯ Djibouti
◯ Dominica
◯ Dominican Republic
◯ Ecuador
◯ Egypt
◯ El Salvador
◯ Equatorial Guinea
◯ Eritrea
◯ Estonia
◯ Eswatini
◯ Ethiopia
◯ Fiji
◯ Finland
◯ France
◯ Gabon
◯ Gambia
◯ Georgia
◯ Germany
◯ Ghana
◯ Greece
◯ Grenada
◯ Guatemala
◯ Guinea

◯ Guinea-Bissau
◯ Guyana
◯ Haiti
◯ Honduras
◯ Hungary
◯ Iceland
◯ India
◯ Indonesia
◯ Iran
◯ Iraq
◯ Ireland
◯ Israel
◯ Italy
◯ Jamaica
◯ Japan
◯ Jordan
◯ Kazakhstan
◯ Kenya
◯ Kiribati
◯ Korea, North
◯ Korea, South
◯ Kuwait
◯ Kyrgyzstan
◯ Laos
◯ Latvia
◯ Lebanon
◯ Lesotho
◯ Liberia
◯ Libya
◯ Liechtenstein
◯ Lithuania
◯ Luxembourg
◯ Madagascar
◯ Malawi
◯ Malaysia
◯ Maldives
◯ Mali
◯ Malta
◯ Marshall Islands
◯ Mauritania
◯ Mauritius
◯ Mexico
◯ Micronesia
◯ Moldova
◯ Monaco
◯ Mongolia
◯ Montenegro
◯ Morocco
◯ Mozambique
◯ Myanmar
◯ Namibia
◯ Nauru
◯ Nepal
◯ Netherlands
◯ New Zealand
◯ Nicaragua
◯ Niger
◯ Nigeria
◯ North Macedonia
◯ Norway
◯ Oman
◯ Pakistan
◯ Palau
◯ Panama
◯ Papua New Guinea
◯ Paraguay
◯ Peru
◯ Philippines
◯ Poland
◯ Portugal
◯ Qatar

◯ Romania
◯ Russia
◯ Rwanda
◯ Saint Kitts and Nevis
◯ Saint Lucia
◯ Saint Vincent and the Grenadines
◯ Samoa
◯ San Marino
◯ Sao Tome and Principe
◯ Saudi Arabia
◯ Senegal
◯ Serbia
◯ Seychelles
◯ Sierra Leone
◯ Singapore
◯ Slovakia
◯ Slovenia
◯ Solomon Islands
◯ Somalia
◯ South Africa
◯ South Sudan
◯ Spain
◯ Sri Lanka
◯ Sudan
◯ Suriname
◯ Sweden
◯ Switzerland
◯ Syria
◯ Taiwan
◯ Tajikistan
◯ Tanzania
◯ Thailand
◯ Timor-Leste
◯ Togo
◯ Tonga
◯ Trinidad and Tobago
◯ Tunisia
◯ Turkey
◯ Turkmenistan
◯ Tuvalu
◯ Uganda
◯ Ukraine
◯ United Arab Emirates
◯ United Kingdom
◯ United States
◯ Uruguay
◯ Uzbekistan
◯ Vanuatu
◯ Vatican City
◯ Venezuela
◯ Vietnam
◯ Yemen
◯ Zambia
◯ Zimbabwe

AOP10 Consultation Survey Round 2

Background

Thank you for completing the first survey on the draft 10-year update to the Position Statement on Active Outdoor Play. In response to your insightful and thorough feedback, we have made substantial changes to the Position Statement and supporting evidence statements. Accordingly, we are seeking your additional review and feedback on these changes before we circulate to the broader outdoor play community.

Some of the major changes we made to the Position Statement based on your feedback, include:

Summarized the evidence statements into more digestible, short paragraphs, with citationsImproved the clarity, accuracy, and consistency of the language used across all statementsCreated a glossary of terms and definitions (available here and will be available as an appendix with the final Position Statement)For a fulsome review of all major comments and how we aimed to address them, download the following file: AOP10 Statement Suggestions Sheet.xlsx

For reference, you can view the 2015 Position Statement .pdf

Finally, we heard from you that there was a lack of clarity on who this statement applies to and how it differs from the 2015 Position Statement. Below is a table outlining the major differences between the two statements. This table will be available as an appendix within the final 2025 Position Statement.

What's Different?

2015 Statement

2025 Statement

1. Update and impact

first Position Statement

impact of 2015 Position Statement compiled and inclusion of a decade of newer, broader evidence

2. Age inclusive

focused solely on children

acknowledges that the benefits of active outdoor play extend across all ages

3. Global and cross-cultural perspectives

centred on Canadian contexts

takes a global perspective, acknowledging diverse cultural, environmental, and policy contexts

4. Commitment to Equity, Diversity, Inclusion, and Accessibility (EDIA)

did not explicitly address accessibility and equity

prioritizes ensuring that active outdoor play is accessible to all, with equity as the centrepiece

5. Beyond risky play

emphasized risky play as a critical component of child development

takes a broader approach, addressing active outdoor play within the context of multiple global challenges (e.g., climate change, urbanization, physical inactivity, health crises, epidemics/pandemics) and its role in fostering resilience

6. Climate change and sustainability integration

did not explicitly address environmental sustainability

highlights the role of outdoor play in fostering environmental stewardship, nature connection, and climate resilience

7. Emphasis on overall health and well-being

focused primarily on physical health benefits

emphasizes the health benefits of active outdoor play from a holistic perspective, encompassing physical, mental, social, emotional, and spiritual health

8. Expanded sectoral collaboration

primarily directed at educators, parents, and policymakers

guided by an expanded and more diverse conceptual framework facilitating broader multisectoral collaboration and collective advocacy

9. Focus on upstream interventions

largely addressed individual and community-level benefits of active outdoor play

embraced a systems approach, prioritizing upstream interventions (e.g., policies, infrastructure, governance) in addition to downstream solutions

10. Integration into global priorities

focused on physical activity and risk in the Canadian context

deliberate integration into broader global priorities including the United Nations Sustainable Development Goals and World Health Organization priorities

Thank you once again for your contribution to this important project! This consultation survey will be open until February 21st, 2025.

As a reminder, the survey should take you no longer than 10 minutes to complete. On the next series of screens, you will be presented with each proposed section of the Position Statement. You may advance or go back using the buttons on the bottom of the screen. Participation in this survey is voluntary. By accessing and completing this survey you are giving your implied/passive consent to participate in the survey. The survey does not collect information about your name or email address and responses will be presented in group format only. A summary of findings will be available on the OPC website (www.outdoorplaycanada.ca) once the results have been published, they will also be sent to participating Steering Committee members for broader distribution. If you have any questions about this study, please contact Dr. Eun-Young Lee at eunyoung.lee@queensu.ca.

Note: This Position Statement on Active Outdoor Play is in draft form and not intended for general circulation. This is a rough draft - you will see further iterations for comment as it develops.

Play-amble

The 2015 Position Statement on Active Outdoor Play 1 and its supporting evidence 2-3 highlighted the benefits of active outdoor play for children's overall health and wellbeing. A decade has passed since it was published, during which time we have seen a 10-fold increase in research publications 4, a surge in philanthropic funding for outdoor play initiatives 5, and use of the Position Statement to inform policy, research, and practice at a global level6. To celebrate these achievements while integrating current evidence and addressing global challenges, an international leadership group was convened to create the 2025 Position Statement on Active Outdoor Play. We conducted 10 systematic reviews, 6 continental narrative reviews, scanned the relevant grey literature, and consulted with experts around the world to synthesize the best available evidence on the relationship between active outdoor play and nine key themes (see link to the conceptual framework for this project at the bottom of the page).

The 2025 Position Statement on Active Outdoor Play envisions a world where active outdoor play is recognized as a powerful tool for addressing global challenges, from global health crises to the impacts of climate change, while advancing several United Nations Sustainable Development Goals 7. We are committed to fostering healthier, more resilient communities by embedding active outdoor play into daily life and ensuring all individuals - regardless of age, background, or ability - have equitable access and opportunities.

As a call to action, we provide recommendations for systemic change - along with recommendations for individual and community actors - advocating for policies and practices that position active outdoor play as a fundamental right and an essential component of sustainable, thriving societies at local, national, and global levels (see link to a full reference list at the bottom of the page)

	Strongly agree	Somewhat agree	Neither agree nor disagree	Somewhat disagree	Strongly disagree
1) Do you consider this section to be clearly stated?	○	○	○	○	○
2) Do you agree with the way this section was stated?	○	○	○	○	○
3) Does this section align with your understanding, research, and/or practice?	○	○	○	○	○

4) Do you have any suggested edits to/ comments on the pre-amble?

____

See the conceptual framework for this project here:

[Attachment: "Conceptual Framework_2024.pdf"]

See the full reference list here:

[Attachment: "AOP10_References.pdf"]

The proposed 2025 Position Statement is:

Active outdoor play is beneficial for the holistic health and well-being of people, communities, environments, and the planet. This is especially important given the multiple global challenges we face today. We recommend increasing opportunities for active outdoor play in all settings where people live, learn, work, and recreate. To achieve this, we must collaborate across sectors, settings, and societies to preserve and promote equitable access to active play outdoors and in nature.

		Strongly agree	Somewhat agree	Neither agree nor disagree	Somewhat disagree	Strongly disagree
5)	Do you consider this section to be clearly stated?	○	○	○	○	○
6)	Do you agree with the way this section was stated?	○	○	○	○	○
7)	Does this section align with your understanding, research, and/or practice?	○	○	○	○	○

8) Do you have any suggested edits to/ comments on the position statement? __________________________________

[Starting on this page, we present key information nuggets derived from the work conducted for each theme. These are meant to be catchy and informative. After each statement, the descriptor paragraph provides more context with references to highlight that the information is backed by evidence - additional reference suggestions are welcomed]

Move more, sit less, sleep better, and play longer

When people play outdoors, they engage in more physical activity and less sedentary behaviour and less screen time, leading to better sleep, and reduced stress 9-17. Outdoor environments foster a sense of playfulness, enticing individuals to engage in spontaneous, fun activities.

	Strongly agree	Somewhat agree	Neither agree nor disagree	Somewhat disagree	Strongly disagree
9) Do you consider this evidence to be clearly stated?	○	○	○	○	○
10) Do you agree with the way this evidence was stated?	○	○	○	○	○
11) Does this evidence align with your understanding, research, and/or practice?	○	○	○	○	○

12) Do you have any suggested edits to/ comments on this evidence section? ______________________________

A powerful catalyst for lifelong health and well-being

Active outdoor play fosters physical vitality, mental health, social connection, and a sense of community across all ages18-23, 24-26. Playing outdoors can offer an opportunity for people of all ages to connect with each other, nature, and within themselves in ways that are both healthy and enriching18,20,21.

	Strongly agree	Somewhat agree	Neither agree nor disagree	Somewhat disagree	Strongly disagree
13) Do you consider this evidence to be clearly stated?	○	○	○	○	○
14) Do you agree with the way this evidence was stated?	○	○	○	○	○
15) Does this evidence align with your understanding, research, and/or practice?	○	○	○	○	○

16) Do you have any suggested edits to/ comments on this evidence section? __________________________________

Facilitate diverse learning opportunities

When integrated into curricular goals, active outdoor play can unlock a world of rich, hands-on learning experiences that spark curiosity, creativity, and problem-solving27-30. It nurtures resilience and adaptability, supporting physical, social, and cognitive growth throughout the life-course31-34.

		Strongly agree	Somewhat agree	Neither agree nor disagree	Somewhat disagree	Strongly disagree
17)	Do you consider this evidence to be clearly stated?	○	○	○	○	○
18)	Do you agree with the way this evidence was stated?	○	○	○	○	○
19)	Does this evidence align with your understanding, research, and/or practice?	○	○	○	○	○

20) Do you have any suggested edits to/ comments on this evidence section? ______________________________

Promote confidence, naturally

The outdoors provides a dynamic playground for adventurous, risk-taking play-an essential ingredient for healthy development at any age. Whether it is testing your limits or taking risks within your comfort zone, engaging in play that is physically, mentally or emotionally challenging helps build confidence, resilience, and problem-solving skills, while promoting fitness, physical literacy, and mental toughness3, 24-26, 35-53.

	Strongly agree	Somewhat agree	Neither agree nor disagree	Somewhat disagree	Strongly disagree
21) Do you consider this evidence to be clearly stated?	○	○	○	○	○
22) Do you agree with the way this evidence was stated?	○	○	○	○	○
23) Does this evidence align with your understanding, research, and/or practice?	○	○	○	○	○

24) Do you have any suggested edits to/ comments on this evidence section? ______________________

A pathway to a happier, healthier planet

Active outdoor play can serve as a unifying force that bridges diverse health paradigms - One Health, global health, and planetary health - by recognizing the interconnectedness of human, environmental, and animal well-being54-55. It fosters healthier communities, stronger ecosystems, and a more sustainable planet1,56-59, making it a powerful ally for resilient communities and ecosystems in an ever-changing world while promoting interdisciplinary approaches involving public health, education and environmental sectors.

		Strongly agree	Somewhat agree	Neither agree nor disagree	Somewhat disagree	Strongly disagree
25)	Do you consider this evidence to be clearly stated?	○	○	○	○	○
26)	Do you agree with the way this evidence was stated?	○	○	○	○	○
27)	Does this evidence align with your understanding, research, and/or practice?	○	○	○	○	○

28) Do you have any suggested edits to/ comments on this evidence section? ______________________

Build resilience and stewardship for a sustainable future

Active outdoor play not only nurtures a connection to nature but also instills a lifelong sense of environmental stewardship60-63. Climate change may threaten opportunities for active outdoor play; but, embracing it as part of daily life can be a powerful adaptive strategy by building resilience and cultivating a proactive commitment to protecting the planet. By fostering an intergenerational culture that plays, learns, and thrives outdoors, we empower communities to adapt to climate challenges and champion a healthier, more sustainable future.

	Strongly agree	Somewhat agree	Neither agree nor disagree	Somewhat disagree	Strongly disagree
29) Do you consider this evidence to be clearly stated?	○	○	○	○	○
30) Do you agree with the way this evidence was stated?	○	○	○	○	○
31) Does this evidence align with your understanding, research, and/or practice?	○	○	○	○	○

32) Do you have any suggested edits to/ comments on this evidence section?

____

Connect communities through active outdoor play

Active outdoor play and community belonging, culture, and social connection are deeply intertwined, each reinforcing and enriching the other. Shared outdoor experiences help individuals build stronger communities, celebrate cultural traditions, and foster a sense of belonging3, 24-26, 64-71. In turn, vibrant communities create safe and supportive environments that encourage active outdoor play for all.

		Strongly agree	Somewhat agree	Neither agree nor disagree	Somewhat disagree	Strongly disagree
33)	Do you consider this evidence to be clearly stated?	○	○	○	○	○
34)	Do you agree with the way this evidence was stated?	○	○	○	○	○
35)	Does this evidence align with your understanding, research, and/or practice?	○	○	○	○	○

36) Do you have any suggested edits to/ comments on this evidence section? ________________________________

Beware of the alternative of the indoors

Active outdoor play is the ideal antidote to the alternative of being indoors. When indoors, people are less active and more sedentary, elevating the odds of developing chronic diseases and mental health problems72-81. They also accumulate excessive screen time and have increased exposure to indoor air pollutants, allergens, infectious diseases, and harmful chemicals, which can lead to chronic health conditions82-85.

	Strongly agree	Somewhat agree	Neither agree nor disagree	Somewhat disagree	Strongly disagree
37) Do you consider this evidence to be clearly stated?	○	○	○	○	○
38) Do you agree with the way this evidence was stated?	○	○	○	○	○
39) Does this evidence align with your understanding, research, and/or practice?	○	○	○	○	○

40) Do you have any suggested edits to/ comments on this evidence section? ______________________________

Expanding the Possibilities of Active Outdoor Play: Innovative Questions for Future Research, Policy, and Practice

As we push the boundaries of what we know about active outdoor play, new and urgent questions emerge-questions that challenge conventional thinking, spark curiosity, and open doors to transformative change. The following areas represent uncharted territories where research, policy, and practice must converge to shape the future of active outdoor play.

□□ Can active outdoor play reshape adult movement patterns?
How do 24-hour movement behaviours interact with active outdoor play in adults? Could outdoor play hold the key to enhancing physical and mental well-being across the lifespan?

□□ Could risky outdoor play be the secret ingredient for academic success?
What if taking risks outdoors-climbing higher, running faster, exploring the unknown-actually strengthens cognitive function and academic performance?

□□ What does active outdoor play look like beyond the Global North?
How do different cultural, environmental, and socio-political contexts shape the ways children engage in active outdoor play? What can we learn from regions where active outdoor play is less prevalent?

□□ How do we bridge the gap between research and real-world play?
Conceptually and empirically, what's missing between what we study and what happens on the ground? How can research better inform policies and practices that truly support active outdoor play?

□□ Can schools be redesigned and reprogrammed as playgrounds for movement, creativity, and learning?
How can we integrate active outdoor play into formal education systems-not just as a break from learning, but as an essential part of it? What would a school built for movement and play look like? Could school grounds serve a greater purpose as community outdoor play spaces?

□□ How do we design cities that invite play at every turn?
From shaded parks to healthy and playful streets, what urban design features make active outdoor play irresistible and inclusive for all?

□□ Could outdoor play be a game-changer for global sustainability?
What if active outdoor play held the key to tackling some of the world's biggest challenges-climate change, health inequities, and social justice? How does it align with the UN Sustainable Development Goals, and what policies could amplify its impact?

□□ Can playing outside protect us from the mental health risks of social media?
With rising concerns about screen time, anxiety, and loneliness, could active outdoor play be a powerful antidote to the negative effects of social media use? How can we reintroduce movement, nature, and face-to-face connection in a digital age?

Each of these questions holds the potential to disrupt conventional wisdom and reimagine the role of outdoor play in shaping healthier, more resilient individuals and communities. The next step? Bold research, innovative policy, and a commitment to action.

		Strongly agree	Somewhat agree	Neither agree nor disagree	Somewhat disagree	Strongly disagree
41)	Do you consider this section to be clearly stated?	◯	◯	◯	◯	◯
42)	Do you agree with the way this section is stated?	◯	◯	◯	◯	◯
43)	Does this section align with your understanding, research, and/or practice?	◯	◯	◯	◯	◯

44) Do you have any suggested edits to/ comments on this section? ________________________

Recommendations - For Sectors

To create systemic change that promotes active outdoor play, we need coordinated efforts across multiple sectors, including policy, education, environment, transportation, community, and research and development sectors.

Policy and Legislation

- Recognize active outdoor play as a fundamental right in health, education, and environmental policies at local, national, and global levels.

- Encourage organizations such as the World Health Organization and United Nations to integrate active outdoor play into policy frameworks.

- Expand funding for play-friendly infrastructure particularly in urban areas, including parks, trails, and nature-based play areas.

- Strengthen land-use policies to prioritize green and blue spaces in urban planning, ideally close to where people live.

- Prioritize and fund the development of pedestrian-friendly pathways, bike lanes, and green/blue spaces that connect neighborhoods, schools, recreational areas, and workplaces. Ensure that these spaces are well-lit, well-maintained, and safe for walking, wheeling, and cycling while accommodating other accessible forms of active transportation.

- Include children's and youth's voices in designing outdoor play spaces and child-friendly neighbourhoods, in line with the UN Sustainable Development Goals for Children, which emphasize the importance of including children's voices when developing plans to meet these goals.

- Locally, establish balanced liability frameworks that protect municipalities and organizations promoting active outdoor play while ensuring reasonable safety guidelines.

- Ensure that diverse groups are included in active outdoor play policy conversations. Diversity considerations include physical, visual, sensory and intellectual disabilities and accessibility among equity-denied communities.

	Strongly agree	Somewhat agree	Neither agree nor disagree	Somewhat disagree	Strongly disagree
45)					

		○	○	○	○	○
	Do you consider these recommendations to be clearly stated?	○	○	○	○	○
46)	Do you agree with the way these recommendations were stated?	○	○	○	○	○
47)	Do the recommendations align with your understanding, research, and/or practice?	○	○	○	○	○

48) Do you have any suggested edits to/ comments on this recommendations section? ______________________

Education and Schools

- Mandate daily active outdoor playtime in early childhood education and school curricula.

- Support educators and caregivers in understanding the benefits of active outdoor play and fostering risk-friendly environments that promote healthy life-long development.

- Promote outdoor classrooms and nature-based learning to encourage experiential education including post-secondary and adult education programs as well as community learning centres.

	Strongly agree	Somewhat agree	Neither agree nor disagree	Somewhat disagree	Strongly disagree
49) Do you consider these recommendations to be clearly stated?	○	○	○	○	○
50) Do you agree with the way these recommendations were stated?	○	○	○	○	○
51) Do the recommendations align with your understanding, research, and/or practice?	○	○	○	○	○

52) Do you have any suggested edits to/ comments on this recommendations section? ______________________

Urban and Community Planning

- Encourage local governments and organizations to design "playful" mobility initiatives, such as "walk-to-play" events or active travel programs, that highlight the benefits of combining active transportation with active outdoor play.

- Increase the availability of natural environments that encourage active outdoor play. This includes urban parks, green roofs, community gardens, and nature trails that promote physical activity while enhancing well-being, social connection, and environmental sustainability.

- Design play-friendly, accessible, and safe outdoor spaces in all neighbourhoods.

- Implement traffic-calming measures and car-free zones to create safer streets for play and active transportation in urban cities.

	Strongly agree	Somewhat agree	Neither agree nor disagree	Somewhat disagree	Strongly disagree
53) Do you consider these recommendations to be clearly stated?	○	○	○	○	○
54) Do you agree with the way these recommendations were stated?	○	○	○	○	○
55) Do the recommendations align with your understanding, research, and/or practice?	○	○	○	○	○

56) Do you have any suggested edits to/ comments on this reccomendations section? ______________________________

Public Awareness and Cultural Shift

- Support, promote, and build on existing global, national, and local campaigns that focus on shifting cultural attitudes towards the importance of active outdoor play as a lifelong healthy behaviour with numerous benefits to individuals of all ages and ecosystems. Examples of existing campaigns include the International Day of Play, Let Grow school programs, Take Me Outside Day, ISPAH's Eight Investments That Work for Physical Activity; and the World Health Organization (WHO)'s Global Action Plan on Physical Activity 2018-2030.

- Address concerns and promote benefit-risk approaches to active outdoor play for all ages, including adults and elderly.

- Encourage intergenerational active outdoor play to foster community connections.

	Strongly agree	Somewhat agree	Neither agree nor disagree	Somewhat disagree	Strongly disagree
57) Do you consider these recommendations to be clearly stated?	○	○	○	○	○
58) Do you agree with the way these recommendations were stated?	○	○	○	○	○
59) Do the recommendations align with your understanding, research, and/or practice?	○	○	○	○	○

60) Do you have any suggested edits to/ comments on this recommendations section? ______________________________

Media

- Shift the focus from indoor, screen-based leisure activities to the importance of outdoor playful activities and nature engagement.

- Challenge misconceptions about risk and safety by presenting balanced perspectives on benefit-risk approaches to active outdoor play.

- Highlight the impact of climate change on outdoor activities with adaptation strategies to keep cultural traditions alive- not just feature stories on how warming winters are affecting outdoor activities like ice skating, pond hockey, and cross-country skiing but cover how communities are adapting, such as creative uses of green spaces in changing climates and innovations like climate-resilient play spaces.

	Strongly agree	Somewhat agree	Neither agree nor disagree	Somewhat disagree	Strongly disagree
61) Do you consider these recommendation to be clearly stated?	○	○	○	○	○
62) Do you agree with the way these recommendations were stated?	○	○	○	○	○
63) Do the recommendations align with your understanding, research, and/or practice?	○	○	○	○	○

64) Do you have any suggested edits to/ comments on this recommendations section? ______________________

Research Monitoring System and Grant Agencies

- Invest in data collection and monitoring systems to track trends in outdoor play participation, identify gaps, and inform evidence-based policy decisions that promote equitable access.

- Establish standardized benchmarks and monitoring systems to track active outdoor play participation at national and global levels.

- Promote knowledge translation strategies to bridge research, policy, and practice, with the goal of fostering environments and initiatives that encourage active outdoor play for individuals of all ages.

		Strongly agree	Somewhat agree	Neither agree nor disagree	Somewhat disagree	Strongly disagree
65)	Do you consider these recommendations to be clearly stated?	○	○	○	○	○
66)	Do you agree with the way these recommendations were stated?	○	○	○	○	○
67)	Do the recommendations align with your understanding, research, and/or practice?	○	○	○	○	○

68) Do you have any suggested edits to/ comments on this recommendations section? ______________________

Recommendations - For Individuals and Communities

Along with the sector-specific recommendations, the promotion of active outdoor play must be accompanied by efforts at the individual and community levels, empowering people to integrate active outdoor play into daily life and fostering local communities to support active engagement.

Note: We acknowledge that individuals and communities, especially those from marginalized groups, can encounter multiple systemic barriers to accessing and engaging in active outdoor play. While these individual- and community-targeted recommendations do not specifically outline individual-level considerations for marginalized groups, they are incorporated into the broader sector-level recommendations.

Individuals and Families

- Active outdoor play isn't limited by age-it's a lifelong practice.

- Embrace active transportation and independent mobility opportunities as mechanisms for outdoor play experiences.

- Make active outdoor play a part of your daily routine. It's a fun way to get active and there are many opportunities to enjoy it in different outdoor spaces.

- Make it social! Encourage and invite others to join your active outdoor play routines-it can nourish a sense of community, connection, and vitality.

- Be weather-wise. Changing conditions like heat, wind, sun, and air pollution can affect outdoor activities. Stay informed with weather and air quality alerts to ensure safe participation.

	Strongly agree	Somewhat agree	Neither agree nor disagree	Somewhat disagree	Strongly disagree
69) Do you consider these recommendations to be clearly stated?	○	○	○	○	○
70) Do you agree with the way these recommendations were stated?	○	○	○	○	○

71)

Do the recommendations align with your understanding, research, and/or practice?	○	○	○	○	○

72) Do you have any suggested edits to/ comments on this recommendations section? __________________________________

Educators and Community Leaders

- Integrate active outdoor play into daily learning and community programmes.

- Champion inclusive and culturally relevant active outdoor play by ensuring that spaces and activities reflect the diverse backgrounds and abilities of your community.

- Support initiatives that model and advocate for playful learning and access to safe, outdoor play spaces.

- Equip families and communities with knowledge and resources by sharing guidance on weather preparedness, benefit-risk awareness, accessing outdoor play spaces, and the lifelong benefits of active outdoor play.

	Strongly agree	Somewhat agree	Neither agree nor disagree	Somewhat disagree	Strongly disagree
73) Do you consider these recommendations to be clearly stated?	○	○	○	○	○
74) Do you agree with the way these recommendations were stated?	○	○	○	○	○
75) Do the recommendations align with your understanding, research, and/or practice?	○	○	○	○	○

76) Do you have any suggested edits to/ comments on this recommendations section? __________________________________

Health Professionals and Public Health Advocates

- Prescribe active outdoor play as a vital component of maintaining good health. Advocate for active time outside to support/promote healthy lifestyles. For patients and individuals with diagnoses, develop personalized outdoor activity plans that consider specific health conditions, abilities, and safety needs. Collaborate with healthcare professionals, including physiotherapists and occupational therapists, to ensure appropriate modifications and support.

- Raise awareness of the health benefits of active outdoor play and educate medical trainees, patients, families, and communities about its role in reducing stress, improving sleep, and supporting overall health and wellbeing.

- Encourage time outside to help prevent diseases caused by sedentary lifestyles, screen overuse, and exposure to indoor pollutants.

- Advocate for policies that ensure all communities-regardless of income, ability, or location-have equitable access to green spaces and safe play-friendly environments.

	Strongly agree	Somewhat agree	Neither agree nor disagree	Somewhat disagree	Strongly disagree
77) Do you consider these recommendations to be clearly stated?	○	○	○	○	○
78) Do you agree with the way these recommendations were stated?	○	○	○	○	○
79) Do the recommendations align with your understanding, research, and/or practice?	○	○	○	○	○

80) Do you have any suggested edits to/ comments on this recommendations section? ______________________________

Researchers

- Conduct and disseminate research on the benefits, barriers, and long-term impacts of active outdoor play across all ages, integrating equity and accessibility considerations.

- Collaborate across fields such as public health, urban planning, environmental science, and psychology to inform intervention strategies to promote active outdoor play in different settings considering locality.

- Assess how policies, urban planning, and outdoor play space accessibility influence outdoor play opportunities and engagement.

- Examine how climate change-related factors (e.g., extreme heat, air pollution) interplay with active outdoor play and co-develop adaptation strategies with community and government partners.

		Strongly agree	Somewhat agree	Neither agree nor disagree	Somewhat disagree	Strongly disagree
81)	Do you consider these recommendations to be clearly stated?	○	○	○	○	○
82)	Do you agree with the way these recommendations were stated?	○	○	○	○	○
83)	Do the recommendations align with your understanding, research, and/or practice?	○	○	○	○	○

84) Do you have any suggested edits to/ comments on this recommendations section? __________________________________

Society and Collective Action

- Foster a culture that normalizes, values, and prioritizes active outdoor play.

- Create inclusive spaces where all individuals, regardless of age, background, or ability, can experience the benefits of active outdoor play.

- Recognize active outdoor play as a universal right and work across cultures and nations to promote policies and initiatives that enhance global access to active outdoor play.

- Advocate for collaboration between researchers, educators, urban planners, health professionals, and policymakers to make active outdoor play a public health priority.

	Strongly agree	Somewhat agree	Neither agree nor disagree	Somewhat disagree	Strongly disagree
85) Do you consider these recommendations to be clearly stated?	○	○	○	○	○
86) Do you agree with the way these recommendations were stated?	○	○	○	○	○
87) Do the recommendations align with your understanding, research, and/or practice?	○	○	○	○	○

88) Do you have any suggested edits to/ comments on this recommendations section? ______________________________

Survey Round 3

1. Please enter the access code that was provided to you with the survey link.

2025 Position Statement on Active Outdoor Play – Global Consensus Survey

Play-amble

The 2015 Position Statement on Active Outdoor Play[1] and its supporting evidence[2-3] showed the benefits of active outdoor play for children's health and well-being. Ten years later, research on this topic has increased by 10 times.[4] There has been more funding for outdoor play projects.[5] The Position Statement has influenced policy, research, and practice globally.[6] An international leadership group has come together to create the 2025 Position Statement on Active Outdoor Play to celebrate these achievements, update the evidence, and expand the scope. For example, the 2025 Position Statement is inclusive of all ages and is global in scope.

The 2025 Position Statement envisions a world where active outdoor play contributes to addressing global challenges such as health crises and climate change, while advancing the United Nations Sustainable Development Goals.[7] Together, as a collective of the outdoor play sector, we want to build healthier, more resilient communities by making active outdoor play part of daily life, ensuring that everyone has equitable access and opportunities to engage in active outdoor play.

This Position Statement calls for systemic change and offers recommendations for individuals, communities, and sectors. These recommendations aim to make active outdoor play a fundamental right and an essential part of sustainable societies.

To support this work, we conducted 10 systematic reviews and six continental narrative reviews, scanned relevant literature, and consulted global experts on nine key themes:

- *health and well-being*
- *One health*
- *nature and the environment*
- *human rights and policy*
- *community connections and partnerships*
- *social capital*
- *education and learning*
- *movement behaviors*
- *emerging areas*

See the links to the conceptual framework for this project and a full reference list at the bottom of this page.

Q1: Do you consider this section to be clearly stated?
R1: strongly agree/ agree/ neutral/ disagree/ strongly disagree

Q2: Do you agree with the way this section was stated?
R2: strongly agree/ agree/ neutral/ disagree/ strongly disagree
Q3: Does this section align with your understanding, research, and/or practice?
R3: strongly agree/ agree/ neutral/ disagree/ strongly disagree
Q4: Comments?

Here is the Proposed 2025 Position Statement:

Active outdoor play promotes holistic health and well-being for people of all ages, communities, and environments, and for our entire planet. It is critical given the multiple global challenges we face today (e.g., social and health inequities, climate change). Together, as a collective of the outdoor play sector, we recommend increasing opportunities for active outdoor play in all settings where people live, learn, work, and recreate. To achieve this, it is important to collaborate across sectors, settings, and societies to preserve, promote, and value equitable access to active play outdoors and in nature.

Q1: Do you consider this section to be clearly stated?
R1: strongly agree/ agree/ neutral/ disagree/ strongly disagree
Q2: Do you agree with the way this section was stated?
R2: strongly agree/ agree/ neutral/ disagree/ strongly disagree
Q3: Does this section align with your understanding, research, and/or practice?
R3: strongly agree/ agree/ neutral/ disagree/ strongly disagree
Q4: Comments?

[Starting on this page, we present key information nuggets derived from the work conducted for each theme. These are meant to be catchy and informative. After each statement, the descriptor paragraph provides more context with references, to highlight that the information is backed by evidence – additional reference suggestions are welcomed.]

Active Outdoor Play…

…helps us move more, play longer, and sleep better

When we play outdoors, we engage in more physical activity and less sedentary behavior and screen time, leading to better sleep.[8-16] Outdoor environments can entice individuals to engage in spontaneous, fun activities.

Q1: Do you consider this evidence to be clearly stated?
R1: strongly agree/ agree/ neutral/ disagree/ strongly disagree
Q2: Do you agree with the way this evidence was stated?
R2: strongly agree/ agree/ neutral/ disagree/ strongly disagree
Q3: Does this evidence align with your understanding, research, and/or practice?
R3: strongly agree/ agree/ neutral/ disagree/ strongly disagree
Q4: Comments?

...is a catalyst for sustaining health and well-being

Active outdoor play can enhance health and well-being (i.e., physical, mental, social, spiritual health).[17-25] Playing outdoors can offer an opportunity to connect, explore, and engage in healthy and enriching ways.[17,19,20]

Q1: Do you consider this evidence to be clearly stated?
R1: strongly agree/ agree/ neutral/ disagree/ strongly disagree
Q2: Do you agree with the way this evidence was stated?
R2: strongly agree/ agree/ neutral/ disagree/ strongly disagree
Q3: Does this evidence align with your understanding, research, and/or practice?
R3: strongly agree/ agree/ neutral/ disagree/ strongly disagree
Q4: Comments?

...facilitates diverse learning opportunities

Active outdoor play can unlock a world of rich, hands-on learning experiences, sparking curiosity, creativity, collaboration, and problem-solving.[26-29] It nurtures resilience and adaptability, and supports social, emotional, and cognitive growth throughout life.[30-33]

Q1: Do you consider this evidence to be clearly stated?
R1: strongly agree/ agree/ neutral/ disagree/ strongly disagree
Q2: Do you agree with the way this evidence was stated?
R2: strongly agree/ agree/ neutral/ disagree/ strongly disagree
Q3: Does this evidence align with your understanding, research, and/or practice?
R3: strongly agree/ agree/ neutral/ disagree/ strongly disagree
Q4: Comments?

...promotes confidence, naturally

Outdoor environments provide a dynamic space for playful adventures and risk-taking – an essential ingredient for healthy development and aging. Engaging in active outdoor play that is challenging helps build confidence, resilience, and problem-solving skills, while promoting agency, well-being, and physical literacy.[3, 23-25, 34-52]

Q1: Do you consider this evidence to be clearly stated?
R1: strongly agree/ agree/ neutral/ disagree/ strongly disagree
Q2: Do you agree with the way this evidence was stated?
R2: strongly agree/ agree/ neutral/ disagree/ strongly disagree
Q3: Does this evidence align with your understanding, research, and/or practice?
R3: strongly agree/ agree/ neutral/ disagree/ strongly disagree
Q4: Comments?

...offers a pathway to a happier, healthier planet

Active outdoor play can bridge multiple sectors including public health, education, recreation, and the environment, while recognizing the interconnectedness of human, animal, and environmental well-being.[53-54] It fosters environmental stewardship and enhances relationships with nature, contributing to healthier communities, stronger ecosystems, and a more sustainable planet. [1,55-58]

Q1: Do you consider this evidence to be clearly stated?
R1: strongly agree/ agree/ neutral/ disagree/ strongly disagree
Q2: Do you agree with the way this evidence was stated?
R2: strongly agree/ agree/ neutral/ disagree/ strongly disagree
Q3: Does this evidence align with your understanding, research, and/or practice?
R3: strongly agree/ agree/ neutral/ disagree/ strongly disagree
Q4: Comments?

…builds climate resilience and stewardship for a sustainable future

Engaging in active outdoor play nurtures a deepened connection with nature and caring for our natural world.[59-62] Climate change may threaten opportunities for active outdoor play. When active outdoor play is part of daily life, it can foster a culture where people of all ages play, learn, grow, and thrive together while enjoying the outdoors.
Q1: Do you consider this evidence to be clearly stated?
R1: strongly agree/ agree/ neutral/ disagree/ strongly disagree
Q2: Do you agree with the way this evidence was stated?
R2: strongly agree/ agree/ neutral/ disagree/ strongly disagree
Q3: Does this evidence align with your understanding, research, and/or practice?
R3: strongly agree/ agree/ neutral/ disagree/ strongly disagree
Q4: Comments?

…connects communities

Shared active outdoor play experiences build stronger communities, celebrate cultural traditions, and strengthen diversity while fostering a sense of belonging.[3, 23-25, 63-70] In turn, cohesive communities create safe and supportive environments that encourage citizenship, agency, and active outdoor play for all.

Q1: Do you consider this evidence to be clearly stated?
R1: strongly agree/ agree/ neutral/ disagree/ strongly disagree
Q2: Do you agree with the way this evidence was stated?
R2: strongly agree/ agree/ neutral/ disagree/ strongly disagree
Q3: Does this evidence align with your understanding, research, and/or practice?
R3: strongly agree/ agree/ neutral/ disagree/ strongly disagree
Q4: Comments?

…can reduce excessive indoor time

Excessive indoor time can contribute to prolonged sedentary behavior, increased screen time, and exposure to indoor pollutants, allergens, and infectious diseases. [71-78] Active outdoor play provides a healthy balance in reducing time spent indoors.

Q1: Do you consider this evidence to be clearly stated?
R1: strongly agree/ agree/ neutral/ disagree/ strongly disagree
Q2: Do you agree with the way this evidence was stated?
R2: strongly agree/ agree/ neutral/ disagree/ strongly disagree
Q3: Does this evidence align with your understanding, research, and/or practice?
R3: strongly agree/ agree/ neutral/ disagree/ strongly disagree
Q4: Comments?

Emerging Areas: Expanding the Possibilities of Active Outdoor Play

As we push the boundaries of what is known about active outdoor play, new and urgent questions arise that challenge conventional thinking, spark curiosity, and open doors to transformative change. The following areas represent uncharted territories where research, policy, and practice must converge to shape the future of active outdoor play.

🌍 Can active outdoor play reshape adult movement patterns? How do physical activity, sedentary behavior, and sleep patterns interact with active outdoor play in adults? Could outdoor play hold the key to enhancing physical and mental well-being across the lifespan?

🧠 Could risky outdoor play be a key ingredient for academic growth? Could taking risks outdoors – climbing higher, running faster, exploring the unknown – actually strengthen cognitive function and academic performance?

🌏 What does active outdoor play look like beyond the Global North? How do different cultural, environmental, and socio-political contexts shape the ways people engage in active outdoor play? What can we do to learn and share best practices from different regions and cultures?

🔬 How do we bridge the gap between research and real-world play? What's missing between what we study and what happens on the ground? How can research better inform policies and practices that support active outdoor play?

🏫 Can schools and schoolyards be redesigned and reprogrammed as playgrounds for movement, creativity, and learning? How can we integrate active outdoor play into formal education systems – not just as a break from learning, but as an essential part of it? What would a school built for movement and play look like? Could schoolyards serve a greater purpose as community outdoor play spaces?

🏙️ How do we design cities that invite play at every turn? From biodiverse urban parks to healthy and playful streets, what urban design features make active outdoor play irresistible and accessible? Inclusive for all?

🌱 Could active outdoor play be a conduit for global sustainability? What if active outdoor play held the key to tackling some of the world's biggest challenges – climate change, and health and social

inequities? How does it align with the UN Sustainable Development Goals, and what policies could amplify its impact?

📱With rising concerns about screen time, anxiety, and loneliness, could active outdoor play be a powerful antidote to the negative effects of social media use? How can we reintroduce movement, nature, and face-to-face connection in a digital age?

Each of these questions holds the potential to disrupt conventional wisdom and reimagine the role of outdoor play in shaping healthier, more resilient individuals and communities. The next step? Bold research, innovative policy, and a commitment to action!

Q1: Do you consider this section to be clearly stated?
R1: strongly agree/ agree/ neutral/ disagree/ strongly disagree
Q2: Do you agree with the way this section was stated?
R2: strongly agree/ agree/ neutral/ disagree/ strongly disagree
Q3: Does this section align with your understanding, research, and/or practice?
R3: strongly agree/ agree/ neutral/ disagree/ strongly disagree
Q4: Comments?

A Call to Action: Recommendations to Promote Active Outdoor Play

Societal Actions

- Encourage a culture that values and prioritizes active outdoor play as part of daily life.
- Create and improve access to spaces where everyone can enjoy active outdoor play.
- Advocate for collaboration among researchers, educators, urban planners, health professionals, and policy-makers to make active outdoor play a health priority.

Policy and Legislation

- Recognize access to active outdoor play as a fundamental right in health, education, recreation, and environmental policies.
- Encourage governments to enact and uphold policies that support active outdoor play.
- Promote, protect, preserve, and invest in outdoor play environments that connect neighborhoods, schools, recreational areas, and workplaces.

Education and Schools

- Encourage colleges, adult education centers, and community learning centers to integrate into programs ongoing professional development related to active outdoor play and learning.
- Require daily active outdoor playtime in early childhood education and K–12 school policies.
- Integrate outdoor classrooms and nature-based learning into education systems.

Public Health and Healthcare

- Educate medical professionals, patients, and communities on the health benefits of active outdoor play.
- Integrate active outdoor play into healthcare practice and public health initiatives to reduce sedentary behavior and improve health.
- Collaborate across sectors to develop localized public health strategies that promote active outdoor play.

Urban Planning

- Design accessible, safe, and play-friendly outdoor spaces in and around neighborhoods.
- Prioritize the preservation and restoration of natural environments that encourage active outdoor play when designing or redesigning communities.
- Reform municipal policies and bylaws to actively support and enable active outdoor play.

Community Leadership

- Support, promote, and build on campaigns that emphasize the importance of active outdoor play as a health-promoting habit.
- Support, promote, and build on efforts that promote benefit-risk approaches to active outdoor play.
- Encourage intergenerational active outdoor play to strengthen community connections.

Research and Surveillance

- Invest in data collection and monitoring systems to track trends in active outdoor play and to identify gaps.
- Explore the optimal quality and quantity of active outdoor play for healthy people and communities.
- Establish causal pathways between active outdoor play, and health and well-being outcomes.

Families

- Play with others, including companion animals, to nourish a sense of community and connection to the outdoors.
- Encourage and model active outdoor play as a normative behavior in your neighborhood.

- Support family members' participation in active outdoor play by encouraging, facilitating, and engaging together.

Individuals
- Be respectful stewards of the outdoor environments where play happens.
- Advocate for equitable access and preservation of green spaces and safe play-friendly environments.
- Explore and enjoy diverse experiences in different outdoor spaces as part of your daily routine.

Q1: Do you consider this section to be clearly stated?
R1: strongly agree/ agree/ neutral/ disagree/ strongly disagree
Q2: Do you agree with the way this section was stated?
R2: strongly agree/ agree/ neutral/ disagree/ strongly disagree
Q3: Does this section align with your understanding, research, and/or practice?
R3: strongly agree/ agree/ neutral/ disagree/ strongly disagree
Q4: Comments?

Other feedback

1. Do you feel this update to the Position Statement on Active Outdoor Play is important to public health?
    a. Yes
    b. No
    c. Unsure

2. Do you feel this update to the Position Statement on Active Outdoor Play is important to global and planetary health?
    a. Yes
    b. No
    c. Unsure

3. Do you feel this update to the Position Statement on Active Outdoor Play is important for meeting the United Nations Sustainable Development Goals?
    a. Yes
    b. No
    c. Unsure

4. Do you feel this update to the Position Statement on Active Outdoor Play important to you and/or your job?
    a. Yes
    b. No

    c. Unsure

5. Do you think the Position Statement is relevant/applicable to the country you reside in?
    a. Yes
    b. No
    c. Unsure

6. In the box below, please enter any additional comments you would like to add regarding this update to the Position Statement on Active Outdoor Play:
   (open box)

Demographic Information

7. Age
    a. <35 years
    b. 35-55 years
    c. >55 years
    d. Prefer not to answer

8. What sector do you represent/work in?
    a. Early childhood education
    b. Elementary to secondary school
    c. Health professional
    d. Allied health professional (e.g., public health professional, physiotherapist, nurse, dietitian, occupational therapist)
    e. Fitness professional (e.g., kinesiologist)
    f. Injury prevention
    g. Insurance
    h. Law
    i. Professor/researcher
    j. Non-governmental organization staff or board member
    k. Government
    l. Other, please specify:

9. In what country do you primarily live, work, and play?
   (drop down menu list of countries)

Recognizing your contribution

When the final version of the Position Statement on Active Outdoor Play is complete, would you like to be contacted to receive the final version, and if supportive, be listed as a supporter of the Position Statement?

a. Yes
b. No
c. Unsure

If yes, input your email here: ______________________________

*Note: This information is not connected to the consensus survey. Your responses to the consensus survey will remain anonymous if you provide your email.

. الرجاء إدخال رمز الوصول الذي تم تقديمه لك مع رابط الاستبيان.

المقدمة

أظهر بيان الموقف لعام ٢٠١٥ بشأن الألعاب والأنشطة في الهواء الطلق[1] والأدلة الداعمة له في هذا المجال[2-3] والفوائد العديدة لصحة الأطفال ورفاهيتهم. مع مرور عشر سنوات، زادت الأبحاث حول هذا الموضوع عشرة أضعاف [4]وكذلك زاد التمويل للمشاريع المتعلقة بالألعاب والأنشطة في الهواء الطلق[5].كان لبيان الموقف تأثير كبير في السياسات والأبحاث والممارسات على مستوى العالم.[6]
اجتمعت مجموعة قيادية دولية لإصدار بيان لعام ٢٠٢٥ حول الألعاب والأنشطة في الهواء الطلق، وذلك للاحتفاء بهذه الإنجازات، وتحديث الأدلة، وتوسيع نطاقها. فعلى سبيل المثال، يشمل بيان الموقف لعام ٢٠٢٥ جميع الأعمار، وهو ذو نطاق عالمي.

يتصوَّر بيان الموقف لعام ٢٠٢٥ عالمًا تساهم فيه الألعاب والأنشطة في الهواء الطلق في التصدي للتحديات العالمية مثل الأزمات الصحية ومختلف الظروف المناخية، مع تعزيز أهداف الأمم المتحدة للتنمية المستدامة.[7]
كمجموعة من قطاع الألعاب في الهواء الطلق، نريد معًا بناء مجتمعات أكثر صحة ومرونة من خلال جعل الألعاب والأنشطة في الهواء الطلق جزءاً من الحياة اليومية، وضمان حصول الجميع على فرص متكافئة للمشاركة في الألعاب والأنشطة في الهواء الطلق.

يدعو بيان الموقف هذا إلى تغيير منهجي، ويقدم توصيات للأفراد والمجتمعات والقطاعات. الهدف من هذه التوصيات هو جعل الألعاب والأنشطة في الهواء الطلق حقًا أساسيًا وجزءًا لا يتجزأ من المجتمعات المستدامة.

لدعم هذا المشروع، أجرينا عشر مراجعات منهجية وست مراجعات سردية على مستوى القارات، كما درسنا الأبحاث والأدبيات المرتبطة بالأمر، واستشرنا خبراء من مختلف أنحاء العالم حول تسعة مواضيع رئيسية:

- الصحة والرفاهية
- صحة واحدة
- الطبيعة والبيئة
- حقوق الإنسان والسياسات
- الروابط والشراكات المجتمعية
- رأس المال الاجتماعي
- التعليم والتعلم
- سلوكيات الحركة
- المجالات الناشئة

يرجى الاطلاع على روابط الإطار المفاهيمي لهذا المشروع وقائمة المراجع الكاملة في أسفل هذه الصفحة.

1Q: هل تعتبر هذا القسم واضح؟
1R: أوافق بشدة / أوافق / محايد / أختلف / أختلف بشدة
2Q: هل توافق على طريقة صياغة هذا القسم؟
2R: أوافق بشدة / أوافق / محايد / أختلف / أختلف بشدة
3Q: هل يتوافق هذا القسم مع فهمك وبحثك وممارستك العملية؟
3R: أوافق بشدة / أوافق / محايد / أختلف / أختلف بشدة
4Q: تعليقات؟

فيما يلي بيان الموقف المقترح لعام ٢٠٢٥:

إن الألعاب والأنشطة في الهواء الطلق يعزز الصحة والرفاهية الشاملة للأفراد من جميع الأعمار والمجتمعات والبيئات، ولكوكبنا بأكمله. وهو أمر بالغ الأهمية في ظل التحديات العالمية المتعددة التي نواجه اليوم (مثل التفاوتات الاجتماعية والصحية، وتغير المناخ).
معًا، كمجموعة من قطاع اللعب في الهواء الطلق، نوصي بزيادة فرص الألعاب والأنشطة في الهواء الطلق في جميع البيئات التي يعيش فيها الناس، ويتعلمون ويعملون ويستمتعون فيها. لتحقيق ذلك، من المهم التعاون بين القطاعات والبيئات والمجتمعات للحفاظ على فرص الألعاب والأنشطة في الهواء الطلق والطبيعة، وتعزيزها، وتقديرها.

Q1: هل تعتبر هذا القسم واضح؟
R1: أوافق بشدة / أوافق / محايد / أختلف / أختلف بشدة
Q2: هل توافق على طريقة صياغة هذا القسم؟
R2: أوافق بشدة / أوافق / محايد / أختلف / أختلف بشدة
Q3: هل يتوافق هذا القسم مع فهمك وبحثك وممارستك العملية؟
R3: أوافق بشدة / أوافق / محايد / أختلف / أختلف بشدة
Q4: تعليقات؟

-

[ابتداءً من هذه الصفحة، نقدم مقتطفات من المعلومات الأساسية المستمدة من العمل المنجز لكل موضوع. والمقصود منها أن تكون جذابة وغنية بالمعلومات. توفّر فقرة الوصف سياقًا معززًا بالمراجع بعد كل عبارة، للتأكد أن المعلومات مدعومة بالأدلة. يمكنك اقتراح أي مراجع إضافية.]

-

الألعاب والأنشطة في الهواء الطلق...

...يساعدنا على زيادة التحرك، واللعب لفترة أطول، والنوم بشكل أفضل

عندما نلعب في الهواء الطلق، نمارس النشاط البدني بشكل أكبر ونقلل من السلوكيات الخاملة والوقت الذي نقضيه في استخدام الشاشات، مما يؤدي إلى نوم أفضل. [16-18] كما أن البيئات الخارجية تشجع الأفراد على المشاركة في أنشطة عفوية وممتعة.

Q1: هل تعتبر هذا الدليل واضحًا؟
R1: أوافق بشدة / أوافق / محايد / أختلف / أختلف بشدة
Q2: هل توافق على الطريقة التي تم بها عرض هذا الدليل؟
R2: أوافق بشدة / أوافق / محايد / أختلف / أختلف بشدة
Q3: هل يتوافق هذا الدليل مع فهمك وبحثك وممارستك العملية؟
R3: أوافق بشدة / أوافق / محايد / أختلف / أختلف بشدة
Q4: تعليقات؟

...يعتبر حافزًا لاستدامة الصحة والرفاهية

الألعاب والأنشطة في الهواء الطلق قد يعزز من الصحة والرفاهية (أي الصحة البدنية والعقلية والاجتماعية والروحية).[17,25] كما أنه يوفر فرصة للتواصل مع الآخرين والاستكشاف والاشتراك بطرق صحية ومثرية.[17,19,20]

Q1: هل تعتبر هذا الدليل واضحًا؟
R1: أوافق بشدة / أوافق / محايد / أختلف / أختلف بشدة
Q2: هل توافق على الطريقة التي تم بها عرض هذا الدليل؟
R2: أوافق بشدة / أوافق / محايد / أختلف / أختلف بشدة
Q3: هل يتوافق هذا الدليل مع فهمك وبحثك وممارستك العملية؟
R3: أوافق بشدة / أوافق / محايد / أختلف / أختلف بشدة
Q4: تعليقات؟

...يوفّر فرص متنوعة لتعلم

الألعاب والأنشطة في الهواء الطلق ويفتح للفرد آفاقًا واسعة من تجارب عملية وثريّة للتعلم، ويثير الفضول والإبداع والتعاون وحل المشكلات.[26-29] كما أنه ينمّي المرونة والقدرة على التكيف، ويدعم النمو الاجتماعي والعاطفي والمعرفي طوال الحياة.[30-33]

Q1: هل تعتبر هذا الدليل واضحًا؟
R1: أوافق بشدة / أوافق / محايد / أختلف / أختلف بشدة
Q2: هل توافق على الطريقة التي تم بها عرض هذا الدليل؟
R2: أوافق بشدة / أوافق / محايد / أختلف / أختلف بشدة
Q3: هل يتوافق هذا الدليل مع فهمك وبحثك وممارستك العملية؟
R3: أوافق بشدة / أوافق / محايد / أختلف / أختلف بشدة
Q4: تعليقات؟

...يعزز الثقة بشكل طبيعي

ويوفر البيئات الخارجية والمساحة الديناميكية للمغامرات المرحة والمخاطرة، ويعتبر ذلك عنصرا أساسيا للنمو الصحي والشيخوخة. بالإضافة إلى ذلك، تمثل المشاركة في الألعاب والأنشطة في الهواء الطلق تحديًا وفرصة لبناء الثقة بالنفس، والمرونة، وتعزيز مهارات حل المشكلات، والقدرة على اتخاذ القرارات، والرفاهية، وتحسين المهارات البدنية.[3, 23-25, 34-52]

Q1: هل تعتبر هذا الدليل واضحًا؟
R1: أوافق بشدة / أوافق / محايد / أختلف / أختلف بشدة
Q2: هل توافق على الطريقة التي تم بها عرض هذا الدليل؟
R2: أوافق بشدة / أوافق / محايد / أختلف / أختلف بشدة
Q3: هل يتوافق هذا الدليل مع فهمك وبحثك وممارستك العملية؟
R3: أوافق بشدة/ أوافق/ محايد/ لا أوافق/ لا أوافق بشدة
Q4: تعليقات؟

...يوفر طريقًا إلى كوكب أكثر سعادة وصحة

يمكن للعب النشط في الهواء الطلق أن يربط بين قطاعات متعددة، بما في ذلك الصحة العامة والتعليم والترفيه والبيئة، مع مراعاة الترابط يمكن أن تساهم الألعاب والأنشطة في الهواء الطلق في الترابط بين قطاعات متعددة، بما في ذلك الصحة العامة، والتعليم، والترفيه، والبيئة.[53-54] ويعزز الإشراف البيئي ويقوي العلاقات مع والبيئة. وقد يساهم أيضًا في مراعاة التواصل بين رفاهية الإنسان والحيوان والبيئة الطبيعة، ويساعد في بناء مجتمعات صحية وأنظمة بيئية أقوى وكوكب مستدام.[1, 55-58]

1Q: هل تعتبر هذا الدليل واضحًا؟
1R: أوافق بشدة / أوافق / محايد / أختلف / أختلف بشدة
2Q: هل توافق على الطريقة التي تم بها عرض هذا الدليل؟
2R: أوافق بشدة / أوافق / محايد / أختلف / أختلف بشدة
3Q: هل يتوافق هذا الدليل مع فهمك وبحثك وممارستك العملية؟
3R: أوافق بشدة / أوافق / محايد / أختلف / أختلف بشدة
4Q: تعليقات؟

...يبني القدرة على التكيف مع المناخ والإشراف من أجل مستقبل مستدام

إن المشاركة في الألعاب والأنشطة في الهواء الطلق يغذي ارتباطًا عميقًا بالطبيعة والاهتمام بعالمنا الطبيعي. وقد يهدد تغير المناخ فرص الألعاب والأنشطة في الهواء الطلق.[59-62] عندما تكون الألعاب والأنشطة في الهواء الطلق جزءًا من الحياة اليومية، فإنه يعزز ثقافةً سالمة يلعب فيها الناس من جميع الأعمار، ويتعلمون، وينمون، ويزدهرون معًا أثناء الاستمتاع بالهواء الطلق.

1Q: هل تعتبر هذا الدليل واضحًا؟
1R: أوافق بشدة / أوافق / محايد / أختلف / أختلف بشدة
2Q: هل توافق على الطريقة التي تم بها عرض هذا الدليل؟
2R: أوافق بشدة / أوافق / محايد / أختلف / أختلف بشدة
3Q: هل يتوافق هذا الدليل مع فهمك وبحثك وممارستك العملية؟
3R: أوافق بشدة / أوافق / محايد / أختلف / أختلف بشدة
4Q: تعليقات؟

...يعزز الترابط بين المجتمعات

وبين تجارب الألعاب الخارجية والأنشطة المشتركة ويبني مجتمعات أقوى، تحتفي بالتقاليد الثقافية، وتعزز التنوع، ويعزز الشعور بالانتماء.[3, 23-25, 63-70] وفي المقابل، تهيئ المجتمعات المتماسكة بيئات آمنة وداعمة تشجع على المواطنة والمبادرة واللعب النشط في الهواء الطلق للجميع.

1Q: هل تعتبر هذا الدليل واضحًا؟
1R: أوافق بشدة / أوافق / محايد / أختلف / أختلف بشدة
2Q: هل توافق على الطريقة التي تم بها عرض هذا الدليل؟
2R: أوافق بشدة / أوافق / محايد / أختلف / أختلف بشدة
3Q: هل يتوافق هذا الدليل مع فهمك وبحثك وممارستك العملية؟
3R: أوافق بشدة / أوافق / محايد / أختلف / أختلف بشدة
4Q: تعليقات؟

...يمكن أن يقلل من الوقت الذي يقضيه الشخص في الأماكن المغلقة

وقد يُؤدي قضاء وقت طويل داخل المنزل وإطالة أمد السلوك الخامل، وزيادة وقت استخدام الشاشات، والتعرض للملوثات الداخلية، ومسببات الحساسية، والأمراض المعدية[71-78]. وتساعد الألعاب والأنشطة في الهواء الطلق على توفير توازن صحي من خلال تقليل الوقت الذي يقضيه الفرد داخل المنزل.

Q1: هل تعتبر هذا الدليل واضحًا؟
R1: أوافق بشدة / أوافق / محايد / أختلف / أختلف بشدة
Q2: هل توافق على الطريقة التي تم بها عرض هذا الدليل؟
R2: أوافق بشدة / أوافق / محايد / أختلف / أختلف بشدة
Q3: هل يتوافق هذا الدليل مع فهمك وبحثك وممارستك العملية؟
R3: أوافق بشدة / أوافق / محايد / أختلف / أختلف بشدة
Q4: تعليقات؟

المجالات الناشئة: توسيع إمكانيات الألعاب والأنشطة في الهواء الطلق

مع توسعنا في نطاق ما هو معروف عن الألعاب والأنشطة في الهواء الطلق، بروز أسئلة جديدة وملحّة تتحدى التفكير التقليدي، وتثير الفضول، وتفتح آفاقًا للتغيير الجذري. تمثل المجالات التالية مجالات جديدة لم تُستكشف بعد، حيث يجب أن تتضافر الأبحاث والسياسات والممارسات لصياغة مستقبل اللعب النشط في الهواء الطلق.

🌍 هل يمكن للعب النشط في الهواء الطلق أن يعيد تشكيل أنماط حركة البالغين؟ كيف يتفاعل النشاط البدني، والسلوكيات المستقرة، وأنماط النوم مع اللعب النشط لدى البالغين؟ هل يمكن للعب في الهواء الطلق أن يكون مفتاحًا لتحسين الصحة البدنية والعقلية طوال العمر؟

🧠 هل يمكن أن يكون اللعب في الهواء الطلق، الذي ينطوي على بعض المخاطر، عنصرًا أساسيًا في النمو الأكاديمي؟ هل يمكن للمخاطرة في الهواء الطلق – مثل التسلق إلى ارتفاعات عالية، والجري بسرعة أكبر، واستكشاف المجهول – أن تعزز الوظائف الإدراكية وتحسّن الأداء الأكاديمي؟

🌏 كيف يبدو اللعب النشط في الهواء الطلق خارج حدود الشمال العالمي؟ كيف تُشكّل السياقات الثقافية والبيئية والاجتماعية والسياسية المختلفة طرق مشاركة الناس في الألعاب والأنشطة في الهواء الطلق؟ ما الذي يمكننا فعله لتعلّم وتبادل أفضل الممارسات من مختلف المناطق والثقافات؟

🔬 كيف يمكننا سد الفجوة بين البحث العلمي في مجال اللعب والتطبيق على أرض الواقع؟ ما الذي ينقصنا بين ما ندرسه وما يحدث على أرض الواقع؟ كيف يمكن للأبحاث أن ترشد وتثري السياسات والممارسات الداعمة للألعاب والأنشطة في الهواء الطلق بشكل أفضل؟

🏫 هل يمكن إعادة تصميم المدارس وساحاتها وبرامجها لتصبح أماكن للحركة والإبداع والتعلم؟ كيف يمكن دمج الألعاب والأنشطة في الهواء الطلق ضمن أنظمة التعليم الرسمي – ليس فقط كاستراحة من التعلم، بل كجزء أساسي منه؟ كيف ستبدو المدرسة المصممة للحركة واللعب؟ هل يمكن أن تخدم ساحات المدارس غرضاً أكبر كمساحات لعب مجتمعية في الهواء الطلق؟

🏙️ كيف يمكننا أن نصمم مدنًا تدعو وتشجع على اللعب في كل زاوية؟ من الحدائق الحضرية المتنوعة بيولوجيًا إلى الشوارع الصحية والممتعة، ما هي سمات التصميم الحضري التي تجعل الألعاب والأنشطة في الهواء الطلق أمرًا لا يُقاوم ومتاحًا للجميع؟ وهل هو شامل لجميع الفئات؟

🌱 هل يمكن أن يكون اللعب النشط في الهواء الطلق وسيلةً للاستدامة العالمية؟ ماذا لو كان اللعب النشط في الهواء الطلق مفتاحًا لمعالجة بعضٍ من أكبر تحديات العالم – مثل تغير المناخ، والتفاوتات الصحية والاجتماعية؟ كيف يتماشى ذلك مع أهداف الأمم المتحدة للتنمية المستدامة، وما هي السياسات التي يمكنها تعزيز أثره؟

📱مع تزايد المخاوف حول الوقت الذي نقضيه أمام الشاشات، والإحساس بالقلق، والشعور بالوحدة، هل يمكن أن تكون الألعاب والأنشطة في الهواء الطلق علاجًا فعالًا للآثار السلبية الناتجة عن استخدام وسائل التواصل الاجتماعي؟ كيف يمكننا إعادة إحياء الحركة، والطبيعة، والتواصل المباشر في العصر الرقمي؟

💡 كل من هذه الأسئلة يحمل في طياته القدرة على تغيير المفاهيم الشائعة وإعادة تصوّر دور اللعب في الهواء الطلق في بناء أفراد ومجتمعات أكثر صحةً ومرونةً. فما هي الخطوة التالية؟ بحوث جريئة، سياسات مبتكرة، والتزام بالعمل!

Q1: هل تعتبر هذا القسم واضح؟
R1: أوافق بشدة / أوافق / محايد / أختلف / أختلف بشدة
Q2: هل توافق على طريقة صياغة هذا القسم؟
R2: أوافق بشدة / أوافق / محايد / أختلف / أختلف بشدة
Q3: هل يتوافق هذا القسم مع فهمك وبحثك وممارستك العملية؟
R3: أوافق بشدة / أوافق / محايد / أختلف / أختلف بشدة
Q4: تعليقات؟

دعوة إلى العمل: توصيات لتعزيز الألعاب والأنشطة في الهواء الطلق

الإجراءات المجتمعية

- تشجيع ثقافة تقدّر الألعاب والأنشطة في الهواء الطلق وتضعه ضمن أولوياتها كجزء من الحياة اليومية.
- إنشاء وتحسين إمكانية الوصول إلى الأماكن التي تتيح للجميع الاستمتاع باللعب النشط في الهواء الطلق.
- الدعوة إلى التعاون بين الباحثين، والمعلمين، ومخططي المدن، وأخصائي الصحة، وصانعي السياسات لجعل الألعاب والأنشطة في الهواء الطلق أولوية صحية لجميع أفراد المجتمع.

السياسات والتشريعات

● الاعتراف بإمكانية الوصول إلى الألعاب والأنشطة في الهواء الطلق كحق أساسي في سياسات الصحة والتعليم والترفيه والبيئة.
● تشجيع الحكومات على إصدار سياسات تدعم الألعاب والأنشطة في الهواء الطلق.
● تعزيز بيئات اللعب في الهواء الطلق، وحمايتها، والحفاظ عليها، والاستثمار فيها، بما يربط بين مختلف الضواحي والمدارس والمناطق الترفيهية وأماكن العمل.

التعليم والمدارس

- تشجيع الكليات ومراكز تعليم الكبار ومراكز التعليم المحلي على دمج التطوير المهني المستمر المتعلق باللعب النشط في الهواء الطلق في البرامج المتوفرة.
- تخصيص وقت يومي للألعاب والأنشطة في الهواء الطلق في سياسات المدارس من مرحلة الروضة حتى الصف الثاني عشر، واشتراط تضمينه في التعليم في مرحلة الطفولة المبكرة.
- إنشاء فصول دراسية في الهواء الطلق ودمج التعلم القائم على الطبيعة في النظم التعليمية.

الصحة العامة والرعاية الصحية

- تثقيف العاملين في المجال الطبي والمرضى والمجتمعات حول الفوائد الصحية للعب النشط في الهواء الطلق.
- دمج اللعب النشط في الهواء الطلق ضمن ممارسات الرعاية الصحية ومبادرات الصحة العامة للحد من السلوكيات التي تتسم بالخمول وقلة الحركة.
- التعاون بين مختلف القطاعات لتطوير استراتيجيات محلية للصحة العامة والتي تعزز اللعب النشط في الهواء الطلق.

التخطيط الحضري

- تصميم مساحات خارجية يسهل الوصول إليها، آمنة ومناسبة للعب في الأحياء السكنية والمناطق المحيطة بها.
- إعطاء الأولوية للحفاظ على البيئات الطبيعية التي تشجع على الألعاب والأنشطة في الهواء الطلق واستعادتها عند تصميم أو إعادة تصميم المجتمعات.
- إصلاح السياسات واللوائح البلدية لدعم وتمكين اللعب النشط في الهواء الطلق بشكل فعال.

القيادة المجتمعية

- دعم وتعزيز وبناء الحملات التي تركز على أهمية الألعاب والأنشطة في الهواء الطلق كعادة مفيدة للصحة.
- دعم وتعزيز الجهود التي تعزز مناهج تقييم الفوائد والمخاطر للعب النشط في الهواء الطلق، والبناء عليها.
- تشجيع اللعب النشط في الهواء الطلق بين الأجيال لتعزيز الروابط المجتمعية.

البحث والمراقبة

- الاستثمار في أنظمة جمع البيانات ورصدها لتتبع اتجاهات اللعب النشط في الهواء الطلق وتحديد الفجوات.
- استكشاف الجودة والكمية المثلى للعب النشط في الهواء الطلق من أجل صحة الأشخاص والمجتمعات.
- إنشاء مسارات سببية بين اللعب النشط في الهواء الطلق ونتائج الصحة والرفاهية.

العائلات

- اللعب مع الآخرين، بما في ذلك الحيوانات الأليفة، لتعزيز الشعور بالانتماء للمجتمع والتفاعل مع الهواء الطلق.
- تشجيع اللعب النشط في الهواء الطلق كسلوك معياري في المناطق السكنية.
- دعم مشاركة أفراد الأسرة في اللعب النشط في الهواء الطلق من خلال التشجيع والتسهيل والمشاركة معًا.

الأفراد

- أن نكون مشرفين محترمين على البيئات الخارجية التي يحدث فيها اللعب.
- الدعوة للوصول العادل إلى المساحات الخضراء والبيئات الآمنة المناسبة للعب، والحفاظ عليها.
- الاستكشاف والاستمتاع بالتجارب المتنوعة في المساحات الخارجية المختلفة كجزء من الروتين اليومي.

Q1: هل تعتبر هذا القسم واضح؟
R1: أوافق بشدة / أوافق / محايد / أختلف / أختلف بشدة
Q2: هل توافق على طريقة صياغة هذا القسم؟
R2: أوافق بشدة / أوافق / محايد / أختلف / أختلف بشدة
Q3: هل يتوافق هذا القسم مع فهمك وبحثك وممارستك العملية؟
R3: أوافق بشدة / أوافق / محايد / أختلف / أختلف بشدة
Q4: تعليقات؟

تعليقات أخرى

1. هل تعتقد أن هذا التحديث لبيان الموقف بشأن الألعاب والأنشطة في الهواء الطلق مهمة للصحة العامة؟

a. نعم

b. لا

c. غير متأكد

2. هل تعتقد أن هذا التحديث لبيان الموقف بشأن الألعاب والأنشطة في الهواء الطلق مهم للصحة العالمية والكوكب؟

a. نعم

b. لا

c. غير متأكد

3. هل تعتقد أن هذا التحديث لبيان الموقف بشأن الألعاب والأنشطة في الهواء الطلق مهم لتحقيق أهداف التنمية المستدامة للأمم المتحدة؟

a. نعم

b. لا

c. غير متأكد

4. هل تشعر أن هذا التحديث لبيان الموقف بشأن الألعاب والأنشطة في الهواء الطلق مهم بالنسبة لك و/أو لوظيفتك؟

a. نعم

b. لا

c. غير متأكد

5. هل تعتقد أن بيان الموقف مناسب/ينطبق على البلد الذي تقيم فيه؟

a. نعم

b. لا

c. غير متأكد

6. في المربع أدناه، يرجى إدخال أي تعليقات إضافية ترغب في إضافتها بخصوص هذا التحديث لبيان الموقف بشأن الألعاب والأنشطة في الهواء الطلق:

(open box)

المعلومات الديموغرافية

7. عمر

a. أقل من 35 سنة

b. سنة 35-55

c. سنة 55<

d. أفضل عدم الإجابة

8. ما هو القطاع الذي تمثله/تعمل فيه؟

a. تعليم الطفولة المبكرة

b. من المرحلة الابتدائية إلى الثانوية

c. أخصائي الصحة

d. متخصص في الرعاية الصحية المساعدة (على سبيل المثال، متخصص في الصحة العامة، أخصائي العلاج الطبيعي، الممرضة، أخصائي التغذية، أخصائي العلاج المهني)

e. متخصص في اللياقة البدنية (على سبيل المثال، أخصائي علم الحركة)

f. الوقاية من الإصابات

g. تأمين

h. قانون

i. أستاذ/باحث

j. موظف أو عضو مجلس إدارة منظمة غير حكومية

k. حكومة

l. أخرى، يرجى التحديد:

9. في أي بلد تعيش وتعمل وتلعب بشكل أساسي؟

(drop down menu list of countries)

تقديرًا لمساهمتك

عند اكتمال النسخة النهائية من بيان الموقف بشأن اللعب النشط في الهواء الطلق، هل ترغب في أن يتم التواصل معك لاستلام النسخة النهائية، وإذا كنت داعمًا، فهل ترغب في إدراج اسمك ضمن الداعمين لبيان الموقف؟

a. نعم

b. لا

c. غير متأكد

إذا كانت الإجابة بنعم، فأدخل بريدك الإلكتروني هنا: ________________________________

*ملاحظة: هذه المعلومات غير مرتبطة باستطلاع الإجماع. ستبقى إجاباتك على الاستطلاع مجهولة إذا زودتنا ببريدك الإلكتروني.

1. 请输入调查链接提供给您的访问代码。

2025 年关于积极户外活动的立场声明 – 全球共识调查

前言

2015年发表的《关于积极户外活动的立场声明》（以下简称2015立场声明）[1]及其证据[2-3]表明了积极的户外活动对儿童健康和福祉有益。十年后，关于这一主题的研究增加了十倍[4]，户外活动项目的资金投入也越来越多[5]。2015立场声明影响了全球的政策、研究和实践[6]。为庆祝这些成就、更新证据并扩大影响力，由一众国际专家牵头，制定了《2025关于积极户外活动的立场声明》（以下简称2025立场声明），更新的立场声明涵盖了全球所有年龄段的人群。

2025立场声明勾勒了全世界拥有积极的户外活动的蓝图，有助于应对健康危机和气候变化等全球挑战，同时推进联合国可持续发展的目标[4]。作为推动户外活动的团体，我们希望通过使积极的户外活动成为日常生活的一部分，从而建立更健康、更具韧性的社区，确保每个人都有平等的机会参与积极的户外活动。

2025立场声明呼吁系统性变革，并为个人、社区和各部门提供建议。这些建议旨在使积极的户外活动成为一项基本权利和可持续社会的重要组成部分。

为了支持这项工作，我们进行了十项系统综述和六项叙述性综述，检索了相关文献，并就九个关键主题咨询了全球专家：

- *健康和福祉*
- *一体健康*
- *自然与环境*
- *人权与政策*
- *社区联系和伙伴关系*
- *社会资本*
- *教育和学习*
- *身体活动行为*
- *新兴领域*

请参阅本页底部的项目概念框架及完整参考文献。

Q1：您认为本节内容表述清楚吗？
R1：非常同意/同意/中立/不同意/非常不同意
Q2：您是否同意本节的表述方式？
R2：非常同意/同意/中立/不同意/非常不同意
Q3：此部分是否与您的理解、研究和/或实践相符？
R3：非常同意/同意/中立/不同意/非常不同意

Q4：评论？

以下是拟议的 2025 年立场声明：

积极的户外活动能促进全人类（包括任何年龄段、社区、环境）及整个地球的健康和福祉。鉴于我们今天面临的多重全球挑战（例如社会和健康不平等、气候变化）——这一点至关重要。作为促进户外活动的团体，我们建议在人们生活、学习、工作和娱乐的所有环境中增加户外活动的机会。为了实现这一目标，跨部门、跨环境,和跨社会进行合作至关重要，以保护、促进和重视户外活动的平等机会。

Q1：您认为本节内容表述清楚吗？
R1：非常同意/同意/中立/不同意/非常不同意
Q2：您是否同意本节的表述方式？
R2：非常同意/同意/中立/不同意/非常不同意
Q3：此部分是否与您的理解、研究和/或实践相符？
R3：非常同意/同意/中立/不同意/非常不同意
Q4: 评论？

[从本页开始，我们将展示针对每个主题开展的工作得出的关键信息。这些信息旨在吸引人且信息丰富。在每个陈述之后，描述段落提供了更多带有参考资料的背景，以强调信息有证据支持——欢迎提供更多参考文献。]

积极的户外活动……

...帮助我们更多地运动、更长时间地玩耍和更好的睡眠

当我们在户外玩耍时，我们会进行更多的身体活动，减少久坐行为和屏幕时间，从而改善睡眠。户外环境可以吸引人们参与自发有趣的活动。

Q1：您认为这个证据表述清楚吗？
R1：非常同意/同意/中立/不同意/非常不同意
Q2：您是否同意该证据的陈述方式？
R2：非常同意/同意/中立/不同意/非常不同意
Q3：这个证据是否与您的理解、研究和/或实践相符？
R3：非常同意/同意/中立/不同意/非常不同意
Q4: 评论？

…是维持健康的催化剂

积极的户外活动可以促进健康（即身体、心理、社交和精神健康）。户外活动可以提供一个以健康和充实的方式进行交流、探索和参与的机会。

Q1：您认为这个证据表述清楚吗？
R1：非常同意/同意/中立/不同意/非常不同意
Q2：您是否同意该证据的陈述方式？

R2：非常同意/同意/中立/不同意/非常不同意

Q3：这个证据是否与您的理解、研究和/或实践相符？

R3：非常同意/同意/中立/不同意/非常不同意

Q4：评论？

...提供多样化的学习　机会

积极的户外活动可以开启一个丰富的动手学习体验的世界，激发好奇心、创造力、协作能力和解决问题的能力。它培养韧性和适应能力，并支持一生中社交、情感和认知的成长。

Q1：您认为这个证据表述清楚吗？

R1：非常同意/同意/中立/不同意/非常不同意

Q2：您是否同意该证据的陈述方式？

R2：非常同意/同意/中立/不同意/非常不同意

Q3：这个证据是否与您的理解、研究和/或实践相符？

R3：非常同意/同意/中立/不同意/非常不同意

Q4：评论？

...自然地增强信心

户外活动为孩子们提供了一个充满活力的探险和冒险空间，而这正是健康成长和衰老的必要因素。参与具有挑战性的户外活动有助于培养自信、韧性和解决问题的能力，同时促进行动、幸福感和身体素养。

Q1：您认为这个证据表述清楚吗？

R1：非常同意/同意/中立/不同意/非常不同意

Q2：您是否同意该证据的陈述方式？

R2：非常同意/同意/中立/不同意/非常不同意

Q3：这个证据是否与您的理解、研究和/或实践相符？

R3：非常同意/同意/中立/不同意/非常不同意

Q4：评论？

...为了更加幸福、健康的地球提供路径

积极的户外活动可以连接多个领域，包括公共卫生、教育、娱乐和环境，同时认识到人类、动物和环境福祉的相互联系。它促进环境管理并加强与自然的联系，有助于建立更健康的社区、更强大的生态系统和可持续发展的地球。

Q1：您认为这个证据表述清楚吗？

R1：非常同意/同意/中立/不同意/非常不同意

Q2：您是否同意该证据的陈述方式？

R2：非常同意/同意/中立/不同意/非常不同意

Q3：这个证据是否与您的理解、研究和/或实践相符？

R3：非常同意/同意/中立/不同意/非常不同意

Q4：评论？

...增强气候适应能力和管理能力，实现可持续未来

积极参与户外活动可以加深与自然的联系，关爱我们的自然世界。气候变化可能会威胁到积极参与户外活动的机会。当积极参与户外活动成为日常生活的一部分时，它可以培养出一种文化，让所有年龄段的人在享受户外活动的同时一起玩耍、学习和茁壮成长。

Q1：您认为这个证据表述清楚吗？
R1：非常同意/同意/中立/不同意/非常不同意
Q2：您是否同意该证据的陈述方式？
R2：非常同意/同意/中立/不同意/非常不同意
Q3：这个证据是否与您的理解、研究和/或实践相符？
R3：非常同意/同意/中立/不同意/非常不同意
Q4：评论？

...连接社区

共享的户外活动体验有助于建立更强大的社区、弘扬文化传统、加强多样性，同时培养归属感。反之，有凝聚力的社区会创造安全和支持性的环境，鼓励公民意识、行动和共同参与户外活动。

Q1：您认为这个证据表述清楚吗？
R1：非常同意/同意/中立/不同意/非常不同意
Q2：您是否同意该证据的陈述方式？
R2：非常同意/同意/中立/不同意/非常不同意
Q3：这个证据是否与您的理解、研究和/或实践相符？
R3：非常同意/同意/中立/不同意/非常不同意
Q4：评论？

…可以减少过多的室内时间

过多的室内时间会导致久坐行为、增加屏幕时间以及接触室内污染物、过敏原和传染病[71-78]。积极的户外活动可以减少在室内时间，从而达至平衡。

Q1：您认为这个证据表述清楚吗？
R1：非常同意/同意/中立/不同意/非常不同意
Q2：您是否同意该证据的陈述方式？
R2：非常同意/同意/中立/不同意/非常不同意
Q3：这个证据是否与您的理解、研究和/或实践相符？
R3：非常同意/同意/中立/不同意/非常不同意
Q4：评论？

新兴领域：拓展户外活动的可能性

随着我们不断拓展对户外运动的认知范围，新的紧迫问题不断涌现，它们挑战着传统思维，激发好奇心，并为变革打开大门。以下领域代表着未知领域，研究、政策和实践必须融合在一起，才能塑造户外活动的未来。

🌍积极的户外活动能否重塑成年人的运动模式？身体活动、久坐行为和睡眠模式如何与成年人的积极户外活动相互作用？户外活动能否成为促进终身身心健康的关键？

🧠户外冒险活动能否成为学业进步的关键因素？户外冒险——爬得更高、跑得更快、探索未知——是否真的能增强认知功能和学业成绩？

🌏全球北方以外的地区户外活动是什么样子的？不同的文化、环境和社会政治背景如何影响人们参与户外活动的方式？我们可以做些什么来学习和分享不同地区和文化的最佳实践？

🔬我们如何弥合研究与现实世界户外活动之间的差距？我们的研究和实际发生的事情之间缺少什么联系？研究如何更好地为支持积极户外活动的政策和实践提供证据？

🏫学校和校园能否重新设计和改造成为运动、创造和学习的游乐场？我们如何将积极的户外活动融入正规教育体系——不仅仅是学习中间的休息，而是学习的重要组成部分？一所为运动和户外活动而建的学校会是什么样子？校园能否作为社区户外活动空间发挥更大的作用？

🏙我们如何设计城市，让城市处处都充满户外活动的乐趣？从生物多样性的城市公园到健康有趣的街道，哪些城市设计特色能让户外活动变得不可抗拒且触手可及？包容所有人？

🌱积极的户外运动能否成为全球可持续发展的渠道？如果积极的户外活动是解决全球挑战（气候变化、健康和社会不平等）的关键，情况又会如何？它如何与联合国可持续发展目标保持一致？哪些政策可以扩大其影响力？

📱随着人们对屏幕时间、焦虑和孤独的担忧日益增加，积极的户外活动是否能有效缓解社交媒体带来的负面影响？在数字时代，我们如何才能重新引入运动、自然和面对面的交流？

这些问题中的每一个都有可能颠覆传统观念，重新构想户外活动在塑造更健康、更有韧性的个人和社区方面的作用。下一步是什么？大胆的研究、创新的政策和行动的承诺！

Q1：您认为本节内容表述清楚吗？
R1：非常同意/同意/中立/不同意/非常不同意
Q2：您是否同意本节的表述方式？
R2：非常同意/同意/中立/不同意/非常不同意
Q3：此部分是否与您的理解、研究和/或实践相符？
R3：非常同意/同意/中立/不同意/非常不同意

Q4：评论？

行动呼吁：促进户外活动的建议

社会行动

- 鼓励一种重视并优先考虑积极的户外活动作为日常生活的一部分的文化。
- 创建并改善每个人都可以享受户外活动的空间。
- 倡导研究人员、教育工作者、城市规划者、卫生专业人员和政策制定者之间的合作，使积极的户外活动成为健康的优先事项。

政策与立法

- 认识到参与积极的户外活动是健康、教育、娱乐和环境政策中的一项基本权利。
- 鼓励政府制定并坚持支持积极户外活动的政策。
- 促进、保护、维护和投资连接社区、学校、娱乐区和工作场所的户外游乐环境。

教育和学校

- 鼓励大学、成人教育中心和社区学习中心将与积极的户外活动和学习相关的持续专业发展融入计划中。
- 在早期儿童教育和 K-12 学校政策中，要求每天都有积极的户外活动时间。
- 将户外课堂和基于自然的学习融入教育体系。

公共卫生和医疗保健

- 向医疗专业人员、患者和社区宣传积极的户外活动对健康的益处。
- 将积极的户外活动融入医疗保健实践和公共卫生计划中，以减少久坐行为，并改善健康。
- 跨部门合作制定促进积极户外活动的本地化公共卫生策略。

城市规划

- 在社区内和周围设计方便、安全且适合玩耍的户外空间。
- 优先保护和恢复鼓励积极户外活动的自然环境。
- 改革市政政策和法规，积极支持和推动积极的户外活动。

社区领导力

- 支持、推广和开展强调积极户外活动作为促进健康习惯的重要性的活动。
- 支持、促进并加强推动积极户外活动的效益风险方法的努力。
- 鼓励跨代户外活动，以加强社区联系。

研究与监测

- 投资数据收集和监控系统来追踪户外活动的趋势并找出差距。
- 探索有利于人们和社区健康的户外活动的最佳质量和数量。
- 建立积极的户外活动与健康和福祉结果之间的因果关系。

家庭

- 与其他人一起玩耍，包括与宠物一起玩耍，以培养社区意识和与户外的联系。
- 鼓励并示范积极的户外活动作为您所在社区的规范行为。
- 通过鼓励、促进和共同参与来支持家庭成员参与积极的户外活动。

个人

- 尊重户外活动环境。
- 提倡公平地使用和保护绿色空间和安全的环境。
- 将探索和享受不同户外空间的多样化体验作为日常生活的一部分。

Q1：您认为本节内容表述清楚吗？
R1：非常同意/同意/中立/不同意/非常不同意
Q2：您是否同意本节的表述方式？
R2：非常同意/同意/中立/不同意/非常不同意
Q3：此部分是否与您的理解、研究和/或实践相符？
R3：非常同意/同意/中立/不同意/非常不同意
Q4: 评论？

其他反馈

1. 您认为户外活动立场声明的这一更新是否对公共卫生重要？

a. 是

b. 否

c. 不确定

2. 您认为户外活动立场声明的这一更新是否对全球和地球健康重要？

a. 是

b. 否

c. 不确定

3. 您认为户外活动立场声明的这一更新是否对实现联合国可持续发展目标重要？

a. 是

b. 否

c. 不确定

4. 您认为户外活动立场声明的这一更新是否对您和/或您的工作重要？

a.是

b. 否

c. 不确定

5. 您认为立场声明是否与您居住的国家相关/适用？

a. 是

b. 否

c. 不确定

6. 请在下方框中输入您对更新的积极户外活动立场声明的任何其他评论：

(open box)

人口统计信息

7. 年龄

a. <35 岁

b. 35-55 岁

c. >55 岁

d. 不愿回答

8. 您从事哪个行业的工作？

a. 幼儿教育

b. 中小学教育

c. 健康相关领域

d. 专职健康领域（例如公共卫生专业人员、理疗师、护士、营养师、职业治疗师）

e. 健身专业领域（例如运动机能学家）

f. 伤害预防

g. 保险

h. 法律

i. 教授/研究员

j. 非政府组织工作人员或董事会成员

k.政府

l. 其他，请说明：

9. 您主要在哪个国家生活、工作和活动？

(drop down menu list of countries)

您的贡献认可

当《积极户外活动立场声明》的最终版本完成后，您是否希望有人与您联系以接收最终版本，并且如果支持，是否希望被列为立场声明的支持者？

a. 是

b. 否

c. 不确定

如果是，请在此处输入您的电子邮件：________________________________

*注意：此信息与共识调查无关。如果您提供电子邮件，您对共识调查的回复将保持匿名。

1. Veuillez saisir le code d'accès qui vous a été fourni avec le lien de l'enquête.

Énoncé de position 2025 sur le jeu actif en plein air – Enquête de consensus mondiale

Préamble

La déclaration de position de 2015 sur le jeu actif en plein air [1] et les données probantes qui l'appuient [2-3] ont démontré les bienfaits du jeu actif en plein air pour la santé et le bien-être des enfants. Dix ans plus tard, la recherche sur ce sujet a décuplé [4]. Le financement des projets de jeu en plein air a augmenté [5.] Cet énoncé de position a influencé les politiques, la recherche et les pratiques à l'échelle mondiale [6.] Un groupe de direction international s'est réuni pour créer l'énoncé de position 2025 sur le jeu actif en plein air afin de célébrer ces réalisations, de mettre à jour les données probantes et d'élargir sa portée. Par exemple, la déclaration de position 2025 inclut tous les groupes d'âges et a une portée mondiale.

La déclaration de position 2025 envisage un monde où le jeu actif en plein air contribue à relever les défis mondiaux tels que les crises sanitaires et le changement climatique, tout en faisant progresser les Objectifs de développement durable des Nations Unies. [7] Ensemble, en tant que collectif du secteur du jeu en plein air, nous voulons bâtir des communautés plus saines et plus résilientes en intégrant le jeu actif en plein air à la vie quotidienne, en veillant à ce que chacun ait un accès équitable et des opportunités de jouer activement en plein air.

Cette déclaration de position appelle à un changement systémique et propose des recommandations aux individus, aux communautés et aux secteurs. Ces recommandations visent à faire du jeu actif en plein air un droit fondamental et un élément essentiel des sociétés durables.

Pour soutenir ce travail, nous avons mené 10 revues systématiques et six revues narratives continentales, analysé la littérature pertinente et consulté des experts mondiaux sur neuf thèmes clés :

- *santé et bien-être*
- *Une seule santé*
- *la nature et l'environnement*
- *droits humains et politiques*
- *liens et partenariats communautaires*
- *capital social*
- *éducation et apprentissage*
- *comportements de mouvement*
- *domaines émergents*

Voir les liens vers le cadre conceptuel de ce projet et une liste de référence complète au bas de cette page.

Q1 : Considérez-vous que cette section est clairement énoncée ?
R1 : tout à fait d'accord/d'accord/neutre/pas d'accord/pas du tout d'accord
Q2 : Êtes-vous d'accord avec la manière dont cette section a été formulée ?

R2 : tout à fait d'accord/d'accord/neutre/pas d'accord/pas du tout d'accord
Q3 : Cette section correspond-elle à votre compréhension, à vos recherches et/ou à votre pratique ?
R3 : tout à fait d'accord/d'accord/neutre/pas d'accord/pas du tout d'accord
Q4 : Commentaires ?

Voici l'énoncé de position 2025 :

Le jeu actif en plein air favorise la santé et le bien-être holistique pour les personnes de tous âges, communautés, environnements ainsi que de notre planète entière. Il est essentiel pour faire face aux multiples défis mondiaux auxquels nous sommes confrontés aujourd'hui (par exemple, inégalités sociales et sanitaires, changement climatique). Ensemble, en tant que collectif du secteur du jeu de plein air, nous recommandons d'accroître les possibilités de jeu actif en plein air dans tous les lieux de vie, d'apprentissage, de travail et de loisirs. Pour y parvenir, il est important de collaborer entre les secteurs, les milieux et les sociétés afin de préserver, de promouvoir et de valoriser un accès équitable au jeu actif en plein air et dans la nature.

Q1 : Considérez-vous que cette section est clairement énoncée ?
R1 : tout à fait d'accord/d'accord/neutre/pas d'accord/pas du tout d'accord
Q2 : Êtes-vous d'accord avec la manière dont cette section a été formulée ?
R2 : tout à fait d'accord/d'accord/neutre/pas d'accord/pas du tout d'accord
Q3 : Cette section correspond-elle à votre compréhension, à vos recherches et/ou à votre pratique ?
R3 : tout à fait d'accord/d'accord/neutre/pas d'accord/pas du tout d'accord
Q4 : Commentaires ?

[À partir de cette page, nous présentons des informations clés issues des travaux menés pour chaque thème. Elles se veulent accrocheuses et informatives. Après chaque énoncé, le paragraphe descriptif fournit davantage de contexte et des références, afin de souligner que les informations sont soutenues par des preuves ; toute suggestion de référence supplémentaire est la bienvenue.]

Jeu actif en plein air…

…nous aide à bouger plus, à jouer plus longtemps et à mieux dormir

Quand on joue à l'extérieur, nous pratiquons davantage d'activité physique et passons moins de temps sédentaire ou devant un écran, ce qui conduit à un meilleur sommeil. [8-16] Les environnements extérieurs permettent aux individus de pratiqué plus d'activités spontanées et agréables.

Q1 : Considérez-vous que cette preuve est clairement énoncée ?
R1 : tout à fait d'accord/d'accord/neutre/pas d'accord/pas du tout d'accord
Q2 : Êtes-vous d'accord avec la manière dont cette preuve a été présentée ?
R2 : tout à fait d'accord/d'accord/neutre/pas d'accord/pas du tout d'accord
Q3 : Ces preuves correspondent-elles à votre compréhension, à vos recherches et/ou à votre pratique ?

R3 : tout à fait d'accord/d'accord/neutre/pas d'accord/pas du tout d'accord
Q4 : Commentaires ?

...est un catalyseur pour soutenir la santé et le bien-être

Le jeu actif en plein air peut être bénéfique pour la santé et le bien-être physique, mental, social et spirituel. [17-25] Jouer en plein air peut offrir une occasion de se rencontrer, d'explorer et participer de manière saine et enrichissante. [17,19,20]

Q1 : Considérez-vous que cette preuve est clairement énoncée ?
R1 : tout à fait d'accord/d'accord/neutre/pas d'accord/pas du tout d'accord
Q2 : Êtes-vous d'accord avec la manière dont cette preuve a été présentée ?
R2 : tout à fait d'accord/d'accord/neutre/pas d'accord/pas du tout d'accord
Q3 : Ces preuves correspondent-elles à votre compréhension, à vos recherches et/ou à votre pratique ?
R3 : tout à fait d'accord/d'accord/neutre/pas d'accord/pas du tout d'accord
Q4 : Commentaires ?

...facilite diverses opportunités d' apprentissage

Le jeu actif en plein air peut ouvrir la voie à un monde d'expériences d'apprentissage riches et pratiques, susciter la curiosité, la créativité, la collaboration et la résolution de problèmes. [26-29] Il nourrit la résilience et l'adaptabilité et soutient la croissance sociale, émotionnelle et cognitive tout au long de la vie. [30-33]

Q1 : Considérez-vous que cette preuve est clairement énoncée ?
R1 : tout à fait d'accord/d'accord/neutre/pas d'accord/pas du tout d'accord
Q2 : Êtes-vous d'accord avec la manière dont cette preuve a été présentée ?
R2 : tout à fait d'accord/d'accord/neutre/pas d'accord/pas du tout d'accord
Q3 : Ces preuves correspondent-elles à votre compréhension, à vos recherches et/ou à votre pratique ?
R3 : tout à fait d'accord/d'accord/neutre/pas d'accord/pas du tout d'accord
Q4 : Commentaires ?

...favorise la confiance, naturellement

Les environnements extérieurs offrent un espace dynamique pour les aventures ludiques et la prise de risque – un élément essentiel à la croissance et au développement. Le jeu actif qui présente un défi aide à renforcer la confiance, la résilience et les compétences en résolution de problèmes, tout en favorisant le pouvoir d'action, le bien-être et le savoir-faire physique. [3, 23-25, 34-52]

Q1 : Considérez-vous que cette preuve est clairement énoncée ?
R1 : tout à fait d'accord/d'accord/neutre/pas d'accord/pas du tout d'accord
Q2 : Êtes-vous d'accord avec la manière dont cette preuve a été présentée ?
R2 : tout à fait d'accord/d'accord/neutre/pas d'accord/pas du tout d'accord
Q3 : Ces preuves correspondent-elles à votre compréhension, à vos recherches et/ou à votre pratique ?
R3 : tout à fait d'accord/d'accord/neutre/pas d'accord/pas du tout d'accord
Q4 : Commentaires ?

...offre une voie vers une planète plus heureuse et plus saine

Le jeu actif en plein air peut construire des ponts entre plusieurs secteurs incluant la santé publique, l'éducation, les loisirs, et l'environnement en reconnaissant les interconnexions entre la santé humaine, animale et environnementale [53-54] Il favorise la protection de l'environnement et la connexion avec la nature contribuant à bâtir des communautés plus saines, des écosystèmes plus forts et une planète plus durable.

Q1 : Considérez-vous que cette preuve est clairement énoncée ?
R1 : tout à fait d'accord/d'accord/neutre/pas d'accord/pas du tout d'accord
Q2 : Êtes-vous d'accord avec la manière dont cette preuve a été présentée ?
R2 : tout à fait d'accord/d'accord/neutre/pas d'accord/pas du tout d'accord
Q3 : Ces preuves correspondent-elles à votre compréhension, à vos recherches et/ou à votre pratique ?
R3 : tout à fait d'accord/d'accord/neutre/pas d'accord/pas du tout d'accord
Q4 : Commentaires ?

...renforce la résilience climatique et la gestion responsable pour un avenir durable

Le jeu actif en plein air favorise le développement d'une connexion profonde avec la nature et la bienveillance envers notre monde naturel. [59-62] Le changement climatique pourrait menacer les possibilités de jeu actif en plein air. Lorsque le jeu actif en plein air fait partie de la vie quotidienne, il peut créer une culture ou les gens de tous les groupes d'âge jouent, apprennent, grandissent et s'épanouissent ensemble tout en appréciant la nature

Q1 : Considérez-vous que cette preuve est clairement énoncée ?
R1 : tout à fait d'accord/d'accord/neutre/pas d'accord/pas du tout d'accord
Q2 : Êtes-vous d'accord avec la manière dont cette preuve a été présentée ?
R2 : tout à fait d'accord/d'accord/neutre/pas d'accord/pas du tout d'accord
Q3 : Ces preuves correspondent-elles à votre compréhension, à vos recherches et/ou à votre pratique ?
R3 : tout à fait d'accord/d'accord/neutre/pas d'accord/pas du tout d'accord
Q4 : Commentaires ?

...connecte les communautés

Les expériences collectives de jeu actif en plein air renforcent les communautés, célèbrent les traditions culturelles et renforcent la diversité tout en favorisant un sentiment d'appartenance. [3, 23-25, 63-70] À leur tour, les communautés cohésives créent des environnements sûrs et favorables qui encouragent la citoyenneté, le pouvoir d'agir et le jeu actif en plein air pour tous.

Q1 : Considérez-vous que cette preuve est clairement énoncée ?
R1 : tout à fait d'accord/d'accord/neutre/pas d'accord/pas du tout d'accord
Q2 : Êtes-vous d'accord avec la manière dont cette preuve a été présentée ?
R2 : tout à fait d'accord/d'accord/neutre/pas d'accord/pas du tout d'accord
Q3 : Ces preuves correspondent-elles à votre compréhension, à vos recherches et/ou à votre pratique ?
R3 : tout à fait d'accord/d'accord/neutre/pas d'accord/pas du tout d'accord

Q4 : Commentaires ?

...peut réduire le temps excessif passé à l'intérieur

Un temps excessif passé à l’intérieur peut contribuer à un comportement sédentaire prolongé, à une augmentation du temps d’écran et à l’exposition aux polluants intérieurs, aux allergènes et aux maladies infectieuses.[71-78] Le jeu actif en plein air offre un équilibre sain en réduisant le temps passé à l’intérieur.

Q1 : Considérez-vous que cette preuve est clairement énoncée ?
R1 : tout à fait d'accord/d'accord/neutre/pas d'accord/pas du tout d'accord
Q2 : Êtes-vous d’accord avec la manière dont cette preuve a été présentée ?
R2 : tout à fait d'accord/d'accord/neutre/pas d'accord/pas du tout d'accord
Q3 : Ces preuves correspondent-elles à votre compréhension, à vos recherches et/ou à votre pratique ?
R3 : tout à fait d'accord/d'accord/neutre/pas d'accord/pas du tout d'accord
Q4 : Commentaires ?

Domaines émergents : élargir les possibilités de jeu actif en plein air

Alors que nous repoussons les limites de nos connaissances sur le jeu actif en plein air, de nouvelles questions urgentes surgissent, remettant en question les idées reçues, stimulant la curiosité et ouvrant la voie à des changements transformateurs. Les domaines suivants représentent des territoires inexplorés où la recherche, les politiques et la pratique doivent converger pour façonner l'avenir du jeu actif en plein air.

🌍 Le jeu actif en plein air peut-il modifier les comportements de mouvement des adultes ? Comment l'activité physique, la sédentarité et les habitudes de sommeil interagissent-elles avec le jeu actif en plein air chez les adultes ? Le jeu en plein air pourrait-il être la clé pour améliorer le bien-être physique et mental tout au long de la vie ?

🧠 Le jeu risqué à l’extérieur pourraient-il être un facteur clé de réussite scolaire ? Prendre des risques à l’extérieur – grimper plus haut, courir plus vite, explorer l'inconnu – pourrait-il réellement renforcer les fonctions cognitives et les performances scolaires ?

🌏 À quoi ressemble le jeu actif en plein air au-delà des pays du nord ? Comment les différents contextes culturels, environnementaux et sociopolitiques influencent-ils la manière dont les gens s'adonnent au jeu actif en plein air ? Comment pouvons-nous apprendre et partager les meilleures pratiques des différentes régions et cultures ?

🔬 Comment combler l’écart entre la recherche et le jeu en situation réelle ? Quel est l’écart entre ce que nous étudions et la réalité du terrain ? Comment la recherche peut-elle mieux éclairer les politiques et les pratiques qui favorisent le jeu actif en plein air ?

🏫 Les écoles et les cours d'école peuvent-elles être repensées et réaménagées pour devenir des terrains de jeu favorisant le mouvement, la créativité et l'apprentissage ? Comment intégrer le jeu actif en plein air dans les systèmes éducatifs formels, non seulement comme une pause dans l'apprentissage, mais comme un élément essentiel de celui-ci ? À quoi ressemblerait une école conçue pour le mouvement et le jeu ? Les cours d'école pourraient-elles aussi servir d'espaces de jeu communautaires en plein air ?

🏙️ Comment concevoir des villes qui invitent au jeu à chaque instant ? Des parcs urbains riches en biodiversité aux rues saines et ludiques, quels éléments d'aménagement urbain rendent le jeu actif en plein air irrésistible et accessible ? Inclusif pour tous ?

🌱 Le jeu actif en plein air pourrait-il contribuer à la durabilité mondiale ? Et si le jeu actif en plein air était la clé pour relever certains des plus grands défis mondiaux : le changement climatique et les inégalités sociales et sanitaires ? Comment s'inscrit-il dans les Objectifs de développement durable des Nations Unies et quelles politiques pourraient amplifier son impact ?

📱 Face aux préoccupations croissantes concernant le temps passé devant les écrans, l'anxiété et la solitude, le jeu actif en plein air pourrait-il être un puissant antidote aux effets négatifs de l'utilisation des réseaux sociaux ? Comment pouvons-nous réintroduire le mouvement, la nature et les interactions en face à face à l'ère du numérique ?

Chacune de ces questions a le potentiel de bousculer les idées reçues et de repenser le rôle du jeu en plein air dans la formation d'individus et de communautés plus sains et plus résilients. La prochaine étape ? Des recherches audacieuses, des politiques innovantes et un engagement à agir !

Q1 : Considérez-vous que cette section est clairement énoncée ?
R1 : tout à fait d'accord/d'accord/neutre/pas d'accord/pas du tout d'accord
Q2 : Êtes-vous d’accord avec la manière dont cette section a été formulée ?
R2 : tout à fait d'accord/d'accord/neutre/pas d'accord/pas du tout d'accord
Q3 : Cette section correspond-elle à votre compréhension, à vos recherches et/ou à votre pratique ?
R3 : tout à fait d'accord/d'accord/neutre/pas d'accord/pas du tout d'accord
Q4 : Commentaires ?

Un appel à l'action : recommandations pour promouvoir le jeu actif en plein air

Actions sociétales

- Encourager une culture qui valorise et donne la priorité au jeu actif en plein air dans le cadre de la vie quotidienne.
- Créer et améliorer l’accès à des espaces où chacun peut profiter de jeux actifs en plein air.

- Plaider en faveur d’une collaboration entre les chercheurs, les éducateurs, les urbanistes, les professionnels de la santé et les décideurs politiques pour faire du jeu actif en plein air une priorité en matière de santé.

Politique et législation

- Reconnaître l’accès au jeu actif en plein air comme un droit fondamental dans les politiques de santé, d’éducation, de loisirs et d’environnement.
- Encourager les gouvernements à adopter et à maintenir des politiques qui soutiennent le jeu actif en plein air.
- Promouvoir, protéger, préserver et investir dans des environnements de jeu extérieurs qui relient les quartiers, les écoles, les zones de loisirs et les lieux de travail.

Éducation et écoles

- Encourager les collèges, les centres d’éducation pour adultes et les centres d’apprentissage communautaires à intégrer dans leurs programmes un développement professionnel continu lié au jeu et à l’apprentissage actifs en plein air.
- Exiger des périodes de jeu actif quotidiennes en plein air dans les politiques d’éducation de la petite enfance et des écoles primaires et secondaires.
- Intégrer les classes en plein air et l’apprentissage basé sur la nature dans les systèmes éducatifs.

Santé publique et soins de santé

- Sensibiliser les professionnels de la santé, les patients et les communautés aux bienfaits pour la santé du jeu actif en plein air.
- Intégrer le jeu actif en plein air dans les pratiques de soins de santé et les initiatives de santé publique pour réduire les comportements sédentaires et améliorer la santé.
- Collaborer entre les secteurs pour développer des stratégies de santé publique localisées qui favorisent le jeu actif en plein air.

Urbanisme

- Concevoir des espaces extérieurs accessibles, sûrs et propices aux jeux dans et autour des quartiers.
- Accorder la priorité à la préservation et à la restauration des environnements naturels qui encouragent le jeu actif en plein air lors de la conception ou du réaménagement des communautés.
- Réformer les politiques et les règlements municipaux pour soutenir et faciliter le jeu actif en plein air.

Leadership communautaire

- Soutenir, promouvoir et développer des campagnes qui soulignent l’importance du jeu actif en plein air comme habitude bénéfique pour la santé.

- Soutenir, promouvoir et développer les efforts qui favorisent les approches avantages-risques du jeu actif en plein air.

Encouragez le jeu actif en plein air intergénérationnel pour renforcer les liens communautaires.

Recherche et surveillance

- Investir dans des systèmes de collecte et de surveillance des données pour suivre les tendances en matière de jeu actif en plein air et pour identifier les lacunes.
- Explorer la qualité et la quantité optimales de jeu actif en plein air pour la santé des personnes et des communautés.
- Établir des liens de causalité entre le jeu actif en plein air et les résultats en matière de santé et de bien-être.

Familles

- Jouer avec d'autres personnes, y compris des animaux de compagnie, pour nourrir un sentiment de communauté et de connexion avec l'extérieur.
- Encourager et modéliser le jeu actif en plein air comme un comportement normatif dans votre quartier.
- Soutenir la participation des membres de la famille aux jeu actif en plein air en les encourageant, en les facilitant et en participant ensemble.

Individus

- Soyez des intendants respectueux des environnements extérieurs où se déroulent les jeux.
- Plaider pour un accès équitable et la préservation des espaces verts et des environnements de jeu sûrs.
- Explorer et profiter d'expériences diverses dans différents espaces extérieurs dans le cadre de votre routine quotidienne.

Q1 : Considérez-vous que cette section est clairement énoncée ?

R1 : tout à fait d'accord/d'accord/neutre/pas d'accord/pas du tout d'accord

Q2 : Êtes-vous d'accord avec la manière dont cette section a été formulée ?

R2 : tout à fait d'accord/d'accord/neutre/pas d'accord/pas du tout d'accord

Q3 : Cette section correspond-elle à votre compréhension, à vos recherches et/ou à votre pratique ?

R3 : tout à fait d'accord/d'accord/neutre/pas d'accord/pas du tout d'accord

Q4 : Commentaires ?

Autres commentaires

1. Pensez-vous que cette mise à jour de l'Énoncé de position 2025 sur le jeu actif en plein air est important pour la santé publique ?

a. Oui

b. Non

c. Incertain

2. Pensez-vous que cette mise à jour de l'Énoncé de position 2025 sur le jeu actif en plein air est important pour la santé mondiale et planétaire ?

a. Oui

b. Non

c. Incertain

3. Pensez-vous que cette mise à jour de l'Énoncé de position 2025 sur le jeu actif en plein air est important pour atteindre les Objectifs de développement durable des Nations Unies ?

a. Oui

b. Non

c. Incertain

4. Pensez-vous que cette mise à jour de l'Énoncé de position 2025 sur le jeu actif en plein air est important pour vous et/ou votre travail ?

a. Oui

b. Non

c. Incertain

5. Pensez-vous que l'Énoncé de position 2025 sur le jeu actif en plein air est pertinent/applicable à votre pays de résidence ?

a. Oui

b. Non

c. Incertain

6. Dans la case ci-dessous, veuillez indiquer tout commentaire supplémentaire que vous souhaiteriez ajouter concernant cette mise à jour de l'Énoncé de position sur le jeu actif en plein air :

(open box)

Informations démographiques

7. Âge

a. < 35 ans

b. 35-55 ans

c. > 55 ans

d. Je préfère ne pas répondre

8. Quel secteur représentez-vous/dans quel secteur travaillez-vous ?

a. Éducation de la petite enfance

b. École primaire à secondaire

c. Professionnel de la santé

d. Professionnel paramédical (p. ex., professionnel de la santé publique, physiothérapeute, infirmier, diététicien, ergothérapeute)

e. Professionnel du conditionnement physique (p. ex., kinésiologue)

f. Prévention des blessures

g. Assurances

h. Droit

i. Professeur/chercheur

j. Personnel ou membre du conseil d'administration d'une organisation non gouvernementale

k. Gouvernement

l. Autre, veuillez préciser :

9. Dans quel pays vivez-vous, travaillez-vous et jouez-vous principalement ?

(drop down menu list of countries)

Reconnaissance de votre contribution

Une fois la version finale de l'Énoncé de position 2025 sur le jeu actif en plein air terminé, souhaitez-vous être contacté pour le recevoir et, si vous le soutenez,souhaitez-vous figurer sur la liste des signataires ?

a. Oui

b. Non

c. Incertain

Si oui, veuillez indiquer votre adresse courriel ici : ______________________________

*Remarque : Ces informations ne sont pas liées à l'enquête de consensus. Vos réponses à l'enquête de consensus resteront anonymes si vous indiquez votre adresse courriel.

1. Введите код доступа, который был предоставлен вам вместе со ссылкой на опрос.

Позиционное заявление 2025 года об активных играх на свежем воздухе – Глобальный консенсусный опрос

Введение

Позиционное заявление 2015 года об активных играх на свежем воздухе [1] и его подтверждающие доказательства [2-3] продемонстрировали пользу активных игр на свежем воздухе для здоровья и благополучия детей. Спустя десять лет количество исследований по этой теме увеличились в 10 раз. [4] Увеличилось финансирование проектов по играм на свежем воздухе.[5] Позиционное заявление повлияло на политику, исследования и практику во всем мире. [6] Международная группа лидеров объединилась для создания Позиционного заявления 2025 года об активных играх на свежем воздухе, чтобы отметить эти достижения, обновить доказательства и расширить сферу действия. Например, Позиционное заявление 2025 года охватывает все возрасты и имеет глобальный охват

В позиционном заявлении 2025 года описывается мир, в котором активные игры на свежем воздухе способствуют решению глобальных проблем, таких как кризисы в области здравоохранения и изменение климата, а также достижению Целей Организации Объединенных Наций в области устойчивого развития . [7] Вместе, как коллектив сектора игр на свежем воздухе, мы хотим создать более здоровые и устойчивые сообщества, сделав активные игры на свежем воздухе частью повседневной жизни, обеспечивая каждому равный доступ и возможности для участия в активных играх на свежем воздухе.

Это позиционное заявление призывает к системным изменениям и предлагает рекомендации для отдельных лиц, сообществ и секторов. Эти рекомендации направлены на то, чтобы сделать активные игры на свежем воздухе основополагающим правом и неотъемлемой частью устойчивого общества.

Для поддержки этой работы мы провели 10 систематических обзоров и шесть континентальных нарративных обзоров, изучили соответствующую литературу и проконсультировались с мировыми экспертами по девяти ключевым темам:

- *здоровье и благополучие*
- *Единое здоровье*
- *природа и окружающая среда*
- *права человека и политика*
- *общественные связи и партнерства*
- *социальный капитал*
- *образование и обучение*
- *двигательное поведение*
- *развивающиеся области*

Ссылки на концептуальную основу этого проекта и полный список литературы можно найти внизу этой страницы.

В1: Считаете ли вы, что этот раздел изложен ясно?
R1: полностью согласен/ согласен/ нейтрален/ не согласен/ категорически не согласен
В2: Согласны ли вы с тем, как был изложен этот раздел?
R2: полностью согласен/ согласен/ нейтрален/ не согласен/ категорически не согласен
В3: Соответствует ли этот раздел вашему пониманию, исследованиям и/или практике?
R3: полностью согласен/ согласен/ нейтрален/ не согласен/ категорически не согласен
В4: Комментарии?

Предлагаемое Позиционное заявление 2025 года:

Активные игры на свежем воздухе способствуют общему здоровью и благополучию людей всех возрастов, сообществ, сред и всей нашей планеты. Это критически важно, учитывая многочисленные глобальные проблемы, с которыми мы сталкиваемся сегодня (например, социальное неравенство, неравенство в состоянии здоровья, изменение климата). Вместе, как коллектив сектора игр на свежем воздухе, мы рекомендуем расширять возможности для активных игр на свежем воздухе везде, где люди живут, учатся, работают и отдыхают. Для достижения этой цели, важно сотрудничество между секторами, сферами и обществами, чтобы сохранять, развивать и ценить равноправный доступ к активным играм на свежем воздухе и на природе.

В1: Считаете ли вы, что этот раздел изложен ясно?
R1: полностью согласен/ согласен/ нейтрален/ не согласен/ категорически не согласен
В2: Согласны ли вы с тем, как был изложен этот раздел?
R2: полностью согласен/ согласен/ нейтрален/ не согласен/ категорически не согласен
В3: Соответствует ли этот раздел вашему пониманию, исследованиям и/или практике?
R3: полностью согласен/ согласен/ нейтрален/ не согласен/ категорически не согласен
В4: Комментарии?

[Начиная с этой страницы, мы представляем ключевые информационные моменты, полученные в результате работы, проведенной по каждой теме. Предусматривается, что они должны быть привлекательными и информативными. После каждого утверждения идет пояснительный абзац с дополнительным контекстом и ссылками, чтобы подчеркнуть, что информация подкреплена доказательствами — дополнительные предложения по ссылкам приветствуются.]

Активные игры на свежем воздухе...

...помогают нам двигаться больше, играть дольше и спать лучше

Когда мы играем на свежем воздухе, мы больше занимаемся физической активностью, меньше ведем сидячий образ жизни и проводим меньше времени перед экраном, что способствует лучшему сну. [8–16] Окружающая среда может побудить людей к спонтанным и увлекательным занятиям.

В1: Считаете ли вы, что эти доказательства изложены ясно?
R1: полностью согласен/ согласен/ нейтрален/ не согласен/ категорически не согласен
В2: Согласны ли вы с тем, как были изложены эти доказательства?
R2: полностью согласен/ согласен/ нейтрален/ не согласен/ категорически не согласен
В3: Соответствуют ли эти доказательства вашему пониманию, исследованиям и/или практике?
R3: полностью согласен/ согласен/ нейтрален/ не согласен/ категорически не согласен
В4: Комментарии?

... являются катализатором для поддержания здоровья и благополучия

Активные игры на свежем воздухе могут быть полезны для здоровья и благополучия (физического, психического, социального и духовного). [17-25] Игры на свежем воздухе могут предоставить возможность для общения, исследований и участия в здоровых и развивающих занятиях. [17,19,20]

В1: Считаете ли вы, что эти доказательства изложены ясно?
R1: полностью согласен/ согласен/ нейтрален/ не согласен/ категорически не согласен
В2: Согласны ли вы с тем, как были изложены эти доказательства?
R2: полностью согласен/ согласен/ нейтрален/ не согласен/ категорически не согласен
В3: Соответствуют ли эти доказательства вашему пониманию, исследованиям и/или практике?
R3: полностью согласен/ согласен/ нейтрален/ не согласен/ категорически не согласен
В4: Комментарии?

...способствуют разнообразию возможностей для обучения

Активные игры на свежем воздухе могут открыть целый мир увлекательных практических занятий, развивая любознательность, креативность, сотрудничество и способность решать проблемы. [26-29] Это развивает устойчивость и адаптивность, а также поддерживает социальный, эмоциональный и когнитивный рост на протяжении всей жизни. [30-33]

В1: Считаете ли вы, что эти доказательства изложены ясно?
R1: полностью согласен/ согласен/ нейтрален/ не согласен/ категорически не согласен
В2: Согласны ли вы с тем, как были изложены эти доказательства?
R2: полностью согласен/согласен/нейтрально/не согласен/категорически не согласен
В3: Соответствуют ли эти доказательства вашему пониманию, исследованиям и/или практике?
R3: полностью согласен/ согласен/ нейтрален/ не согласен/ категорически не согласен
В4: Комментарии?

...способствуют развитию уверенности, естественным образом

Окружающая среда предоставляет динамичное пространство для игровых приключений и принятия рисков — важный компонент для здорового развития и здорового старения. Занятия сложными активными играми на свежем воздухе помогают развивать уверенность, стойкость и умение решать проблемы, способствуя при этом развитию самостоятельности, благополучия и физической грамотности.[3, 23-25, 34-52]

В1: Считаете ли вы, что эти доказательства изложены ясно?
R1: полностью согласен/ согласен/ нейтрален/ не согласен/ категорически не согласен
В2: Согласны ли вы с тем, как были изложены эти доказательства?
R2: полностью согласен/ согласен/ нейтрален/ не согласен/ категорически не согласен
В3: Соответствуют ли эти доказательства вашему пониманию, исследованиям и/или практике?
R3: полностью согласен/ согласен/ нейтрален/ не согласен/ категорически не согласен
В4: Комментарии?

...открывают путь к более счастливой и здоровой планете

Активные игры на свежем воздухе могут соединить множество сфер, включая здравоохранение, образование, досуг и окружающую среду, при этом учитывая взаимосвязь между благополучием человека, животных и окружающей среды.[53-54] Это способствует охране окружающей среды и укреплению отношений с природой, способствуя формированию более здоровых сообществ, более крепких экосистем и более устойчивой планеты. [1,55-58]

В1: Считаете ли вы, что эти доказательства изложены ясно?
R1: полностью согласен/ согласен/ нейтрален/ не согласен/ категорически не согласен
В2: Согласны ли вы с тем, как были изложены эти доказательства?
R2: полностью согласен/ согласен/ нейтрален/ не согласен/ категорически не согласен
В3: Соответствуют ли эти доказательства вашему пониманию, исследованиям и/или практике?
R3: полностью согласен/ согласен/ нейтрален/ не согласен/ категорически не согласен
В4: Комментарии?

...развивают устойчивость к изменению климата и экологическую ответственность для устойчивого будущего

Активные игры на свежем воздухе способствуют укреплению связи с природой и заботе о нашем окружающем мире. [59-62] Изменение климата может поставить под угрозу возможности для активных игр на свежем воздухе. Когда активные игры на свежем воздухе становятся частью повседневной жизни, это способствует формированию культуры, в которой люди всех возрастов играют, учатся, растут и процветают вместе, наслаждаясь пребыванием на свежем воздухе.

В1: Считаете ли вы, что эти доказательства изложены ясно?
R1: полностью согласен/ согласен/ нейтрален/ не согласен/ категорически не согласен
В2: Согласны ли вы с тем, как были изложены эти доказательства?
R2: полностью согласен/ согласен/ нейтрален/ не согласен/ категорически не согласен
В3: Соответствуют ли эти доказательства вашему пониманию, исследованиям и/или практике?
R3: полностью согласен/ согласен/ нейтрален/ не согласен/ категорически не согласен
В4: Комментарии?

...объединяют сообщества

Совместные активные игры на свежем воздухе способствуют укреплению сообществ, поддержке культурных традиций и усилению разнообразия, одновременно создавая чувство принадлежности. [3, 23-25, 63-70] В свою очередь, сплоченные сообщества создают безопасную и благоприятную среду, которая поощряет гражданскую активность, самостоятельность и активные игры на свежем воздухе для всех.

В1: Считаете ли вы, что эти доказательства изложены ясно?
R1: полностью согласен/ согласен/ нейтрален/ не согласен/ категорически не согласен
В2: Согласны ли вы с тем, как были изложены эти доказательства?
R2: полностью согласен/ согласен/ нейтрален/ не согласен/ категорически не согласен
В3: Соответствуют ли эти доказательства вашему пониманию, исследованиям и/или практике?
R3: полностью согласен/ согласен/ нейтрален/ не согласен/ категорически не согласен
В4: Комментарии?

... помогают сократить чрезмерное времяпрепровождение в помещении

Чрезмерное пребывание в помещении может способствовать продолжительному малоподвижному образу жизни, увеличению времени, проведенного перед экраном, а также подверженности воздействию загрязняющих веществ, аллергенов и инфекционных заболеваний в помещении. [71-78] Активные игры на свежем воздухе обеспечивают здоровый баланс, уменьшая время проведенное в помещении.

В1: Считаете ли вы, что эти доказательства изложены ясно?
R1: полностью согласен/ согласен/ нейтрален/ не согласен/ категорически не согласен
В2: Согласны ли вы с тем, как были изложены эти доказательства?
R2: полностью согласен/ согласен/ нейтрален/ не согласен/ категорически не согласен
В3: Соответствуют ли эти доказательства вашему пониманию, исследованиям и/или практике?
R3: полностью согласен/ согласен/ нейтрален/ не согласен/ категорически не согласен
В4: Комментарии?

Новые направления: расширение возможностей для активных игр на свежем воздухе

По мере того, как мы расширяем границы того, что известно об активных играх на свежем воздухе, возникают новые и неотложные вопросы, которые бросают вызов традиционному мышлению, вызывают любопытство и открывают двери для преобразующих изменений. Следующие области представляют собой неизведанные территории, где исследования, политика и практика должны сойтись, чтобы сформировать будущее активных игр на свежем воздухе.

🌍 Могут ли активные игры на свежем воздухе изменить двигательные паттерны взрослых? Как физическая активность, малоподвижный образ жизни и режим сна взаимодействуют с активными играми на свежем воздухе у взрослых? Могут ли игры на свежем воздухе стать ключом к улучшению физического и психического благополучия на протяжении всей жизни?

🧠 Могут ли рискованные игры на свежем воздухе стать ключевым фактором для академического роста? Могут ли рискованные игры на свежем воздухе — забираться выше, бегать быстрее, исследовать неизведанное — на самом деле усилить когнитивные функции и успеваемость?

🌏 Как выглядят активные игры на свежем воздухе за пределами Глобального Севера? Как различные культурные, экологические и социально-политические контексты формируют способы, которыми люди занимаются активными играми на свежем воздухе? Что мы можем сделать, чтобы узнать и поделиться лучшими практиками из разных регионов и культур?

🔬 Как преодолеть разрыв между исследованиями и реальными играми? Чего не хватает между тем, что мы изучаем, и тем, что происходит на местах? Как исследования могут лучше информировать политику и практику, которые поддерживают активные игры на свежем воздухе?

🏫 Можно ли перепроектировать и перепрограммировать школы и школьные дворы в игровые площадки для движения, творчества и обучения? Как можно интегрировать активные игры на свежем воздухе в формальные образовательные системы — не просто как для перерыва в обучении, а как его неотъемлемую часть? Как будет выглядеть школа, построенная для движения и игр? Могут ли школьные дворы служить более важной цели, становясь общественными игровыми площадками на свежем воздухе?

🏙 Как мы проектируем города, которые приглашают к игре на каждом шагу? От биоразнообразных городских парков до здоровых и игровых улиц, какие особенности городского дизайна делают активные игры на свежем воздухе неотразимыми и доступными? Инклюзивными для всех?

🌱 Могут ли активные игры на свежем воздухе стать проводником глобальной устойчивости? Что, если активные игры на свежем воздухе станут ключом к решению некоторых из самых больших мировых проблем — изменения климата, неравенства в области здоровья и социальной сферы? Как это согласуется с Целями ООН в области устойчивого развития и какая политика может усилить это влияние?

📱 С ростом беспокойства по поводу времени, проводимого перед экранами, тревожности и одиночества, могут ли активные игры на свежем воздухе стать мощным противоядием от негативных последствий использования социальных сетей? Как мы можем вернуть движение, природу и личное общение в эпоху цифровых технологий?

Каждый из этих вопросов несет в себе потенциал для разрушения общепринятых взглядов и переосмысления роли игр на свежем воздухе в формировании более здоровых, более устойчивых людей и сообществ. Следующий шаг? Смелые исследования, инновационная политика и приверженность действию!

В1: Считаете ли вы, что этот раздел изложен ясно?
R1: полностью согласен/ согласен/ нейтрален/ не согласен/ категорически не согласен
В2: Согласны ли вы с тем, как был изложен этот раздела?
R2: полностью согласен/ согласен/ нейтрален/ не согласен/ категорически не согласен
В3: Соответствует ли этот раздел вашему пониманию, исследованиям и/или практике?
R3: полностью согласен/ согласен/ нейтрален/ не согласен/ категорически не согласен
В4: Комментарии?

Призыв к действию: рекомендации по продвижению активных игр на свежем воздухе

Общественные действия

- Поощряйте культуру, в которой ценятся и отдаются приоритеты активным играм на свежем воздухе как части повседневной жизни.
- Создавайте и улучшайте доступ к пространствам, где каждый может наслаждаться активными играми на свежем воздухе.
- Выступайте за сотрудничество между исследователями, педагогами, градостроителями, специалистами в области здравоохранения и политиками, чтобы сделать активные игры на свежем воздухе приоритетом для здоровья.

Политика и законодательство

- Признайте доступ к активным играм на свежем воздухе как основополагающее право в политике в области здравоохранения, образования, отдыха и охраны окружающей среды.
- Поддерживайте правительства в принятии и поддержке политики, способствующей активным играм на свежем воздухе.
- Продвигайте, защищайте, сохраняйте и инвестируйте в игровые площадки на открытом воздухе, которые объединяют районы, школы, зоны отдыха и рабочие места.

Образование и школы

- Поддерживайте колледжи, центры образования для взрослых и общественные учебные центры, чтобы они включали в программы непрерывное профессиональное развитие, связанное с активными играми и обучением на свежем воздухе.
- Требуйте ежедневных активных игр на свежем воздухе в дошкольном образовании и школьной политике для учащихся с 1 до 12 класса.
- Интегрируйте занятия на свежем воздухе и обучение на основе природы в образовательные системы.

Общественное здоровье и здравоохранение

- Просвещайте медицинских работников, пациентов и общественность о пользе для здоровья активных игр на свежем воздухе.
- Интегрируйте активные игры на свежем воздухе в практику здравоохранения и инициативы общественного здравоохранения для снижения малоподвижного образа жизни и улучшения здоровья.
- Сотрудничайте между секторами для разработки локальных стратегий общественного здравоохранения, поощряющих активные игры на свежем воздухе.

Городское планирование

- Проектируйте доступные, безопасные и удобные для игр открытые пространства в жилых кварталах и вокруг них.
- Отдавайте приоритет сохранению и восстановлению природной среды, способствующей активным играм на свежем воздухе.
- Реформируйте муниципальную политику и подзаконные акты для активной поддержки и создания условий для активных игр на свежем воздухе.

Лидерство в сообществе

- Поддерживайте, продвигайте и развивайте кампании, подчеркивающие важность активных игр на свежем воздухе как привычки, способствующей укреплению здоровья.
- Поддерживайте, поощряйте и развивайте усилия, которые поощряют подходы к активным играм на свежем воздухе, основанные на соотношении пользы и риска.
- Поощряйте активные игры на свежем воздухе между представителями разных поколений для укрепления связей в обществе.

Исследования и наблюдение

- Инвестируйте в системы сбора и мониторинга данных, чтобы отслеживать тенденции в сфере активных игр на свежем воздухе и выявлять пробелы.
- Изучайте оптимальное качество и количество активных игр на свежем воздухе для здоровых людей и сообществ.
- Устанавливайте причинно-следственные связи между активными играми на свежем воздухе и результатами в отношении здоровья и благополучия.

Семьи

- Играйте с другими людьми и с домашними животными, чтобы развивать чувство общности и связи с природой.
- Поощряйте и демонстрируйте активные игры на свежем воздухе как нормативную модель поведения в вашем районе.
- Поддерживайте участие членов семьи в активных играх на свежем воздухе, поощряя, содействуя и вовлекая их в совместные игры.

Лица

- Будьте уважительными хозяевами открытого пространства, где происходят игры.
- Выступайте за равный доступ и сохранение зеленых насаждений и безопасной игровой среды.
- Исследуйте и наслаждайтесь разнообразными впечатлениями в различных местах на свежем воздухе, сделав их частью своей повседневной жизни.

B1: Считаете ли вы, что этот раздел изложен ясно?
R1: полностью согласен/ согласен/ нейтрален/ не согласен/ категорически не согласен
B2: Согласны ли вы с тем, как был изложен этот раздела?
R2: полностью согласен/ согласен/ нейтрален/ не согласен/ категорически не согласен
B3: Соответствует ли этот раздел вашему пониманию, исследованиям и/или практике?
R3: полностью согласен/ согласен/ нейтрален/ не согласен/ категорически не согласен
B4: Комментарии?

Другие/дополнительные отзывы

1. Считаете ли вы, что это обновление позиционного заявления об активных играх на свежем воздухе важно для общественного здравоохранения?

a. Да

b. Нет

c. Не уверен

2. Считаете ли вы, что это обновление позиционного заявления об активных играх на свежем воздухе важно для глобального и планетарного здоровья?

a. Да

b. Нет

c. Не уверен

3. Считаете ли вы, что это обновление позиционного заявления об активных играх на свежем воздухе важно для достижения Целей устойчивого развития Организации Объединенных Наций?

a. Да

b. Нет

c. Не уверен

4. Считаете ли вы, что это обновление позиционного заявления об активных играх на свежем воздухе важно для вас и/или вашей работы?

a. Да

b. Нет

c. Не уверен

5. Считаете ли вы это позиционное заявление актуальным/применимым к стране, в которой вы проживаете?

a. Да

b. Нет

c. Не уверен

6. В поле ниже введите любые дополнительные комментарии, которые вы хотели бы добавить относительно этого обновления позиционного заявления об активных играх на свежем воздухе:

(open box)

Демографическая информация

7. Возраст

a. <35 лет

b. 35-55 лет

c. >55 лет

d. Предпочитаю не отвечать

8. Какую сферу вы представляете/в какой сфере работаете?

a. Дошкольное образование

b. Начальная и средняя школа

c. Медицинский работник

d. Смежный медицинский работник (например, специалист по общественному здравоохранению, физиотерапевт, медсестра, диетолог, эрготерапевт)

e. Специалист по фитнесу (например, кинезиолог)

f. Профилактика травм

g. Страхование

h. Право

i. Профессор/исследователь (научный сотрудник)

j. Сотрудник или член правления неправительственной организации

k. Правительство

l. Другое, укажите:

9. В какой стране вы в основном живете, работаете и отдыхаете?

(drop down menu list of countries)

Признание вашего вклада

Когда будет готова окончательная версия этого позиционного заявления об активных играх на свежем воздухе, хотели бы вы, чтобы с вами связались для получения окончательной версии и, в случае поддержки, быть указанными как сторонник этого позиционного заявления?

a. Да

b. Нет

c. Не уверен

Если да, введите свой адрес электронной почты здесь:

____

*Примечание: эта информация не связана с опросом о консенсусе. Ваши ответы на опрос о консенсусе останутся анонимными, если вы укажете свой адрес электронной почты.

1. Ingrese el código de acceso que se le proporcionó con el enlace de la encuesta.

Posicionamiento sobre el Juego Activo al aire libre 2025–Encuesta de Consenso Global

Preámbulo

El Posicionamiento de 2015 sobre el Juego Activo al Aire Libre[1] y la evidencia que la respalda [2-3] demostraron los beneficios del juego activo al aire libre para la salud y el bienestar infantil. Diez años después, la investigación sobre este tema se ha multiplicado por diez. [4] Ha aumentado la financiación para proyectos de juego al aire libre. [5] El Posicionamiento ha influido en políticas, investigaciones y prácticas a nivel mundial. [6] Un grupo internacional de liderazgo se ha unido para crear el Posicionamiento de 2025 sobre el Juego Activo al Aire Libre con el fin de celebrar estos logros, actualizar la evidencia y ampliar su alcance. Por ejemplo, el Posicionamiento de 2025 es inclusivo para todas las edades y tiene un alcance global.

El Posicionamiento de 2025 visualiza un mundo donde el juego activo al aire libre contribuya a abordar desafíos globales como las crisis sanitarias y el cambio climático, a la vez que promueve los Objetivos de Desarrollo Sostenible de las Naciones Unidas. [7] Juntos, como colectivo del sector del juego al aire libre, queremos construir comunidades más saludables y resilientes integrando el juego activo al aire libre en la vida diaria, garantizando que todos tengan acceso equitativo y oportunidades para participar en él.

Este Posicionamiento convoca a un cambio sistémico y ofrece recomendaciones para individuos, comunidades y sectores. Estas recomendaciones buscan convertir el juego activo al aire libre en un derecho fundamental y una parte esencial de las sociedades sostenibles.

Para apoyar este trabajo, hemos realizado 10 revisiones sistemáticas y seis revisiones narrativas continentales, analizamos la literatura relevante y hemos consultado a expertos mundiales sobre nueve temas clave:

- *salud y bienestar*
- *Una sola salud*
- *la naturaleza y el medio ambiente*
- *derechos humanos y políticas*
- *conexiones comunitarias y alianzas*
- *capital social*
- *educación y aprendizaje*
- *comportamientos de movimiento*
- *áreas emergentes*

Consulte los enlaces al marco conceptual de este proyecto y una lista completa de referencias en la parte inferior de esta página.

P1: ¿Considera que esta sección está claramente redactada?
R1: totalmente de acuerdo/ de acuerdo/ neutral/ en desacuerdo/ totalmente en desacuerdo

P2: ¿Está usted de acuerdo con la forma como se enunció esta sección?
R2: totalmente de acuerdo/ de acuerdo/ neutral/ en desacuerdo/ totalmente en desacuerdo
P3: ¿Esta sección se alinea con su comprensión, investigación y/o práctica?
R3: totalmente de acuerdo/ de acuerdo/ neutral/ en desacuerdo/ totalmente en desacuerdo

A continuación, la propuesta de Posicionamiento de 2025:

El juego activo al aire libre promueve la salud y el bienestar holístico para personas de todas las edades, comunidades, entornos y del planeta entero. Es crucial dados los múltiples desafíos globales que enfrentamos hoy (p. ej., desigualdades sociales y sanitarias, cambio climático). Juntos, como colectivo del sector del juego al aire libre, recomendamos aumentar las oportunidades de juego activo al aire libre en todos los entornos donde las personas viven, aprenden, trabajan y se divierten. Para lograrlo, es importante colaborar entre sectores, entornos y sociedades para preservar, promover y valorar el acceso equitativo al juego activo al aire libre y en la naturaleza.

P1: ¿Considera que esta sección está claramente redactada?
R1: totalmente de acuerdo/ de acuerdo/ neutral/ en desacuerdo/ totalmente en desacuerdo
P2: ¿Está usted de acuerdo con la forma como se enunció esta sección?
R2: totalmente de acuerdo/ de acuerdo/ neutral/ en desacuerdo/ totalmente en desacuerdo
P3: ¿Esta sección se alinea con su comprensión, investigación y/o práctica?
R3: totalmente de acuerdo/ de acuerdo/ neutral/ en desacuerdo/ totalmente en desacuerdo
P4: ¿Comentarios?

[A partir de esta página, presentamos información clave derivada del trabajo realizado para cada tema. El objetivo es que sea atractiva e informativa. Después de cada afirmación, el párrafo descriptivo proporciona más contexto con referencias para destacar que la información está respaldada por evidencia. Se agradecen sugerencias de referencias adicionales.]

El juego activo al aire libre…

…nos ayuda a movernos más, jugar más tiempo y dormir mejor

Cuando jugamos al aire libre, realizamos más actividad física y menos tiempo en actividades sedentarias, como, por ejemplo, pasar menos tiempo frente a la pantalla, lo que nos lleva a dormir mejor. [8-16] Los entornos al aire libre pueden motivar a las personas a participar en actividades espontáneas y divertidas.

P1: ¿Considera que esta sección está claramente redactada?
R1: totalmente de acuerdo/ de acuerdo/ neutral/ en desacuerdo/ totalmente en desacuerdo
P2: ¿Está usted de acuerdo con la forma como se enunció esta sección?
R2: totalmente de acuerdo/ de acuerdo/ neutral/ en desacuerdo/ totalmente en desacuerdo
P3: ¿Esta sección se alinea con su comprensión, investigación y/o práctica?
R3: totalmente de acuerdo/ de acuerdo/ neutral/ en desacuerdo/ totalmente en desacuerdo
P4: ¿Comentarios?

...es un catalizador para mantener la salud y el bienestar

El juego activo al aire libre puede mejorar la salud y el bienestar (es decir, la salud física, mental, social y espiritual). [17-25] Jugar al aire libre puede ofrecer una oportunidad para conectar, explorar e interactuar en opciones más saludables y enriquecedoras. [17,19,20]

P1: ¿Considera que esta sección está claramente redactada?
R1: totalmente de acuerdo/ de acuerdo/ neutral/ en desacuerdo/ totalmente en desacuerdo
P2: ¿Está usted de acuerdo con la forma como se enunció esta sección?
R2: totalmente de acuerdo/ de acuerdo/ neutral/ en desacuerdo/ totalmente en desacuerdo
P3: ¿Esta sección se alinea con su comprensión, investigación y/o práctica?
R3: totalmente de acuerdo/ de acuerdo/ neutral/ en desacuerdo/ totalmente en desacuerdo
P4: ¿Comentarios?

...facilita diversas oportunidades de aprendizaje

El juego activo al aire libre puede abrir un mundo de experiencias de aprendizaje enriquecedoras y prácticas, así como fomentar la curiosidad, creatividad, colaboración y resolución de problemas. [26-29] Además, promueve la resiliencia y la adaptabilidad, y apoya el crecimiento social, emocional y cognitivo a lo largo de la vida. [30-33]

P1: ¿Considera que esta sección está claramente redactada?
R1: totalmente de acuerdo/ de acuerdo/ neutral/ en desacuerdo/ totalmente en desacuerdo
P2: ¿Está usted de acuerdo con la forma como se enunció esta sección?
R2: totalmente de acuerdo/ de acuerdo/ neutral/ en desacuerdo/ totalmente en desacuerdo
P3: ¿Esta sección se alinea con su comprensión, investigación y/o práctica?
R3: totalmente de acuerdo/ de acuerdo/ neutral/ en desacuerdo/ totalmente en desacuerdo
P4: ¿Comentarios?

... promueve la confianza, naturalmente

Los entornos al aire libre brindan un espacio dinámico para aventuras lúdicas y arriesgarse - un ingrediente esencial para un desarrollo y envejecimiento saludables. Participar en juegos al aire libre que sean desafiantes ayuda a desarrollar la confianza, la resiliencia y las habilidades para resolver problemas, al tiempo que promueve la autonomía, el bienestar, y la alfabetización física. [3, 23-25, 34-52]

P1: ¿Considera que esta sección está claramente redactada?
R1: totalmente de acuerdo/ de acuerdo/ neutral/ en desacuerdo/ totalmente en desacuerdo
P2: ¿Está usted de acuerdo con la forma como se enunció esta sección?
R2: totalmente de acuerdo/ de acuerdo/ neutral/ en desacuerdo/ totalmente en desacuerdo
P3: ¿Esta sección se alinea con su comprensión, investigación y/o práctica?
R3: totalmente de acuerdo/ de acuerdo/ neutral/ en desacuerdo/ totalmente en desacuerdo
P4: ¿Comentarios?

...ofrece un camino hacia un planeta más feliz y saludable

El juego activo al aire libre puede tender un puente entre múltiples sectores, incluyendo la salud pública, la educación, la recreación, y el medio ambiente, mientras se reconoce la interconexión del bienestar humano, animal y ambiental. [53-54]

Fomenta la protección ambiental y fortalece las relaciones con la naturaleza, contribuyendo a crear comunidades más saludables, ecosistemas más fuertes, y un planeta más sostenible. [1,55-58]

P1: ¿Considera que esta sección está claramente redactada?
R1: totalmente de acuerdo/ de acuerdo/ neutral/ en desacuerdo/ totalmente en desacuerdo
P2: ¿Está usted de acuerdo con la forma como se enunció esta sección?
R2: totalmente de acuerdo/ de acuerdo/ neutral/ en desacuerdo/ totalmente en desacuerdo
P3: ¿Esta sección se alinea con su comprensión, investigación y/o práctica?
R3: totalmente de acuerdo/ de acuerdo/ neutral/ en desacuerdo/ totalmente en desacuerdo
P4: ¿Comentarios?

...construye resiliencia climática y gestión para un futuro sostenible

La participación en el juego activo al aire libre fomenta una conexión más profunda con la naturaleza y el cuidado de nuestro entorno natural.[59-62] El cambio climático puede amenazar las oportunidades de juego activo al aire libre. Cuando el juego activo al aire libre forma parte de la vida diaria, puede fomentar una cultura donde personas de todas las edades jueguen, aprendan, crezcan y prosperen juntos mientras disfrutamos de los espacios al aire libre.

P1: ¿Considera que esta sección está claramente redactada?
R1: totalmente de acuerdo/ de acuerdo/ neutral/ en desacuerdo/ totalmente en desacuerdo
P2: ¿Está usted de acuerdo con la forma como se enunció esta sección?
R2: totalmente de acuerdo/ de acuerdo/ neutral/ en desacuerdo/ totalmente en desacuerdo
P3: ¿Esta sección se alinea con su comprensión, investigación y/o práctica?
R3: totalmente de acuerdo/ de acuerdo/ neutral/ en desacuerdo/ totalmente en desacuerdo
P4: ¿Comentarios?

...conecta comunidades

Las experiencias compartidas de juego activo al aire libre construyen comunidades más fuertes, celebran las tradiciones culturales y fortalecen la diversidad, fomentando un sentido de pertenencia. [3, 23-25, 63-70] A su vez, las comunidades cohesionadas crean entornos seguros y de apoyo que fomentan el civismo, la iniciativa y el juego activo al aire libre para todos.

P1: ¿Considera que esta sección está claramente redactada?
R1: totalmente de acuerdo/ de acuerdo/ neutral/ en desacuerdo/ totalmente en desacuerdo
P2: ¿Está usted de acuerdo con la forma como se enunció esta sección?
R2: totalmente de acuerdo/ de acuerdo/ neutral/ en desacuerdo/ totalmente en desacuerdo
P3: ¿Esta sección se alinea con su comprensión, investigación y/o práctica?
R3: totalmente de acuerdo/ de acuerdo/ neutral/ en desacuerdo/ totalmente en desacuerdo
P4: ¿Comentarios?

...puede reducir el tiempo excesivo en interiores

El exceso de tiempo en interiores puede contribuir al comportamiento sedentario prologando, al aumento del tiempo frente a pantallas, y a la exposición de contaminantes de interior, alérgenos y enfermedades infecciosas de interiores. [71-78] El juego activo al aire libre proporciona un equilibrio saludable al reducir el tiempo que se pasa en espacios interiores.

P1: ¿Considera que esta sección está claramente redactada?
R1: totalmente de acuerdo/ de acuerdo/ neutral/ en desacuerdo/ totalmente en desacuerdo
P2: ¿Está usted de acuerdo con la forma como se enunció esta sección?
R2: totalmente de acuerdo/ de acuerdo/ neutral/ en desacuerdo/ totalmente en desacuerdo
P3: ¿Esta sección se alinea con su comprensión, investigación y/o práctica?
R3: totalmente de acuerdo/ de acuerdo/ neutral/ en desacuerdo/ totalmente en desacuerdo
P4: ¿Comentarios?

Áreas emergentes: Ampliando las posibilidades del Juego Activo al Aire Libre

A medida que ampliamos los conocimientos sobre el juego activo al aire libre, surgen nuevas y urgentes preguntas que desafían el pensamiento convencional, despiertan la curiosidad y abren puertas a un cambio transformador. Las siguientes áreas representan territorios inexplorados donde la investigación, las políticas y la práctica deben converger para definir el futuro del juego activo al aire libre.

¿Puede el juego activo al aire libre transformar los patrones de movimiento de los adultos? ¿Cómo interactúan la actividad física, el sedentarismo y los patrones de sueño con el juego activo al aire libre en adultos? ¿Podría el juego al aire libre ser la clave para mejorar el bienestar físico y mental a lo largo de la vida?

¿Podría el juego arriesgado al aire libre ser un ingrediente clave para el crecimiento académico? ¿Podría arriesgarse al aire libre (subir más alto, correr más rápido, explorar lo desconocido) fortalecer la función cognitiva y el rendimiento académico?

¿Cómo se ve el juego activo al aire libre más allá del hemisferio norte? ¿Cómo influyen los diferentes contextos culturales, ambientales y sociopolíticos en la forma en que las personas participan en el juego activo al aire libre? ¿Qué podemos hacer para aprender y compartir las mejores prácticas de diferentes regiones y culturas?

¿Cómo acortamos la distancia entre la investigación y el juego en la vida real? ¿Qué falta entre lo que estudiamos y lo que sucede en la práctica? ¿Cómo puede la investigación fundamentar mejor las políticas y prácticas que apoyan el juego activo al aire libre?

¿Es posible rediseñar y reprogramar las escuelas y los patios escolares como espacios de juego ara el movimiento, la creatividad y el aprendizaje? ¿Cómo podemos integrar el juego activo al aire libre en los sistemas educativos formales, no solo como un descanso del aprendizaje, sino como parte esencial del mismo? ¿Cómo sería una escuela diseñada para el movimiento y el juego? ¿Podrían los patios escolares cumplir una función más importante como espacios comunitarios de juego al aire libre?

¿Cómo diseñamos ciudades que inviten al juego en cada rincón? Desde parques urbanos con biodiversidad hasta calles saludables y lúdicas, ¿qué características del diseño urbano hacen que el juego activo al aire libre sea irresistible y accesible? ¿Incluyente para todos?

¿Podría el juego activo al aire libre ser un vehículo para la sostenibilidad global? ¿Qué pasaría si el juego activo al aire libre fuera la clave para abordar algunos de los mayores desafíos del mundo: el cambio climático y las desigualdades sociales y sanitarias? ¿Cómo se alinea con los Objetivos de Desarrollo Sostenible de la ONU y qué políticas podrían potenciar su impacto?

Ante la creciente preocupación por el tiempo frente a las pantallas, la ansiedad y la soledad, ¿podría el juego activo al aire libre ser un potente antídoto contra los efectos negativos del uso de las redes sociales? ¿Cómo podemos reintroducir el movimiento, la naturaleza y la conexión cara a cara en la era digital?

Cada una de estas preguntas tiene el potencial de romper con la creencia popular y volver a imaginar el papel del juego al aire libre en el desarrollo de individuos y comunidades más saludables y resilientes. ¿El siguiente paso? ¡Investigación audaz, políticas innovadoras y un compromiso con la acción!

P1: ¿Considera que esta sección está claramente redactada?
R1: totalmente de acuerdo/ de acuerdo/ neutral/ en desacuerdo/ totalmente en desacuerdo
P2: ¿Está usted de acuerdo con la forma como se enunció esta sección?
R2: totalmente de acuerdo/ de acuerdo/ neutral/ en desacuerdo/ totalmente en desacuerdo
P3: ¿Esta sección se alinea con su comprensión, investigación y/o práctica?
R3: totalmente de acuerdo/ de acuerdo/ neutral/ en desacuerdo/ totalmente en desacuerdo
P4: ¿Comentarios?

Un llamado a la acción: Recomendaciones para Promover el Juego Activo al Aire Libre

Acciones sociales

- Fomentar una cultura que valore y priorice el juego activo al aire libre como parte de la vida diaria.
- Crear y mejorar el acceso a espacios donde todos puedan disfrutar del juego activo al aire libre.
- Abogar por la colaboración entre investigadores, educadores, planificadores urbanos, profesionales de la salud y formuladores de políticas para hacer del juego activo al aire libre una prioridad de salud.

Política y legislación

- Reconocer el acceso al juego activo al aire libre como un derecho fundamental en las políticas de salud, educación, recreación y medio ambiente.
- Instar a los gobiernos a promulgar y mantener políticas que apoyen el juego activo al aire libre.

- Promover, proteger, preservar e invertir en entornos de juego al aire libre que conecten vecindarios, escuelas, áreas recreativas y lugares de trabajo.

Educación y escuelas
- Alentar a las universidades, centros de educación para adultos y centros de aprendizaje comunitarios a integrar en sus programas el desarrollo profesional continúo relacionado con el juego y el aprendizaje activos al aire libre.
- Exigir tiempo de juego activo al aire libre todos los días en la educación de la primera infancia y en las políticas escolares.
- Integrar aulas al aire libre y aprendizaje basado en la naturaleza en los sistemas educativos.

Salud pública y Atención Sanitaria
- Educar a los profesionales médicos, pacientes y comunidades sobre los beneficios para la salud del juego activo al aire libre.
- Integrar el juego activo al aire libre en las prácticas de atención médica y en las iniciativas de salud pública para reducir el comportamiento sedentario y mejorar la salud.
- Colaborar entre sectores para desarrollar estrategias de salud pública localizadas que promuevan el juego activo al aire libre.

Planificación Urbana
- Diseñar espacios al aire libre accesibles, seguros y propicios para el juego en los vecindarios y sus alrededores.
- Priorizar la preservación y restauración de entornos naturales que fomenten el juego activo al aire libre al diseñar o rediseñar comunidades.
- Reformar las políticas y estatutos municipales para apoyar y permitir activamente el juego activo al aire libre.

Liderazgo Comunitario
- Apoyar, promover y desarrollar campañas que enfaticen la importancia del juego activo al aire libre como un hábito que promueve la salud.
- Apoyar, promover y desarrollar esfuerzos que promuevan enfoques de beneficios y riesgos para el juego activo al aire libre.
- Fomentar el juego activo al aire libre intergeneracional para fortalecer las conexiones comunitarias.

Investigación y Vigilancia
- Invertir en sistemas de recopilación y monitorización de datos para seguir las tendencias en el juego activo al aire libre e identificar brechas.
- Explorar la calidad y cantidad óptimas de juego activo al aire libre para personas y comunidades saludables.
- Establecer vías causales entre el juego activo al aire libre y los resultados de salud y bienestar.

Familias

- Jugar con otros, incluidos los animales de compañía, para fomentar un sentido de comunidad y conexión con el aire libre.
- Fomentar y modelar el juego activo al aire libre como un comportamiento normativo en su vecindario.
- Apoyar la participación de los miembros de la familia en juegos activos al aire libre alentándolos, ayudándolos a hacerlo posible, y comprometiéndose juntos.

Individuos

- Ser guardianes respetuosos de los entornos al aire libre donde se desarrolla el juego.
- Abogar por el acceso equitativo y la preservación de espacios verdes y entornos seguros y propicios para el juego.
- Explorar y disfrutar de diversas experiencias en diferentes espacios al aire libre como parte de tu rutina diaria.

P1: ¿Considera que esta sección está claramente redactada?
R1: totalmente de acuerdo/ de acuerdo/ neutral/ en desacuerdo/ totalmente en desacuerdo
P2: ¿Está usted de acuerdo con la forma como se enunció esta sección?
R2: totalmente de acuerdo/ de acuerdo/ neutral/ en desacuerdo/ totalmente en desacuerdo
P3: ¿Esta sección se alinea con su comprensión, investigación y/o práctica?
R3: totalmente de acuerdo/ de acuerdo/ neutral/ en desacuerdo/ totalmente en desacuerdo
P4: ¿Comentarios?

Otros comentarios

1. ¿Considera que esta actualización del Posicionamiento sobre Juego Activo al Aire Libre es importante para la salud pública?

a. Sí

b. No

c. No estoy seguro

2. ¿Considera que esta actualización del Posicionamiento sobre Juego Activo al Aire Libre es importante para la salud global y planetaria?

a. Sí

b. No

c. No estoy seguro

3. ¿Considera que esta actualización del Posicionamiento sobre Juego Activo al Aire Libre es importante para alcanzar los Objetivos de Desarrollo Sostenible de las Naciones Unidas?

a. Sí

b. No

c. No estoy seguro

4. ¿Considera que esta actualización del Posicionamiento sobre Juego Activo al Aire Libre es importante para usted o su trabajo?

a. Sí

b. No

c. No estoy seguro

5. ¿Considera que el Posicionamiento es relevante o aplicable a su país de residencia?

a. Sí

b. No

c. No estoy seguro

6. En el recuadro a continuación, escriba cualquier comentario adicional que desee agregar sobre esta actualización del Posicionamiento sobre el Juego Activo al Aire Libre:

(pregunta abierta)

Información Demográfica

7. Edad

a. <35 años

b. 35-55 años

c. >55 años

d. Prefiero no responder

8. ¿En qué sector trabaja o a qué sector representa?

a. Educación infantil

b. Educación primaria y secundaria

c. Profesional de la salud

d. Profesional de la salud afín (p. ej., profesional de la salud pública, fisioterapeuta, enfermero/a, dietista, terapeuta ocupacional)

e. Profesional del fitness (p. ej., kinesiólogo/a, entrenador/a personal)

f. Prevención de lesiones

g. Seguros

h. Derecho

i. Profesor/a/Investigador/a

j. Personal o miembro de la junta directiva de una organización no gubernamental

k. Gobierno

l. Otro (especifique):

9. ¿En qué país vive, trabaja y se divierte principalmente?

(seleccione de la lista de países)

Reconocimiento a su contribución

Una vez finalizada la versión final del Posicionamiento sobre el Juego Activo al Aire Libre, ¿le gustaría que nos pusiéramos en contacto con usted para recibirla y, si la apoya, que se le incluyera como partidario del Posicionamiento?

a. Sí

b. No

c. No estoy seguro

En caso afirmativo, introduzca su correo electrónico aquí:

____

*Nota: Esta información no está relacionada con la encuesta de consenso. Sus respuestas a la encuesta de consenso se mantendrán anónimas si proporciona su correo electrónico.
